# Gene cascade analysis in human granulosa tumor cells (KGN) following exposure to high levels of free fatty acids and insulin

**DOI:** 10.1186/s13048-021-00934-6

**Published:** 2021-12-20

**Authors:** Patricia G. Tremblay, Chloé Fortin, Marc-André Sirard

**Affiliations:** Centre de Recherche en Reproduction, Développement et Santé Intergénérationnelle, Faculté des Sciences de l’Agriculture et de l’Alimentation, Département des Sciences Animales, Université Laval, Québec, QC G1V 0A6 Canada

**Keywords:** Granulosa cells, Ovary, Signaling, Insulin signaling, Free fatty acid signaling, Metabolic disease

## Abstract

Maternal metabolic disorders such as obesity and diabetes are detrimental factors that compromise fertility and the success rates of medically assisted procreation procedures. During metabolic stress, adipose tissue is more likely to release free fatty acids (FFA) in the serum resulting in an increase of FFA levels not only in blood, but also in follicular fluid (FF). In humans, high concentrations of palmitic acid and stearic acid reduced granulosa cell survival and were associated with poor cumulus-oocyte complex (COC) morphology. Obesity and high levels of circulating FFA were also causatively linked to hampered insulin sensitivity in cells and compensatory hyperinsulinemia. To provide a global picture of the principal upstream signaling pathways and genomic mechanisms involved in this metabolic context, human granulosa-like tumor cells (KGN) were treated with a combination of palmitic acid, oleic acid, and stearic acid at the higher physiological concentrations found in the follicular fluid of women with a higher body mass index (BMI) (≥ 30.0 kg/m^2^). We also tested a high concentration of insulin alone and in combination with high concentrations of fatty acids. Transcription analysis by RNA-seq with a cut off for fold change of 1.5 and p-value 0.05 resulted in thousands of differentially expressed genes for each treatment. Using analysis software such as Ingenuity Pathway Analysis (IPA), we were able to establish that high concentrations of FFA affected the expression of genes mainly related to glucose and insulin homoeostasis, fatty acid metabolism, as well as steroidogenesis and granulosa cell differentiation processes. The combination of insulin and high concentrations of FFA affected signaling pathways related to apoptosis, inflammation, and oxidative stress. Taken together, our results provided new information on the mechanisms that might be involved in human granulosa cells exposed to high concentrations of FFA and insulin in the contexts of metabolism disorders.

**Supplementary Information:**

The online version contains supplementary material available at 10.1186/s13048-021-00934-6.

## Background

The increasing prevalence of metabolic syndrome worldwide and its association with adverse health effects, including impaired reproductive function in women, have become issues of concern over the past decade. Maternal metabolic disorders such as obesity and diabetes are detrimental factors that compromise fertility and the success rates of medically assisted procreation (MAP) procedures [1, 2]. Accumulating evidence in several animal studies, especially the negative energy balance (NEB) model in bovine, contributed to indicate that free fatty acids (FFA) are key molecules associated with impaired cumulus-oocyte-complex (COC) morphology and impaired oocyte developmental competence and metabolism [3–11]. Free fatty acids act as cellular energy sources, essential components in membrane biogenesis, are involved in granulosa cell steroidogenesis, in addition to being powerful signaling molecules [12, 13]. Elevated levels of FFA in circulation is a major characteristic of metabolic stress in human and bovine [5, 6, 9, 14, 15]. During metabolic stress such as obesity or diabetes, adipose tissue is more likely to release fatty acids in the serum resulting in increased FFA levels, not only in blood, but also in follicular fluid (FF) [11]. Recent studies in human and bovine showed that levels of FFA in blood were correlated with concentrations of FFA in follicular fluid [5, 6, 9, 16–18]. Analysis of fatty acids (FA) in human follicular fluid lipid fractions showed that FFA reflected the Body Mass Index (BMI) in women which was positively correlated with increasing FFA in ovarian follicular fluid [3, 15]. Palmitic acid (C16:0) (PA), stearic acid (C18:0) (SA), and (C18:1) oleic acid (OA) were among the most abundant FFA in lipid fractions of human and bovine FF [5, 6, 15]. In humans, high concentrations of PA and SA reduced granulosa cell survival through the metabolism of the acyl-CoA forms [7]. Moreover, the COC of women with higher follicular FFA at oocyte aspiration tended to have poor morphology [5]. In bovine models, elevated concentrations of non-esterified fatty acids (NEFA), including saturated PA and SA, jeopardized granulosa cell viability and reduced oocyte developmental competence [3, 4, 6, 8, 10, 14, 19, 20]. Obesity and high levels of circulating NEFA were also causatively linked to hampered insulin sensitivity in cells and compensatory hyperinsulinemia [21, 22]. A higher BMI in women was significantly associated with increased levels of insulin in FF [15, 18]. As reviewed by Purcell and Moley, insulin is involved in various granulosa cell functions such as proliferation, apoptosis, and steroidogenesis [23]. Moreover, periconceptional hyperinsulinemia is associated with obesity and may impair several granulosa cell functions such as estradiol production and FSHR expression, thereby impairing the response to fertility treatments [24].

Studying the specific effects of NEFA is a very difficult task using in vivo experimentation as follicular dynamics result in a constantly changing transcriptome. Therefore, to provide a global picture of the principal upstream signaling pathways and genomic mechanisms involved in the metabolic contexts described above, human granulosa-like tumor cells (KGN) were treated with a combination of PA, OA, and SA at the higher physiological concentrations found in the follicular fluid of women with higher BMI (≥ 30.0 kg/m^2^) [15]. We also tested a high concentration of insulin alone and in combination with high concentrations of fatty acids [25]. The KGN cells, which are derived from a stage-3 diagnosed granulosa cell tumor removed from a 63-year-old Japanese woman in 1984, are believed to have originated from a dominant follicle at the antral or pre-antral stage. These cells bear functional FSH receptors and share other similarities with ovarian granulosa cells in vivo, including cAMP-inducible aromatase expression. Therefore, these cells represent a unique system for the characterization of signaling pathways involved in human folliculogenesis [26]. Our study is, to our knowledge, the first to use a high-throughput approach to profile the transcriptional responses of human granulosa cells to this particular context of metabolic syndrome. Using this specific in vitro model also allows the main signalling pathways to be dissected in response to these three main free fatty acids either alone or in combination with insulin. Therefore, this study should be regarded as a reference for further in-depth research on major canonical pathway as well as major hub genes that could be involved in the response to human granulosa cells to such metabolic challenges.

## Methods

### Human granulosa-like tumor cell line (KGN)

The granulosa cell line KGN, a steroidogenic human ovarian granulosa cell line, was purchased from the RIKEN Bioresource Centre (Tsukuba, Japan). This cell line maintained physiological characteristics of ovarian granulosa cells, including the expression of functional FSH receptors and steroidogenic activity such as estradiol production in response to FSH stimulation [26]. The cells were cultured in DMEM/F12 medium (Life technologies) supplemented with 10% foetal bovine serum (Corning), 100X Penicillin-Streptomycin (10,000 U/mL) (Life technologies) in an atmosphere of 5% CO_2_/95% O_2_ at 37 °C. According to the Human Research Ethics Committee of Laval University (CÉRUL), a research ethics board (REB) review was not required to work with the KGN cell line.

### NEFA treatments

The concentrations of non-esterified fatty acids (NEFA) in the follicular fluid of obese women are significantly correlated with BMI, with the most abundant being palmitic acid (PA, 16:0), stearic acid (SA, 18:0), and oleic acid (OA, 18:1) [15]. To mimic the physiological metabolic conditions found in obese women and to be able to characterize the transcriptomic profile of KGN cells exposed to metabolic challenges similar to obesity, three conditions were set up (Table 1). We first assessed the consequences of exposure of KGN cells to a combination of free fatty acids found in higher concentrations in obese women on their transcriptomic profile. We also assessed the transcriptomic profiles of KGN cells exposed to a high concentration of insulin alone and in combination with high concentrations of fatty acids in order to mimic the insulin resistance that is often observed with obesity (Table 1).

**Table 1:** Fatty acids and insulin concentrations for each experimental condition

Treatment	Culture media	Total FFAs
CTRL	-	-
INSULIN	100 ng/mL insulin	-
HIGH FAT (HF)	PA 75 μM + SA 25 μM + OA 100 μM	200 μM
HIGH FAT insulin (HFIns)	PA 75 μM + SA 25 μM + OA 100 μM *+*100 ng/mL insulin	200 μM

### Preparation of NEFA treatments

The DMEM medium was first supplemented with 1% low fatty acid bovine serum albumin (BSA) (ABFF-100G, MP Biomedicals, Auckland, NZ) in order to improve fatty acid solubility. The fatty acids, PA (product P0500), SA (product S-4751), and OA (product T7140), all purchased from Sigma-Aldrich (St. Louis, MO), were prepared in stock solutions in ethanol 100% (ETOH) at concentrations of 225 mM, 75 mM, and 300 mM, respectively. The fatty acid solutions were vortexed for 4 to 5 min before being added to the medium at working concentrations. The final medium mix was incubated in an atmosphere of 5% CO_2_/95% O_2_ at 37 °C for 24 h before being sonicated for 5 to 6 min in a small bath sonicator. Human insulin solution (Sigma-Aldrich product I9278) was added directly to the appropriate well at the time of treatment.

### Cell culture

The KGN cells were thawed on day 1, placed in a cell culture flask (75 cm^3^, Sigma-Aldrich), grown up to 80% confluence, and sub-cultured on day 4. On day 7, viable cells were seeded in a six-well plate at a density of 3×10^5^ per well in 3 ml of medium and grown for 72 h to obtain full confluence. For all experimental conditions, the medium was then replaced with DMEM/F12 containing antibiotics, 100 nmol L^-1^ of 4-androstene-3,17-dione (product A9630, Sigma-Aldrich, St. Louis, MO) and 1% of low fatty acid BSA. Plates were incubated for 24 h in a humidified incubator at 37 °C in 5% CO2:95% air after adding either ethanol (less than 0.1% of the culture volume) as a control or the fatty acid combination described above with or without the addition of human insulin. Control wells received the same volume of ethanol as the test groups.

### RNA purification and deep sequencing

Total RNA was isolated using the Norgen’s RNA/DNA Purification Kit (Cat. 48,700, Norgen Biotek Corporation, Canada) according to the manufacturer’s instructions. Total RNA integrity and concentration were assessed on a 2100-Bioanalyzer (Agilent Technologies, Palo Alto, CA) and the RNA Integrity Number (RIN) was 10 for all the samples. For each treatment, 270 ng of total RNA was used for mRNA isolation (90 ng from each biological replicate). As described in a previous study, mRNA was isolated from total RNA using the NEBNext Poly (A) mRNA Magnetic Isolation Module (E7490S; NEB) and was fragmented to a mean size of 200 nt. Fragments were reverse transcribed to generate double-stranded cDNA and converted to a paired-end library using the NEBNext Ultra RNA Library Prep kit for Illumina (E7530S; NEB) according to the manufacturer’s instructions and using Agencourt AMPure XP beads and NEBNext Mutliplex Oligos for Illumina (set1, E7335S; NEB) [27]. The libraries were sent to the Génome Québec Innovation Centre at McGill University (Montreal, Quebec, Canada) for quality control tests and then pooled at equimolar concentrations and sequenced on Illumina HiSeq4000 in paired-end mode with 100 base pair reads (PE100) to a depth between 53–69 million reads.

### Transcriptome assembly and expression level estimate from read counts

Ensembl (release 91) was used as the source of annotated genes and transcript isoforms. Using Trimmomatic [28], sequencing adapters were removed and base calls with a quality score below 30 were removed from the end of the reads [28]. Only reads with a minimum length of 32 nt were retained for further processing. The abundance of all transcripts described in release 91 of ENSEMBL cdna gene annotation was estimated by pseudoalignment using kallisto [29]. Differential expression of genes was then assessed using pairwise comparisons in edgeR [30]. Because we did not have replicates, dispersion within edgeR was evaluated by grouping samples for similar comparisons (first for control, then for high fat, high fat/insulin and insulin alone) and dropping the factor with the lowest impact, as suggested by the edgeR manual. The sequence data produced in this study were submitted to NCBI GEO under accession number GSE161914. As described in a previous study, normalized data were sorted for significance and then filtered according to the Transcript Support Level (TSL) used by the Ensembl gene annotation system [27]. This annotation system is a method to highlight the well supported and poorly supported transcript models for users. The method relies on the comparison of the mRNA and EST alignments to the GENCODE transcripts, and the transcripts are scored according to how well the alignment match over their full length. Differentially expressed genes selected for the present analysis were assigned to the evaluated annotations tsl1 which means that all splice junctions of the transcript were supported by at least one non-suspect mRNA.

## Results

### Differentially expressed genes in treatments

Using KGN cells and RNA-seq technology, we performed transcriptome analysis of three different treatments of fatty acids combination at high physiological concentration and high concentration of insulin in combination or alone. For this analysis INS treatment, HF treatment and HFIns treatment were all individually compared to the control group using pairwise comparisons. Following gene expression level analysis, 1,615 up- and down-regulated differentially expressed genes (DEGs) were mapped and used in the insulin treatment (INS) for subsequent functional analysis. Totals of 1,700 DEGs and 1,634 DEGs were mapped for the high fat (HF) and high fat-insulin (HFIns) treatments, respectively. Curated lists of the first 100 most significant upregulated and downregulated DEGs for each treatment are available in supplemental data (Supplemental Table S1-S6). To provide a global picture of the principal upstream signaling pathways and genomic mechanisms involved in the treatments, functional analysis was conducted using the Ingenuity Pathway Analysis (IPA) software which is a web-based software that allows the analysis and interpretation of data derived from omics experiments. Comparing our dataset to the Ingenuity Knowledge Base which organizes biological interactions and functional annotations, and attributes a probability of association between genes in the dataset and biological functions, we were able to extract the major biological functions as well as potential upstream regulators in our system.

### Major canonical pathways related to insulin and fatty acid signaling

The lists of DEGs from RNAseq data analysis of each treatment were separately uploaded into the IPA software and analyzed for the major biological functions that were the most significant to our dataset. A complete list of the significant canonical pathways affected by each treatment and identified by IPA is available in supplemental data (Supplemental table S7-S9). Table 2 presents several of the most significantly affected canonical pathways identified by IPA software for the Ins, HF and HFIns treatments (Table 2). The level of significance was measured by the ratio of the number of genes in our dataset that matched a given pathway over the total number of genes listed for this given pathway in the Ingenuity Knowledge Base. A p-value was also calculated with the Fisher’s exact test to assess the probability of association between the gene’s dataset and the targeted canonical pathway. The canonical pathways were also described with an activation z-score. The activation z-score is an overall score that is used to infer an activation state for a given biological function. By comparing the observed differential regulation of a gene (“up” or “down”) in the dataset to the known regulation direction associated with a specific canonical, the IPA software attributed a predicted activated (Z-score ≥ 2) or an inhibited (Z-score ≤ -2) score to biological functions of our system. Enriched canonical pathways analysis essentially show that INS treatment is predicted to affect genes related to the calcium signaling with a slight tendency toward inhibition (z-score = -0.426) as well as genes involved in the B cell receptor signaling (z-score = -1.095). Similarly, HF treatment is predicted to negatively affect several major signaling pathways such as the protein kinase A signaling (z-score = -1.342), the PI3k signaling in B lymphocytes (z-score = -1.400) and to a lesser degree the AMPK signaling (z-score = -0.626). Combination of both treatments principally affects antioxidant action of vitamin C, type I diabetes mellitus signaling and April mediated signaling but also genes predicted to have a positive impact on the NFAT signaling as demonstrated by the z-score (z-score = 2.121).

**Table 2:** Enriched canonical pathways of the differentially expressed genes using Ingenuity Pathway Analysis (IPA) software for insulin, high fat and high fat/insulin treatments

	-log (*p*-value)	Ratio	z-score
**Insulin**			
Calcium signaling	7.98	2.12E-01	-0.426
p53 signaling	5.00	2.16E-01	0.218
Signaling by Rho Family GTPases	4.72	1.63E-01	0.324
Superpathway of inositol phosphate compounds	4.62	1.67E-01	0.000
B cell receptor signaling	4.56	1.76E-01	-1.095
**High fat**			
Calcium signaling	9.77	2.37E-01	0.557
Amyloid process	5.52	3.20E-01	0.000
B cell receptor signaling	5.25	1.91E-01	-0.690
Role of NFAT in cardiac hypertrophy	4.62	1.76E-01	-0.822
GNRH signalling	4.60	1.91E-01	-1.633
Protein kinase A signalling	4.28	1.48E-01	-1.342
PI3k signaling in B lymphocytes	4.10	1.98E-01	-1.400
Type II diabetes mellitus signalling	3.17	1.71E-01	0.000
AMPK signaling	2.15	1.40E-01	-0.626
**High fat insulin**			
RANK signaling in osteoclasts	4.68	2.20E-01	-0.447
Antioxidant action of vitamin C	3.92	2.02E-01	-1.886
IL-6 signaling	3.86	1.88E-01	0.000
April mediated signaling	3.61	2.82E-01	-1.508
CD27 signaling in lymphocytes	3.58	2.50E-01	0.000
NF-κB signaling	3.42	1.63E-01	-0.186
B Cell activating factor signaling	3.40	2.68E-01	-1.265
Type I diabetes mellitus signaling	3.30	1.87E-01	-2.357
Role of NFAT in cardiac hypertrophy	3.29	1.53E-01	2.121
IL-4 signaling	3.18	1.98E-01	0.000

### Functional analysis

**Fig. 1:**
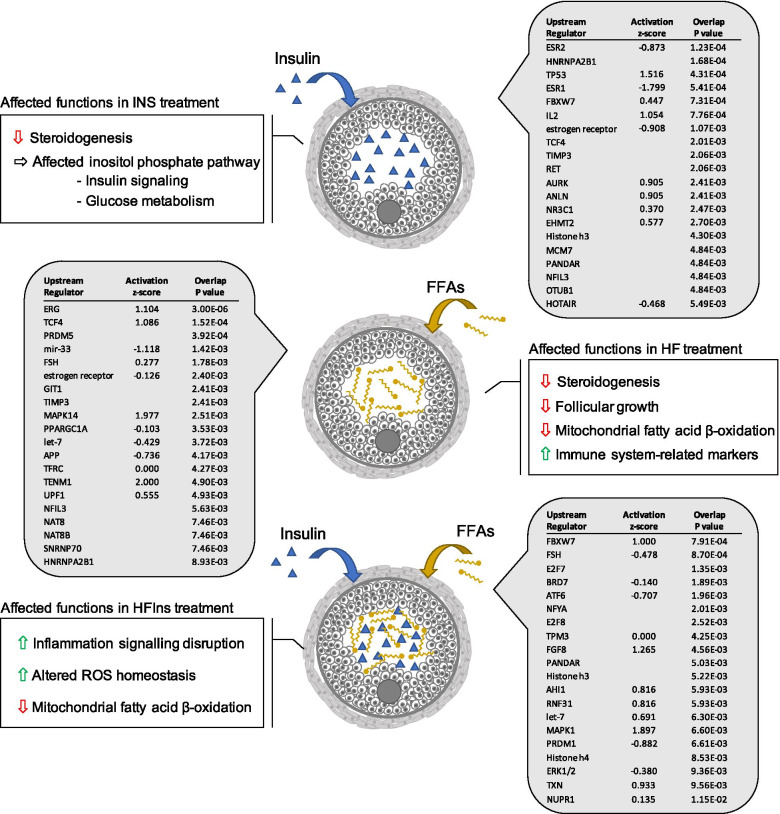
The most significant upstream regulators identified by IPA for each treatment (Insulin alone, free fatty acids (FFAs) alone or with added insulin) and major follicular functions affected according to canonical and upstream analysis

The IPA Upstream Regulator analytic tool was also used to identify the main upstream regulators driving the observed gene expression changes in each treatment. The top 20 transcriptional regulators for the INS, HF and HFIns treatments are shown in Fig. 1. The overlap p-value was calculated with the Fisher’s Exact Test and ranked the upstream regulators based on significant overlap between the gene in the dataset and the known gene targets of a given upstream regulator included in IPA’s database. As for the canonical pathways, the z-score inferred the activation of the transcriptional regulators by comparing the expression of genes in the dataset to what is expected from the literature. A complete list of all the significant upstream regulators for each treatment is available in supplemental data (Supplemental table S10-12). For further in-depth analysis of some canonical pathways, lists of downstream DEGs were uploaded to the PANTHER classification system for functional classification. Briefly, the PANTHER classification system employs the Gene Ontology (GO) and exploits a given list of genes to find functional classes and to provide curated associations of genes to biological pathways from the PANTHER Pathway resource [31].

For KGN cells exposed to insulin for 24 h, IPA revealed upstream regulators related to cell proliferation, estrogen regulation, fatty acid metabolism, and glucose homeostasis. Also, among the most significant upstreams identified by the treatment, post-transcriptional factors linked to the regulation of insulin secretion and signaling pathways in insulin-sensitive cells were highlighted by the software. For KGN cells exposed to insulin, IPA identified a cell proliferation-related factor, the yes associated protein 1 (YAP1), as a significant and activated (z-score = 2,438, *p*-value = 3,71E-02) upstream regulator; while a chemoattractant for macrophages, the colony-stimulating factor 1 (CSF1) (z-score = -2,433, *p*-value = 7,67E-03) was identified as an inhibited upstream regulating seven DEGs in our dataset (Supplemental Table 10). Among the significant upstream regulators identified, tumor protein P53 (TP53) (z-score = 1,516, *p*-value = 4,31E-04), as well as POU class 5 homeobox 1 (POU5F1) (z-score = 1,445, *p*-value = 1,04E-02) had a tendency toward activation. The peroxisome proliferator activated receptor gamma (PPARG) (z-score = -1,451, *p*-value = 3,79E-02) as well as the estrogen receptor 1 (ESR1) (z-score = -1,799, *p*-value = 5,41E-04) tended toward inhibition according to the software cut-offs. Among the top significant upstream regulators identified by the software, many were related to glucose homeostasis such as F-box and WD repeat domain containing 7 (FBXW7, also known as CDC4), nuclear receptor subfamily 3 group C member 1 (NR3C1), and nuclear factor interleukin 3 regulated (NFIL3) [32–34]. Others such as HOX transcript antisense RNA (HOTAIR) and euchromatic histone lysine methyltransferase 2 (EHMT2), which encodes for a ubiquitously expressed histone methyltransferase, were linked to the regulation of insulin secretion and signaling pathways in insulin-sensitive cells [35, 36].

Predicted regulator analysis of cells exposed to the high fat (HF) treatment suggested potential activation of several signaling pathways related to lipid metabolism, regulation of β-catenin signaling, and programmed cell death. Posttranscriptional factors known to play roles in fatty acid metabolism, insulin signaling, and granulosa cells programmed death were also identified in the upstream analysis for the HF treatment. The predicted regulator with the most significant p-value was the erythroblast transformation-specific transcription factor ERG (ERG) protein (*p*-value = 3,79E-02) encoded by a gene that was recently reported to be upregulated by androgen treatment, downregulated in atretic bovine medium follicles, and suggested as a potential target in polycystic ovary syndrome (PCOS) pathological changes in human granulosa cells [37, 38]. Among the top 10 most significant predicted upstream regulators, a conserved microRNA (miRNA) family, miR-33, which plays a role in fatty acid metabolism and insulin signaling [39] was identified (z-score = -1,118, *p*-value = 1,43E-03). Let-7, another miRNA family acting at the posttranscriptional level and involved in granulosa cell programmed death was also identified in HF treatment (z-score = -0,429, *p*-value = 3,72E-03). The PPARG coactivator 1 alpha (*PPARGC1A*) gene (z-score = -0,103, *p*-value = 3,53E-03) was identified as well and is another candidate gene involved in follicular atresia and in the regulation of genes involved in energy metabolism [40, 41]. A key effector of stress signals in cells, the mitogen-activated protein kinase 14 (MAPK14), was also identified by IPA as a predicted regulator with a strong tendency toward activation in the HF treatment (z-score = 1,977, *p*- value = 2,51E-03).

Finally, upstream regulator analysis of KGN cells exposed to the combined high fat and insulin (HFIns) treatment revealed predicted regulators related to cell cycle progression, glucose homeostasis, as well as steroidogenesis signaling and regulation of cellular oxidative status. Two factors potentially involved in glucose homeostasis, F-box and WD repeat domain containing 7 (FBXW7) and bromodomain-containing protein 7 (BRD7), were influenced by the HFIns treatment [34, 42]. FBXW7, the most significant upstream regulator in this treatment, was also among the top five most significant upstream for the insulin treatment. The miRNA let-7, which was identified in the HF treatment, was also influenced by the HFIns treatment. Other important factors involved in the cell cycle progression such as E2F transcription factor 7 (E2F7) and E2F transcription factor 8 (E2F8) were highlighted by the upstream analysis. The HFIns treatment is suggested to have affected FSH signaling as well as steroidogenesis regulation since several factors such as fibroblast growth factor 8 (FGF8), mitogen-activated protein kinase 1 (MAPK1), and the extracellular signal-regulated kinase 1/2 (ERK1/2), which are involved in estradiol signaling, were identified among the most significant predicted regulators. The thioredoxin (TXN) gene, a regulator of cellular redox status, was also highlighted as a significant predicted regulator in the HFIns treatment with a z-score of almost one (z-score = 0,933, *p*-value = 9,56E-03).

## Discussion

Obese women have decreased fertility, with decreased numbers and quality of oocytes and embryos. Although the biology and mechanisms are still poorly understood, there is rising evidence that metabolic disturbances in obese women affect reproductive functions [1, 2, 43, 44]. Among the metabolites found in follicular fluid and closely affecting follicular and oocyte development, free fatty acids were identified as key molecules contributing to the obesity-associated impaired oocyte developmental competence and metabolism [15]. Along with the increase in fatty acid concentrations, insulin levels were also increased in women afflicted with metabolic [15, 18, 45, 46]. The results presented here revealed the direct sensitivity of granulosa cells to the metabolic context of the female in a dominant-like follicular stage. Using a unique in vitro human cell model that allows the study of gene signaling in a more stable context when compared to human granulosa cells obtained from in vitro fertilization patients, we were able to characterize the major biological functions and pathways likely to be affected by an increase in free fatty acids and insulin in the surrounding cellular environment. Results revealed the granulosa cell response and the mechanisms triggered when facing a metabolic challenge such as an increase in fatty acid concentration which is one of the main consequences of obesity in women. The analysis of the transcriptomic response highlights numerous genes and pathways related to steroidogenesis, mitochondrial and energy metabolism as well as oxidative stress and inflammation-related hub genes and canonical pathways. Results also support the fact that a combination of high fat and high insulin may be worse than high fat alone, as not only the apoptotic signature increased but cytokines associated with inflammation and oxidative stress-related pathways were also higher in the combination treatment.

### Mitochondrial and energy metabolism

Transcriptomic analysis with the IPA software revealed that granulosa cell exposure to high concentrations of free fatty acids affected major canonical pathways related to mitochondrial function and energy metabolism such as calcium signaling, the phosphatidylinositol 3-kinases/protein kinase B (PI3K/AKT) signaling, and the 5’ AMP-activated protein kinase (AMPK) signaling. Calcium signaling was identified as the top disrupted canonical pathway in response to the HF treatment. In granulosa cells, calcium homeostasis was shown to be influenced by both FSH and LH, and to mediate mitochondrial biogenesis in differentiated human granulosa cells by regulating several factors involved in mitochondrial DNA (mtDNA) transcription and replication [47, 48]. Mainly studied in liver and in pancreatic tissues, calcium signaling disruption related to nutrient stress, or metabolic conditions such as obesity, was suggested to impair the endoplasmic reticulum (ER) and mitochondria integrity and functions as reviewed by Arruda et al., 2015. Saturated fatty acid incorporation in the ER membrane affected membrane fluidity and the function of Ca^2+^ linked receptors [49–54]. Intracellular Ca^2+^-dependent signaling molecules are involved in the regulation of cellular metabolism as many proteins and enzymes participate in fatty acid metabolism, tricarboxylic acid cycles, as well as apoptosis [55, 56]. Disruption of intracellular calcium homeostasis was directly related to the pathogenesis of insulin insensitivity in liver [57]. Tending towards inhibition in response to the insulin treatment, and activation in response to the HF treatment, such a Ca^2+^-related mechanism might be critical in the onset of impaired granulosa cell function resulting from metabolic challenges especially in type 2 diabetes (T2D) patients.

Along with the other canonical pathways affected by the HF treatment, the protein kinase A (PKA) and PI3K signaling were also identified as impaired with a tendency towards inhibition. Insulin action in human granulosa cells, including glucose uptake, was shown to be mediated via the insulin receptor substrate 1 (IRS1) protein and PI3K/AKT signaling pathways [25, 58, 59]. Accumulation of fatty acid metabolites such as dyacylglycerides (DAG) and ceramides was suggested to affect key factors of the PI3K signaling, to inhibit insulin signaling, and to induce endoplasmic reticulum (ER) and oxidative stress [60, 61]. In our results, the negative effect on the PI3K signaling pathway may indicate that fatty acids are involved in the induction of insulin resistance by interfering with this pathway in human granulosa cells as observed in a previous study on human and mouse granulosa cells [22]. Moreover, disruption of PKA and AKT signaling pathways affected many granulosa cell key functions such as steroidogenesis and growth and differentiation [62]. The actions of both gonadotrophins, FSH and LH, are mediated via PKA and AKT signaling. PKA is a master kinase for granulosa cell differentiation and proliferation while AKT acts as a permissive pathway for PKA signaling and regulates cell survival [27, 63–65]. Consistent with our results, obesity in women was associated with lower levels of estradiol [46, 66]. In a recent study, Xu et al., 2019 also showed decreased estradiol (E2) production in women with elevated BMI along with a reduced response to FSH stimulation exacerbated in the presence of high insulin concentrations [24]. Furthermore, AKT targets many metabolism-related factors such as FOXO1 and peroxisome proliferator-activated receptor coactivator 1α (PGC1α) which regulate gene expression to increase gluconeogenesis and fatty acid oxidation [67]. The PPARG coactivator 1 alpha (*PPARGC1A*) gene (z-score = -0,103, p-value = 3,53 E-03) encoding for the PGC1α protein was identified in our study and was previously suggested to be involved in follicular atresia as well as steroidogenesis regulation and progesterone production in granulosa cells [41, 68, 69]. Slightly inhibited as a predicted regulator in the HF treatment, this gene was significantly upregulated in the insulin treatment and inhibited in the HFIns treatment. The transcriptional coactivator protein PGC-1α, which is stimulated through calcium signaling, insulin, and p38 MAPK action, was described as a master regulator of mitochondrial biogenesis and function, and is thought to act as a key factor regulating multiple metabolic pathways such as gluconeogenesis and fatty acid synthesis and oxidation, or glycolysis [70]. As reviewed by Cheng et al. 2018, PGC-1α binds to target genes that influence mitochondrial metabolism and impact fatty acid utilization and transport. In our HF treatment, IPA analysis suggested *PPARGC1A* regulation of Carnitine Palmitoyltransferase 1B (CPT1B), glycerol kinase (GK), and cluster of differentiation 36 (CD36), all DEGs related to FA uptake, triglyceride metabolism, and fatty acid oxidation (FAO) [71–74]. Closely related to *PPARGC1A* action, another energy sensor and energy homeostasis regulator sensitive to the AMP:ATP ratio was identified by our study as being negatively affected in granulosa cells exposed to fatty acids [75]. The AMP-activated protein kinase (AMPK) is a well-known regulator of enzymes involved in the synthesis of fatty acids, cholesterol, and glycogen [76]. It is involved in the pathogenesis of insulin-resistant states and is targeted by metformin, an insulin sensitizer, for the treatment of type 2 diabetes. The alteration of AMPK activity in stress conditions such as obesity can lead to dysregulated metabolism and insulin resistance [77–79]. Consistent with our results, and as reviewed by Lyons and Roche, 2018, obesity was associated with reduced AMPK activation and reduced fatty acid oxidation [80, 81]. Several studies investigated the role of AMPK in granulosa cells and the results were summarized by Bertoldo et al., 2014 [82–85]. In bovine and human granulosa cells, AMPK activation by metformin reduced estradiol and progesterone production as well as steroidogenesis-related proteins [85, 86]. The AMPK enzyme regulates a wide array of physiological and cellular events and seems to have diverse functions in granulosa cells which evolve during folliculogenesis. Our results reinforced its role as a key molecule involved in human granulosa cell sensitivity to the metabolic context [82].

In agreement with the literature and the results mentioned above, the mitogen-activated protein kinase 14 (MAPK14), also called p38-α, was also identified as a significant predicted regulator with a strong tendency toward activation in the HF treatment. The p38 protein is a key effector activated by stress signals and oxidative stress and is involved in inflammation and apoptosis processes. In granulosa cells, along with its suggested role in the pro-apoptosis/pro-survival balance, p38 was also reported to be involved in steroidogenesis regulation by stimulating estradiol production and reducing progesterone production [87–90]. The p38 protein was also directly activated by saturated fatty acids in human pancreatic β-cells and was linked to energy metabolism in several tissues, regulating gluconeogenesis among other processes [87, 91]. This factor plays a key role in FFA-induced transcription of gluconeogenic genes including PPARGC1A, and was highlighted as a key molecule in the response of bovine granulosa cells to the mother nutrition and metabolic stress status thereby influencing fertility outcomes [92–94]. Among the downstream effectors of MAPK14 affected by treatment was the vitamin D receptor (VDR) which was upregulated (DEG) in the insulin and HF treatments but downregulated in the combination treatment (HFIns). Although the pathogenetic mechanisms linking obesity, vitamin D, and fertility are still not understood, vitamin D deficiency was previously associated with female infertility [95]. Additionally, the VDR was suggested to be involved in the regulation of genes involved in glucose and lipid metabolism [96]. Accumulating evidence from human studies, reviewed by Anagnostis et al. in 2013, suggested a key role of vitamin D deficiency in metabolic disturbances including insulin resistance in polycystic ovary syndrome (PCOS) in obese women [97, 98]. The expression of the VDR gene was downregulated in PCOS and in obese women when compared to non-PCOS and non-obese women [99]. Besides playing a role in energy metabolism, the VDR is also involved in steroidogenic functions in human ovarian and granulosa cells [100–102].

Taken together, the results showed that when exposed to high concentrations of fatty acids, granulosa cells exhibited a certain sensitivity through modulations of numerous genes and pathways related to mitochondrial biofunctions, energy metabolism, and steroidogenesis. Analysis of major canonical pathways and upstream regulators suggested an impaired steroidogenesis, a predicted reduced response to gonadotrophin and insulin levels, and a dysregulated fatty acid-related metabolism mostly involving the PI3K/AKT signaling. Overall, although the results of this study provided a limited insight into the onset response of human granulosa cells, this study allowed us to identify potential new pathways and targets worth considering for further research.

### Inflammation and oxidative stress

As a consequence of ER and mitochondrial stress, FFA were suggested to induce a lipotoxic response and to increase levels of reactive oxygen species (ROS) [103]. Persistent cell damage and chronic low-grade inflammation are the hallmarks of reproductive tissues exposed to FFA, and follicles from obese women presented inflammation characteristics [18, 104]. Consistently, results from this study identified the nuclear factor of activated T-cells (NFAT) signaling pathway with a tendency toward inhibition among the top 10 canonical pathways affected by the HF treatment. The NFAT family members, studied mostly in T and B cells, participated in transcriptional induction of various immune response genes as well as cytokine gene expression [105, 106]. Thought to be key transcription factors for immune cell activation and differentiation, NFAT family members were also suggested to be involved in adipocyte differentiation, modulation of adipokine gene expression, and to participate in glucose and insulin homeostasis [105, 107–109]. Although NFATC1 was upregulated following stimulation of porcine granulosa cells with a phospholipase C (PLC) activator [110], the actions mediated by the NFAT family in granulosa cells still remain poorly understood. Functional classification of the 38 downstream effector genes of the NFAT canonical pathway using PANTHER Classification systems showed that the differentially expressed genes were, among others, related to inflammation mediated by chemokine and cytokine signaling pathways, and to the 5HT2 type receptor or serotonin receptor (5-HT2A) signaling pathway which is known to stimulate an increase in intracellular Ca^2+^ levels via the PLC/IP3/DAG pathway [111]. This canonical pathway tended to be inhibited in the HF and insulin treatments, but activated in the HFIns treatment (z-score = 2.212) suggesting that high fat in combination with a high insulin concentration could be a key signal for the onset of inflammatory and immune processes in human granulosa cells [112–114]. As reviewed by Duffy et al., (2019), women with PCOS, which is often associated with obesity and high insulin levels, had altered inflammatory transcriptome in granulosa cells as some inflammatory genes were over-expressed, along with genes responsible for leukocyte migration [113, 115–118]. Obesity in combination with PCOS seemed to enhance inflammatory gene expression as overweight PCOS women exhibited the most differences in gene expression profiles among the contrasts studied [115]. Consistent with this understanding of the results, analysis of the HFIns treatment highlighted interkeulin 6 (IL-6) among the top 3 canonical pathways and interleukin 4 (IL-4) as being involved in granulosa cell response to such metabolic challenge. Interleukin 4 is an anti-inflammatory cytokine and Il-6 is a proinflammatory pleiotropic cytokine secreted by granulosa cells [119, 120]. Modulated by known pro-inflammatory cytokines such as interleukin-1a (IL1A), IL6 was suggested to contribute to the ovulatory process and to be involved in bidirectional communication between the ovary and cellular components of the immune system [121]. It also increased in ovaries from obese women and mice and may participate in the chronic low-grade inflammation associated with the PCOS pathogenesis [115, 122–124].

Regarding the oxidative stress that is often increased in obesity and associated metabolic diseases, the thioredoxin (TXN) gene, which encodes the TXR protein a ubiquitously expressed small protein playing a key role in regulating the cellular redox status, was highlighted as a predicted regulator with a tendency towards activation in the HFIns treatment [125]. Long-chain saturated fatty acids were associated with the generation of proapoptotic molecules and the accumulation of intracellular ROS thereby inducing the dysregulation of mitochondrial function and resulting in lipotoxicity [103]. Maternal obesity during the periconceptional period disrupted mitochondrial homeostasis and increased the levels of ROS in mouse oocytes and zygotes [126]. Moreover, obese women had higher follicular fluid oxidative stress markers [127]. Thioredoxin, a component of the TRX/TRXR system induced by oxidative stress-generating stimuli and involved in antioxidant defense mechanisms, was present throughout follicular development in women and was also identified in follicular fluid [125, 128, 129]. The predicted activation of the thioredoxin pathway following treatment with both insulin and high fat could suggest an enhanced oxidative environment compared to high fat exposure alone or insulin alone. In line with this, the antioxidant action of vitamin C was identified as the third most significant canonical pathway in the HFIns treatment. Vitamin C (L-ascorbic acid) protects against oxidative stress and cellular damage induced by ROS [130]. In mice, vitamin C exerted beneficial effects against ovarian aging due in part to its antioxidant capacity [131]. Moreover, previous studies reported that this hormonally regulated vitamin played a key role in preventing atresia and apoptosis in granulosa cells and dominant follicles [132, 133]. The tendency toward inhibition of this pathway could suggest a disturbed equilibrium of pro-oxidant and antioxidant molecules in human granulosa cells exposed to a combination of high insulin and high fat.

Finally, a noteworthy effect of the high concentrations of FFA and insulin (HFIns) was the strong involvement of the tumor necrosis factor (TNF) superfamily and the nuclear factor-kappa B (NF-κB) family in the granulosa cell response as almost all the top canonicals were directly or indirectly related to those two signaling pathways. Among the top 10 most significant pathways affected by this treatment was the RANK signaling in Osteoblasts. The RANK protein, encoded by TNFRSF11A, also known as receptor activator of NF-κB (RANK), is a key molecule that regulates the differentiation and function of osteoclasts. As reviewed by Park et al. (2007), RANK signaling can lead to the activation of several mitogen-activated protein kinases (MAPKs) as well as the transcription factor nuclear factor-κB (NF-κB) [134]. Among the DEGs suggested as being part of this canonical pathway, many MAP kinases were included as well as NF-κB signaling pathway-related genes (MAPK14, NFKB Inhibitor Delta (NFKBID), inhibitor of nuclear factor kappa B kinase subunit beta (IKBKB), inhibitor of nuclear factor kappa B kinase regulatory subunit gamma (IKBKG), nuclear factor NF-kappa-B p65 subunit (RELA), etc...). The nuclear factor - kappa B (NF-κB) family is a group of transcription factors involved in many well-recognized cell functions including regulation of inflammatory response, cell survival/cell apoptosis, maturation of the immune system, and cell differentiation [135–137]. In bovine and porcine granulosa cells, NF-κB activity was linked to estradiol-mediated signaling pathway, progesterone production, as well as GC survival/apoptosis, with some discrepancies regarding its role in apoptosis among the different species [138–143]. Reinforcing the idea of a strong involvement of this pathway in response to the HFIns treatment, canonical analysis highlighted a treatment-related disruption of the APRIL signaling pathway, also known as TNFS13A, as well as the CD27 signaling pathway, also known as the tumor necrosis factor receptor superfamily 7 (TNFRSF7). Besides being regulated by RANK signaling, NF-κB family members are also regulated by many other cytokines of the TNF superfamily that stimulate various cell type- and pathway-specific responses [144]. The APRIL signaling, which was suggested to mediate a pro-survival response in cells and to act through the activation of the NF-κB pathway tended to be inhibited by the fatty acid and insulin combined treatment [145–148]. The CD27 protein, which is also related to the activation of the NF-κB signaling, was likewise identified as a top pathway due to its relation to the NF-κB family [149]. All connected together, inflammation-related factors such as TNFα, IL6, NOS2, and the transcription factor p65, also known as nuclear factor NF-kappa-B p65 subunit (RELA), were all increased in the ovarian tissue of obese mice [123, 150]. Interestingly, factors like NOS2 and RELA were inhibited in the HFIns treatment; and although the canonical pathway for NF-κB was neither activated nor inhibited in the HFIns treatment, our results still suggest that this pathway is influenced by treatment and could therefore be involved in the mechanisms through which human granulosa cells respond to metabolic challenges. Globally, when exposed to metabolic challenges such as high concentrations of fat and insulin, human granulosa cells seemed to activate a gene expression program linked to the immune response and to inflammatory processes. Although after only 24 h of exposition, the whole mechanism might not be fully engaged yet, the results demonstrated a certain sensitivity of the different signaling pathways previously mentioned. In agreement with the specific genomic signatures found in this study, inflammation and oxidative stress are two processes tightly linked with one another and this interdependence is a known contributing factor to the development of metabolic diseases.

Overall, this study provided, for the first time, a more comprehensive profile of gene expression in human granulosa cells exposed to metabolic challenge contexts of high-fat and/or high insulin levels. The results of our study suggested that human granulosa cells are sensitive to specific FFAs and to insulin in their environment. When exposed to such challenges, important changes and gene profile remodeling occur in relation to the energy and mitochondrial metabolism, homeostasis, as well as to key functions such as steroidogenesis for which the signaling pathways were markedly affected by our high fat treatment. Moreover, when high insulin levels were added to high fat concentrations, increased oxidative stress and inflammation related reactions were observed suggesting that the combination of both conditions might affect further follicular development in a more critical way. Even though we are aware that in vitro and in vivo contexts differ, as many other adipokines and molecules can be found in follicular fluid and are surely affecting human granulosa cells responses, this study still provided a great overall picture of the mechanisms taking part in this response. Cultured KGN cells have been found to bear functional FSH receptors and to be unresponsive to hCG stimulation suggesting a developmental stage close to antral/undifferentiated follicle [26]. Despite the fact that KGN cells do not differentiate in a typical pre-ovulatory stage which limits the developmental window under investigation, they represent a unique model for the study of antral/dominant follicular phase. Because of the own nature of KGN cells which are derived from a stage-3 diagnosed granulosa cell tumor further research and validation in native granulosa cells for each pathway put forward by our analysis will be needed [26, 151]. Nevertheless, with all the conserved characteristics of native granulosa cell including cAMP-inducible aromatase expression, it is believed that KGN cell line is a readily accessible system for characterizing signalling pathways in human folliculogenesis using mechanistic experiments [26]. Finally, even though high-throughput sequencing methods and software such as IPA are powerful tools for building gene correlation networks, biological interpretation of the massive amount of data generated remains a challenge. This study demonstrated that human granulosa cells can adjust to the maternal metabolism; and although this overall picture might not be exhaustive, this is a first step in the unravelling of the mechanisms involved.

## Supplementary Information

**Additional file 1: Supplemental Table S1.** List of the first 100 upregulated DEGs in High fat + insulin (HFIns) treatment. **Supplemental Table S2.** List of the first 100 downregulated DEGs in High fat + insulin (HFIns) treatment. **Supplemental Table S3.** List of the first 100 upregulated DEGs in High fat (HF) treatment. **Supplemental Table S4.** List of the first 100 downregulated DEGs in High fat (HF) treatment. **Supplemental Table S5.** List of the first 100 upregulated DEGs in Insulin (INS) treatment. **Supplemental Table S6.** List of the first 100 downregulated DEGs in Insulin (INS) treatment. **Supplemental Table S7.** Complete list of significant (*p*-value ≤ 0,05) enriched canonical pathways of the differentially expressed genes using Ingenuity Pathway Analysis (IPA) software for insulin (INS) treatment. **Supplemental Table S8.** Complete list of significant (*p*-value ≤ 0,05) enriched canonical pathways of the differentially expressed genes using Ingenuity Pathway Analysis (IPA) software for high fat (HF) treatment. **Supplemental Table S9.** Complete list of significant (*p*-value ≤ 0,05) enriched canonical pathways of the differentially expressed genes using Ingenuity Pathway Analysis (IPA) software for high fat + insulin (HFIns) treatment. **Supplemental Table S10.** List of most significant upstream regulators in Insulin (INS) treatment (*p*-value of overlap ≤ 0,05). **Supplemental Table S11.** List of most significant upstream regulators in High fat (HF) treatment (*p*-value of overlap ≤ 0,05). **Supplemental Table S12.** List of most significant upstream regulators in High fat + insulin (HFIns) treatment (*p*-value of overlap ≤ 0,05).

## Data Availability

The data discussed in this publication have been deposited in NCBI’s Gene Expression Omnibus and are accessible through GEO Series accession number GSE161914.
