# Supplementary material for: Gene cascade analysis in human granulosa tumor cells (KGN) following exposure to high levels of free fatty acids and insulin
Source: J Ovarian Res. 2021 Dec 20;14:178. doi: 10.1186/s13048-021-00934-6 (PMC8690403; doi:10.1186/s13048-021-00934-6)
Supplement: Supplementary file 1 — Additional file 1: Supplemental Table S1. List of the first 100 upregulated DEGs in High fat + insulin (HFIns) treatment. Supplemental Table S2. List of the first 100 downregulated DEGs in High fat + insulin (HFIns) treatment. Supplemental Table S3. List of the first 100 upregulated DEGs in High fat (HF) treatment. Supplemental Table S4. List of the first 100 downregulated DEGs in High fat (HF) treatment. Supplemental Table S5. List of the first 100 upregulated DEGs in Insulin (INS) treatment. Supplemental Table S6. List of the first 100 downregulated DEGs in Insulin (INS) treatment. Supplemental Table S7. Complete list of significant (p-value ≤ 0,05) enriched canonical pathways of the differentially expressed genes using Ingenuity Pathway Analysis (IPA) software for insulin (INS) treatment. Supplemental Table S8. Complete list of significant (p-value ≤ 0,05) enriched canonical pathways of the differentially expressed genes using Ingenuity Pathway Analysis (IPA) software for high fat (HF) treatment. Supplemental Table S9. Complete list of significant (p-value ≤ 0,05) enriched canonical pathways of the differentially expressed genes using Ingenuity Pathway Analysis (IPA) software for high fat + insulin (HFIns) treatment. Supplemental Table S10. List of most significant upstream regulators in Insulin (INS) treatment (p-value of overlap ≤ 0,05). Supplemental Table S11. List of most significant upstream regulators in High fat (HF) treatment (p-value of overlap ≤ 0,05). Supplemental Table S12. List of most significant upstream regulators in High fat + insulin (HFIns) treatment (p-value of overlap ≤ 0,05). [file 13048_2021_934_MOESM1_ESM.docx]

**Supplemental Data**

**Supplemental Table S1. List of the first 100 upregulated DEGs in High fat + insulin (HFIns) treatment.**

| gene name | target Id | transcript  biotype | transcript  tsl | H-FAT-Ins.  log FC | H-FAT-Ins.  PValue |
| --- | --- | --- | --- | --- | --- |
| RAPGEF1 | ENST00000372190,7 | protein_coding | tsl1 | 12,211 | 1,97E-09 |
| DDHD1 | ENST00000612692,4 | protein_coding | tsl1 | 11,645 | 9,06E-09 |
| MCRS1 | ENST00000546244,5 | protein_coding | tsl1 | 11,154 | 3,34E-08 |
| MGRN1 | ENST00000415496,5 | protein_coding | tsl1 | 11,129 | 3,47E-08 |
| HDAC6 | ENST00000376619,6 | protein_coding | tsl1 | 11,111 | 3,73E-08 |
| NGLY1 | ENST00000308710,9 | protein_coding | tsl1 | 10,946 | 5,80E-08 |
| BTN3A2 | ENST00000377708,6 | protein_coding | tsl1 | 10,723 | 1,05E-07 |
| TAF15 | ENST00000631482,1 | protein_coding | tsl1 | 10,719 | 1,05E-07 |
| TAF15 | ENST00000604841,5 | protein_coding | tsl1 | 10,719 | 1,05E-07 |
| GOLGA8A | ENST00000432566,6 | protein_coding | tsl1 | 10,640 | 1,30E-07 |
| NFIB | ENST00000543693,5 | protein_coding | tsl1 | 10,514 | 1,82E-07 |
| PUS3 | ENST00000227474,7 | protein_coding | tsl1 | 10,497 | 1,80E-07 |
| POLA1 | ENST00000379059,7 | protein_coding | tsl1 | 10,416 | 1,61E-07 |
| ZBTB22 | ENST00000418724,1 | protein_coding | tsl1 | 10,415 | 2,36E-07 |
| CTTN | ENST00000376561,7 | protein_coding | tsl1 | 10,255 | 3,59E-07 |
| ZMIZ1 | ENST00000611351,1 | protein_coding | tsl1 | 10,246 | 3,68E-07 |
| SMPD3 | ENST00000563226,1 | protein_coding | tsl1 | 10,203 | 4,11E-07 |
| VEGFA | ENST00000520948,5 | protein_coding | tsl1 | 10,149 | 4,74E-07 |
| LMNB1 | ENST00000395354,1 | protein_coding | tsl1 | 10,115 | 3,12E-07 |
| PTPA | ENST00000347048,8 | protein_coding | tsl1 | 10,088 | 5,41E-07 |
| CRNKL1 | ENST00000377327,8 | protein_coding | tsl1 | 10,027 | 6,04E-07 |
| BACH2 | ENST00000257749,8 | protein_coding | tsl1 | 9,989 | 7,21E-07 |
| BAZ2A | ENST00000379441,7 | protein_coding | tsl1 | 9,989 | 7,23E-07 |
| MYH7B | ENST00000262873,11 | protein_coding | tsl1 | 9,986 | 7,27E-07 |
| FILIP1L | ENST00000495625,2 | protein_coding | tsl1 | 9,924 | 8,55E-07 |
| CEP128 | ENST00000281129,7 | protein_coding | tsl1 | 9,908 | 8,90E-07 |
| SEC24D | ENST00000511481,5 | protein_coding | tsl1 | 9,865 | 9,98E-07 |
| RC3H1 | ENST00000258349,8 | protein_coding | tsl1 | 9,787 | 3,95E-07 |
| RPS6KB1 | ENST00000406116,7 | protein_coding | tsl1 | 9,782 | 1,24E-06 |
| ZNF566 | ENST00000493391,5 | protein_coding | tsl1 | 9,778 | 1,24E-06 |
| MIER3 | ENST00000381226,7 | protein_coding | tsl1 | 9,776 | 1,26E-06 |
| BIN1 | ENST00000348750,8 | protein_coding | tsl1 | 9,772 | 1,27E-06 |
| PHRF1 | ENST00000416188,2 | protein_coding | tsl1 | 9,769 | 6,78E-07 |
| POLE2 | ENST00000554396,5 | protein_coding | tsl1 | 9,759 | 1,31E-06 |
| CTAGE5 | ENST00000280083,7 | protein_coding | tsl1 | 9,685 | 1,59E-06 |
| PLEKHG3 | ENST00000484731,2 | protein_coding | tsl1 | 9,685 | 1,59E-06 |
| NEDD1 | ENST00000557644,5 | protein_coding | tsl1 | 9,676 | 1,63E-06 |
| CEP162 | ENST00000257766,8 | protein_coding | tsl1 | 9,639 | 1,80E-06 |
| PHRF1 | ENST00000633631,1 | protein_coding | tsl1 | 9,637 | 8,39E-07 |
| SLC23A2 | ENST00000379333,5 | protein_coding | tsl1 | 9,600 | 1,99E-06 |
| PRPF38B | ENST00000370021,1 | protein_coding | tsl1 | 9,579 | 2,10E-06 |
| PIK3C2B | ENST00000424712,6 | protein_coding | tsl1 | 9,529 | 2,39E-06 |
| ERMN | ENST00000410096,5 | protein_coding | tsl1 | 9,521 | 2,44E-06 |
| OSBPL3 | ENST00000396429,5 | protein_coding | tsl1 | 9,502 | 1,30E-06 |
| ARID4A | ENST00000417477,2 | protein_coding | tsl1 | 9,493 | 2,62E-06 |
| MTRR | ENST00000264668,6 | protein_coding | tsl1 | 9,489 | 2,65E-06 |
| PCDHA11 | ENST00000398640,6 | protein_coding | tsl1 | 9,444 | 2,33E-06 |
| ENDOV | ENST00000517795,5 | protein_coding | tsl1 | 9,394 | 3,39E-06 |
| LATS1 | ENST00000253339,9 | protein_coding | tsl1 | 9,385 | 3,47E-06 |
| DIAPH2 | ENST00000373049,8 | protein_coding | tsl1 | 9,381 | 3,50E-06 |
| HSPA8 | ENST00000453788,6 | protein_coding | tsl1 | 9,343 | 3,86E-06 |
| ZFYVE1 | ENST00000318876,9 | protein_coding | tsl1 | 9,340 | 3,89E-06 |
| SOCS4 | ENST00000339298,2 | protein_coding | tsl1 | 9,332 | 3,98E-06 |
| NR2C1 | ENST00000393101,7 | protein_coding | tsl1 | 9,272 | 4,64E-06 |
| P3H2 | ENST00000427335,6 | protein_coding | tsl1 | 9,262 | 4,75E-06 |
| DZIP1 | ENST00000361396,6 | protein_coding | tsl1 | 9,262 | 4,76E-06 |
| LYPLA1 | ENST00000343231,10 | protein_coding | tsl1 | 9,254 | 4,86E-06 |
| ZNF30 | ENST00000303586,11 | protein_coding | tsl1 | 9,235 | 5,10E-06 |
| KIF14 | ENST00000614960,4 | protein_coding | tsl1 | 9,218 | 5,33E-06 |
| MLH1 | ENST00000458205,6 | protein_coding | tsl1 | 9,206 | 5,49E-06 |
| NELFE | ENST00000375429,7 | protein_coding | tsl1 | 9,204 | 5,53E-06 |
| CRYZ | ENST00000417775,5 | protein_coding | tsl1 | 9,201 | 5,57E-06 |
| CD276 | ENST00000564751,5 | protein_coding | tsl1 | 9,117 | 3,17E-06 |
| ABCF1 | ENST00000376545,7 | protein_coding | tsl1 | 9,028 | 1,40E-06 |
| CDC42BPA | ENST00000366764,6 | protein_coding | tsl1 | 8,994 | 9,44E-06 |
| ZNF419 | ENST00000221735,11 | protein_coding | tsl1 | 8,990 | 9,53E-06 |
| PAM | ENST00000304400,11 | protein_coding | tsl1 | 8,917 | 1,15E-05 |
| HEXIM2 | ENST00000591576,5 | protein_coding | tsl1 | 8,912 | 1,16E-05 |
| CDC25A | ENST00000351231,7 | protein_coding | tsl1 | 8,902 | 1,19E-05 |
| VPS36 | ENST00000611132,4 | protein_coding | tsl1 | 8,879 | 1,26E-05 |
| RERG | ENST00000538313,5 | protein_coding | tsl1 | 8,862 | 1,32E-05 |
| OCLN | ENST00000355237,6 | protein_coding | tsl1 | 8,859 | 7,13E-06 |
| SGK3 | ENST00000396596,2 | protein_coding | tsl1 | 8,837 | 1,39E-05 |
| STX5 | ENST00000377897,8 | protein_coding | tsl1 | 8,823 | 1,45E-05 |
| LGR5 | ENST00000536515,5 | protein_coding | tsl1 | 8,810 | 1,51E-05 |
| RGS3 | ENST00000613049,4 | protein_coding | tsl1 | 8,804 | 1,53E-05 |
| AMACR | ENST00000512079,5 | protein_coding | tsl1 | 8,799 | 1,55E-05 |
| DGKD | ENST00000409813,7 | protein_coding | tsl1 | 8,781 | 1,62E-05 |
| TBRG4 | ENST00000361278,7 | protein_coding | tsl1 | 8,760 | 1,70E-05 |
| ANO6 | ENST00000441606,2 | protein_coding | tsl1 | 8,736 | 1,81E-05 |
| PALM2 | ENST00000314527,8 | protein_coding | tsl1 | 8,736 | 1,81E-05 |
| TCOF1 | ENST00000323668,11 | protein_coding | tsl1 | 8,734 | 1,82E-05 |
| ACTR2 | ENST00000377982,8 | protein_coding | tsl1 | 8,718 | 1,90E-05 |
| CYP2U1 | ENST00000508453,1 | protein_coding | tsl1 | 8,695 | 2,01E-05 |
| CD46 | ENST00000322875,8 | protein_coding | tsl1 | 8,670 | 2,14E-05 |
| C1QTNF3 | ENST00000231338,7 | protein_coding | tsl1 | 8,664 | 2,17E-05 |
| TRAK1 | ENST00000327628,9 | protein_coding | tsl1 | 8,630 | 2,36E-05 |
| DDAH2 | ENST00000454138,6 | protein_coding | tsl1 | 8,626 | 6,88E-06 |
| DDAH2 | ENST00000434464,6 | protein_coding | tsl1 | 8,626 | 6,88E-06 |
| DDAH2 | ENST00000447101,6 | protein_coding | tsl1 | 8,626 | 6,88E-06 |
| DDAH2 | ENST00000437889,6 | protein_coding | tsl1 | 8,626 | 6,88E-06 |
| VEPH1 | ENST00000392833,6 | protein_coding | tsl1 | 8,625 | 2,11E-05 |
| UBE2V1 | ENST00000415862,6 | protein_coding | tsl1 | 8,614 | 2,46E-05 |
| STAG1 | ENST00000236698,9 | protein_coding | tsl1 | 8,602 | 2,53E-05 |
| GALC | ENST00000622264,4 | protein_coding | tsl1 | 8,567 | 2,77E-05 |
| WDR44 | ENST00000371825,7 | protein_coding | tsl1 | 8,555 | 2,85E-05 |
| TMBIM1 | ENST00000396809,6 | protein_coding | tsl1 | 8,530 | 3,00E-05 |
| PTPA | ENST00000452489,6 | protein_coding | tsl1 | 8,529 | 3,02E-05 |
| C1orf112 | ENST00000413811,3 | protein_coding | tsl1 | 8,525 | 3,07E-05 |
|  |  |  |  |  |  |

**Supplemental Table S2. List of the first 100 downregulated DEGs in High fat + insulin (HFIns) treatment.**

| gene name | TARGET ID | transcript  biotype | | transcript  tsl | | | H-FAT-Ins.  log FC | H-FAT-Ins.  PValue | |
| --- | --- | --- | --- | --- | --- | --- | --- | --- | --- |
| IL34 | ENST00000288098,6 | | protein_coding | | tsl1 | -8,614 | | 2,96E-05 |  |
| ENO3 | ENST00000518175,1 | | protein_coding | | tsl1 | -8,630 | | 2,84E-05 |  |
| MTMR3 | ENST00000351488,7 | | protein_coding | | tsl1 | -8,642 | | 2,76E-05 |  |
| C1S | ENST00000402681,7 | | protein_coding | | tsl1 | -8,652 | | 2,70E-05 |  |
| TAP1 | ENST00000439781,2 | | protein_coding | | tsl1 | -8,675 | | 2,54E-05 |  |
| FOXJ3 | ENST00000445886,5 | | protein_coding | | tsl1 | -8,679 | | 2,52E-05 |  |
| SLC19A1 | ENST00000380010,8 | | protein_coding | | tsl1 | -8,681 | | 2,51E-05 |  |
| CCSAP | ENST00000284617,6 | | protein_coding | | tsl1 | -8,694 | | 2,43E-05 |  |
| C3orf18 | ENST00000449241,5 | | protein_coding | | tsl1 | -8,705 | | 2,36E-05 |  |
| PSMC3 | ENST00000298852,7 | | protein_coding | | tsl1 | -8,714 | | 7,71E-07 |  |
| CEP152 | ENST00000399334,7 | | protein_coding | | tsl1 | -8,737 | | 1,52E-05 |  |
| SEMA3B | ENST00000611067,4 | | protein_coding | | tsl1 | -8,739 | | 2,17E-05 |  |
| ECT2 | ENST00000417960,5 | | protein_coding | | tsl1 | -8,774 | | 1,99E-05 |  |
| SMN1 | ENST00000628353,2 | | protein_coding | | tsl1 | -8,785 | | 1,94E-05 |  |
| SLC22A17 | ENST00000637426,1 | | protein_coding | | tsl1 | -8,791 | | 1,91E-05 |  |
| PPP1R18 | ENST00000400554,3 | | protein_coding | | tsl1 | -8,818 | | 1,78E-05 |  |
| SCP2 | ENST00000371509,8 | | protein_coding | | tsl1 | -8,844 | | 1,67E-05 |  |
| MARK1 | ENST00000402574,5 | | protein_coding | | tsl1 | -8,862 | | 1,60E-05 |  |
| ZMAT1 | ENST00000372782,4 | | protein_coding | | tsl1 | -8,888 | | 1,49E-05 |  |
| VPS8 | ENST00000287546,8 | | protein_coding | | tsl1 | -8,888 | | 1,49E-05 |  |
| CCDC126 | ENST00000409765,5 | | protein_coding | | tsl1 | -8,902 | | 1,44E-05 |  |
| N4BP2 | ENST00000513269,1 | | protein_coding | | tsl1 | -8,918 | | 1,39E-05 |  |
| ZSWIM9 | ENST00000328759,11 | | protein_coding | | tsl1 | -8,918 | | 1,39E-05 |  |
| NFATC1 | ENST00000427363,6 | | protein_coding | | tsl1 | -8,934 | | 1,33E-05 |  |
| RAB7A | ENST00000482525,5 | | protein_coding | | tsl1 | -8,935 | | 1,33E-05 |  |
| PROM1 | ENST00000447510,6 | | protein_coding | | tsl1 | -8,959 | | 1,25E-05 |  |
| RPS6KA2 | ENST00000481261,6 | | protein_coding | | tsl1 | -8,988 | | 1,16E-05 |  |
| MED16 | ENST00000312090,10 | | protein_coding | | tsl1 | -8,989 | | 4,20E-06 |  |
| ARNTL2 | ENST00000395901,6 | | protein_coding | | tsl1 | -8,990 | | 1,16E-05 |  |
| BID | ENST00000399774,7 | | protein_coding | | tsl1 | -9,032 | | 1,04E-05 |  |
| MBD4 | ENST00000249910,5 | | protein_coding | | tsl1 | -9,033 | | 1,04E-05 |  |
| GTDC1 | ENST00000409214,5 | | protein_coding | | tsl1 | -9,037 | | 1,03E-05 |  |
| ECHDC1 | ENST00000528402,5 | | protein_coding | | tsl1 | -9,094 | | 8,89E-06 |  |
| GPBP1 | ENST00000264779,6 | | protein_coding | | tsl1 | -9,094 | | 8,88E-06 |  |
| SMG6 | ENST00000354901,8 | | protein_coding | | tsl1 | -9,095 | | 8,85E-06 |  |
| TOM1L1 | ENST00000575882,5 | | protein_coding | | tsl1 | -9,119 | | 1,12E-06 |  |
| NFATC2 | ENST00000396009,7 | | protein_coding | | tsl1 | -9,140 | | 4,56E-06 |  |
| MAP2 | ENST00000447185,5 | | protein_coding | | tsl1 | -9,142 | | 7,85E-06 |  |
| PBX2 | ENST00000420432,2 | | protein_coding | | tsl1 | -9,145 | | 7,80E-06 |  |
| PHF20 | ENST00000374000,8 | | protein_coding | | tsl1 | -9,155 | | 7,61E-06 |  |
| WSB2 | ENST00000535496,5 | | protein_coding | | tsl1 | -9,192 | | 6,92E-06 |  |
| ZNF423 | ENST00000262383,6 | | protein_coding | | tsl1 | -9,208 | | 6,64E-06 |  |
| STARD13 | ENST00000255486,8 | | protein_coding | | tsl1 | -9,250 | | 5,97E-06 |  |
| RBBP4 | ENST00000373485,5 | | protein_coding | | tsl1 | -9,258 | | 5,84E-06 |  |
| TTC7A | ENST00000394850,6 | | protein_coding | | tsl1 | -9,286 | | 5,43E-06 |  |
| TBC1D2 | ENST00000375064,5 | | protein_coding | | tsl1 | -9,287 | | 5,43E-06 |  |
| ETV1 | ENST00000405192,6 | | protein_coding | | tsl1 | -9,304 | | 5,17E-06 |  |
| PANK4 | ENST00000378466,7 | | protein_coding | | tsl1 | -9,331 | | 4,85E-06 |  |
| DNAH1 | ENST00000420323,6 | | protein_coding | | tsl1 | -9,351 | | 4,61E-06 |  |
| TRPS1 | ENST00000519674,1 | | protein_coding | | tsl1 | -9,397 | | 4,10E-06 |  |
| PRDM10 | ENST00000358825,9 | | protein_coding | | tsl1 | -9,410 | | 3,96E-06 |  |
| RNF38 | ENST00000259605,10 | | protein_coding | | tsl1 | -9,420 | | 3,87E-06 |  |
| KIAA0895 | ENST00000338533,9 | | protein_coding | | tsl1 | -9,423 | | 3,11E-06 |  |
| ZNF202 | ENST00000530393,5 | | protein_coding | | tsl1 | -9,440 | | 3,67E-06 |  |
| NDUFV1 | ENST00000529927,5 | | protein_coding | | tsl1 | -9,444 | | 3,63E-06 |  |
| COL4A6 | ENST00000372216,8 | | protein_coding | | tsl1 | -9,454 | | 3,54E-06 |  |
| PTPRN2 | ENST00000389416,8 | | protein_coding | | tsl1 | -9,460 | | 2,28E-06 |  |
| PHLDB1 | ENST00000600882,5 | | protein_coding | | tsl1 | -9,473 | | 3,37E-06 |  |
| DAPK1 | ENST00000358077,9 | | protein_coding | | tsl1 | -9,481 | | 1,40E-06 |  |
| PABPC1 | ENST00000610907,1 | | protein_coding | | tsl1 | -9,491 | | 3,22E-06 |  |
| KIF16B | ENST00000636835,1 | | protein_coding | | tsl1 | -9,495 | | 3,18E-06 |  |
| RAP1GAP2 | ENST00000366401,8 | | protein_coding | | tsl1 | -9,505 | | 2,07E-06 |  |
| NUCB2 | ENST00000458064,6 | | protein_coding | | tsl1 | -9,511 | | 3,05E-06 |  |
| TRAPPC11 | ENST00000512476,1 | | protein_coding | | tsl1 | -9,530 | | 2,90E-06 |  |
| RASSF7 | ENST00000397583,7 | | protein_coding | | tsl1 | -9,542 | | 2,82E-06 |  |
| TCAIM | ENST00000342649,8 | | protein_coding | | tsl1 | -9,566 | | 2,65E-06 |  |
| EZH2 | ENST00000320356,6 | | protein_coding | | tsl1 | -9,578 | | 2,57E-06 |  |
| PHF19 | ENST00000616568,4 | | protein_coding | | tsl1 | -9,598 | | 2,44E-06 |  |
| TRIM25 | ENST00000537230,2 | | protein_coding | | tsl1 | -9,599 | | 2,43E-06 |  |
| C5orf24 | ENST00000338051,4 | | protein_coding | | tsl1 | -9,603 | | 2,41E-06 |  |
| MYH10 | ENST00000379980,8 | | protein_coding | | tsl1 | -9,670 | | 2,03E-06 |  |
| MARK2 | ENST00000408948,7 | | protein_coding | | tsl1 | -9,680 | | 1,98E-06 |  |
| DRAM2 | ENST00000539140,5 | | protein_coding | | tsl1 | -9,700 | | 1,87E-06 |  |
| LASP1 | ENST00000435347,7 | | protein_coding | | tsl1 | -9,722 | | 1,77E-06 |  |
| ZSCAN26 | ENST00000421553,6 | | protein_coding | | tsl1 | -9,741 | | 1,69E-06 |  |
| BTBD3 | ENST00000618296,4 | | protein_coding | | tsl1 | -9,759 | | 1,61E-06 |  |
| CLN8 | ENST00000331222,5 | | protein_coding | | tsl1 | -9,766 | | 1,53E-06 |  |
| AKAP9 | ENST00000358100,6 | | protein_coding | | tsl1 | -9,828 | | 1,34E-06 |  |
| TARBP2 | ENST00000456234,6 | | protein_coding | | tsl1 | -9,912 | | 1,08E-06 |  |
| LIN54 | ENST00000442461,6 | | protein_coding | | tsl1 | -10,034 | | 7,86E-07 |  |
| ST3GAL5 | ENST00000377332,8 | | protein_coding | | tsl1 | -10,108 | | 6,48E-07 |  |
| COA6 | ENST00000619305,1 | | protein_coding | | tsl1 | -10,154 | | 5,74E-07 |  |
| NAGK | ENST00000613852,4 | | protein_coding | | tsl1 | -10,164 | | 5,61E-07 |  |
| GMPR2 | ENST00000559836,5 | | protein_coding | | tsl1 | -10,164 | | 5,38E-07 |  |
| ATP2C1 | ENST00000422190,6 | | protein_coding | | tsl1 | -10,165 | | 1,20E-08 |  |
| NSD2 | ENST00000503128,5 | | protein_coding | | tsl1 | -10,220 | | 2,90E-07 |  |
| DYSF | ENST00000409582,7 | | protein_coding | | tsl1 | -10,248 | | 4,49E-07 |  |
| USP25 | ENST00000400183,6 | | protein_coding | | tsl1 | -10,253 | | 4,44E-07 |  |
| RNF145 | ENST00000518802,5 | | protein_coding | | tsl1 | -10,287 | | 4,06E-07 |  |
| SH3BP5 | ENST00000408919,7 | | protein_coding | | tsl1 | -10,310 | | 3,82E-07 |  |
| SYNE2 | ENST00000344113,8 | | protein_coding | | tsl1 | -10,350 | | 3,44E-07 |  |
| ZNF827 | ENST00000513320,5 | | protein_coding | | tsl1 | -10,459 | | 2,35E-07 |  |
| DNM1L | ENST00000547312,5 | | protein_coding | | tsl1 | -10,472 | | 2,48E-07 |  |
| TYK2 | ENST00000524462,5 | | protein_coding | | tsl1 | -10,489 | | 2,39E-07 |  |
| MMP2 | ENST00000437642,6 | | protein_coding | | tsl1 | -10,492 | | 2,37E-07 |  |
| DYSF | ENST00000409366,5 | | protein_coding | | tsl1 | -10,635 | | 8,65E-09 |  |
| SRGAP2 | ENST00000624873,3 | | protein_coding | | tsl1 | -10,970 | | 6,17E-08 |  |
| ZNF410 | ENST00000555044,5 | | protein_coding | | tsl1 | -11,252 | | 3,04E-08 |  |
| ATP6V0A1 | ENST00000343619,8 | | protein_coding | | tsl1 | -11,403 | | 2,13E-08 |  |
| REV1 | ENST00000393445,7 | | protein_coding | | tsl1 | -11,482 | | 1,58E-08 |  |

**Supplemental Table S3. List of the first 100 upregulated DEGs in High fat (HF) treatment.**

| gene name | target id | transcript  biotype | transcript  tsl | H-FAT.  log FC | H-FAT.  PValue |
| --- | --- | --- | --- | --- | --- |
| TAB3 | ENST00000378933,5 | protein_coding | tsl1 | 12,067 | 4,31E-09 |
| ZC3H11A | ENST00000332127,8 | protein_coding | tsl1 | 11,873 | 7,47E-09 |
| LRRC8B | ENST00000439853,5 | protein_coding | tsl1 | 11,848 | 7,91E-09 |
| TBCK | ENST00000394706,7 | protein_coding | tsl1 | 11,753 | 1,03E-08 |
| KIAA0232 | ENST00000307659,5 | protein_coding | tsl1 | 11,283 | 3,58E-08 |
| SMG7 | ENST00000507469,5 | protein_coding | tsl1 | 10,829 | 1,19E-07 |
| SART3 | ENST00000431469,6 | protein_coding | tsl1 | 10,684 | 1,74E-07 |
| RAPGEF1 | ENST00000372190,7 | protein_coding | tsl1 | 10,642 | 9,70E-09 |
| WDR81 | ENST00000632244,1 | protein_coding | tsl1 | 10,519 | 2,69E-07 |
| EVL | ENST00000392920,7 | protein_coding | tsl1 | 10,500 | 2,83E-07 |
| ZFAT | ENST00000520727,5 | protein_coding | tsl1 | 10,469 | 3,07E-07 |
| PAM | ENST00000304400,11 | protein_coding | tsl1 | 10,412 | 3,56E-07 |
| ATP6V0A1 | ENST00000343619,8 | protein_coding | tsl1 | 10,393 | 3,46E-07 |
| SFI1 | ENST00000432498,5 | protein_coding | tsl1 | 10,308 | 4,66E-07 |
| CARD8 | ENST00000520753,5 | protein_coding | tsl1 | 10,298 | 4,80E-07 |
| STK17B | ENST00000409228,5 | protein_coding | tsl1 | 10,259 | 5,32E-07 |
| BTBD3 | ENST00000618296,4 | protein_coding | tsl1 | 10,257 | 7,83E-08 |
| SP110 | ENST00000392048,7 | protein_coding | tsl1 | 10,203 | 6,16E-07 |
| CLN8 | ENST00000331222,5 | protein_coding | tsl1 | 10,194 | 1,86E-07 |
| LASP1 | ENST00000435347,7 | protein_coding | tsl1 | 10,172 | 6,06E-07 |
| AP4E1 | ENST00000560508,1 | protein_coding | tsl1 | 10,169 | 5,83E-07 |
| RAPGEF1 | ENST00000372189,7 | protein_coding | tsl1 | 10,148 | 7,09E-07 |
| ARHGAP12 | ENST00000396144,8 | protein_coding | tsl1 | 10,147 | 3,75E-07 |
| YY1AP1 | ENST00000347088,9 | protein_coding | tsl1 | 10,098 | 7,95E-07 |
| SCAMP5 | ENST00000562212,5 | protein_coding | tsl1 | 9,990 | 1,07E-06 |
| NEK2 | ENST00000540251,5 | protein_coding | tsl1 | 9,962 | 1,01E-06 |
| CLDND1 | ENST00000394181,6 | protein_coding | tsl1 | 9,958 | 1,17E-06 |
| MARK2 | ENST00000408948,7 | protein_coding | tsl1 | 9,957 | 1,17E-06 |
| PTDSS2 | ENST00000633628,1 | protein_coding | tsl1 | 9,929 | 1,26E-06 |
| DEAF1 | ENST00000632113,1 | protein_coding | tsl1 | 9,900 | 1,35E-06 |
| ACACA | ENST00000613687,4 | protein_coding | tsl1 | 9,884 | 8,49E-07 |
| KIF14 | ENST00000614960,4 | protein_coding | tsl1 | 9,882 | 1,42E-06 |
| ATP13A1 | ENST00000291503,9 | protein_coding | tsl1 | 9,809 | 1,72E-06 |
| MTRF1L | ENST00000367231,9 | protein_coding | tsl1 | 9,743 | 5,30E-07 |
| DXO | ENST00000375356,7 | protein_coding | tsl1 | 9,711 | 2,21E-06 |
| PAAF1 | ENST00000544552,5 | protein_coding | tsl1 | 9,700 | 2,28E-06 |
| ARNTL | ENST00000401424,5 | protein_coding | tsl1 | 9,697 | 1,72E-06 |
| DGLUCY | ENST00000521077,6 | protein_coding | tsl1 | 9,689 | 2,34E-06 |
| FTO | ENST00000463855,1 | protein_coding | tsl1 | 9,688 | 2,35E-06 |
| CHD2 | ENST00000420239,6 | protein_coding | tsl1 | 9,684 | 2,37E-06 |
| TMEM143 | ENST00000377431,6 | protein_coding | tsl1 | 9,673 | 2,44E-06 |
| BZW2 | ENST00000415365,5 | protein_coding | tsl1 | 9,673 | 2,43E-06 |
| CTAGE5 | ENST00000280083,7 | protein_coding | tsl1 | 9,668 | 2,47E-06 |
| TJP2 | ENST00000348208,8 | protein_coding | tsl1 | 9,667 | 2,36E-06 |
| SLC39A10 | ENST00000409086,7 | protein_coding | tsl1 | 9,662 | 2,51E-06 |
| CPT1B | ENST00000312108,11 | protein_coding | tsl1 | 9,662 | 8,51E-08 |
| MARC2 | ENST00000359316,6 | protein_coding | tsl1 | 9,578 | 3,12E-06 |
| DAPK1 | ENST00000358077,9 | protein_coding | tsl1 | 9,575 | 2,67E-06 |
| FBXL15 | ENST00000224862,7 | protein_coding | tsl1 | 9,572 | 2,12E-06 |
| GAB2 | ENST00000340149,6 | protein_coding | tsl1 | 9,558 | 3,28E-06 |
| PITPNC1 | ENST00000580974,5 | protein_coding | tsl1 | 9,548 | 3,36E-06 |
| PML | ENST00000567606,5 | protein_coding | tsl1 | 9,521 | 3,60E-06 |
| PRDM10 | ENST00000358825,9 | protein_coding | tsl1 | 9,516 | 3,65E-06 |
| ITPKB | ENST00000272117,7 | protein_coding | tsl1 | 9,465 | 4,16E-06 |
| KIF21A | ENST00000541463,6 | protein_coding | tsl1 | 9,445 | 4,38E-06 |
| ICA1 | ENST00000396675,7 | protein_coding | tsl1 | 9,419 | 4,68E-06 |
| STX16 | ENST00000358029,8 | protein_coding | tsl1 | 9,414 | 4,75E-06 |
| DTX2 | ENST00000324432,9 | protein_coding | tsl1 | 9,408 | 4,82E-06 |
| TMEM237 | ENST00000621467,4 | protein_coding | tsl1 | 9,400 | 2,88E-06 |
| RCAN1 | ENST00000399272,5 | protein_coding | tsl1 | 9,395 | 4,98E-06 |
| SYTL5 | ENST00000456733,2 | protein_coding | tsl1 | 9,388 | 5,08E-06 |
| SRPK2 | ENST00000489828,5 | protein_coding | tsl1 | 9,384 | 5,13E-06 |
| ZNF18 | ENST00000580613,5 | protein_coding | tsl1 | 9,363 | 5,41E-06 |
| CAMSAP2 | ENST00000236925,8 | protein_coding | tsl1 | 9,355 | 5,51E-06 |
| OLFML2A | ENST00000288815,5 | protein_coding | tsl1 | 9,295 | 6,44E-06 |
| SEMA3F | ENST00000413852,5 | protein_coding | tsl1 | 9,292 | 6,48E-06 |
| FAM213A | ENST00000372187,9 | protein_coding | tsl1 | 9,265 | 6,95E-06 |
| DDX31 | ENST00000372153,5 | protein_coding | tsl1 | 9,254 | 7,14E-06 |
| RFXANK | ENST00000303088,8 | protein_coding | tsl1 | 9,241 | 7,01E-06 |
| ZEB1 | ENST00000361642,9 | protein_coding | tsl1 | 9,235 | 5,30E-06 |
| ERBB4 | ENST00000436443,5 | protein_coding | tsl1 | 9,216 | 7,87E-06 |
| SLC43A1 | ENST00000528450,5 | protein_coding | tsl1 | 9,215 | 7,88E-06 |
| GLI1 | ENST00000528467,1 | protein_coding | tsl1 | 9,212 | 7,94E-06 |
| PISD | ENST00000266095,9 | protein_coding | tsl1 | 9,201 | 1,52E-06 |
| SH3BP4 | ENST00000344528,8 | protein_coding | tsl1 | 9,177 | 7,71E-06 |
| ST6GAL1 | ENST00000448044,5 | protein_coding | tsl1 | 9,166 | 8,93E-06 |
| FYN | ENST00000229471,8 | protein_coding | tsl1 | 9,165 | 8,96E-06 |
| ZNF439 | ENST00000455282,1 | protein_coding | tsl1 | 9,119 | 1,01E-05 |
| NHLRC3 | ENST00000470258,5 | protein_coding | tsl1 | 9,111 | 1,03E-05 |
| UCHL5 | ENST00000367448,5 | protein_coding | tsl1 | 9,108 | 1,03E-05 |
| IRF3 | ENST00000593922,5 | protein_coding | tsl1 | 9,097 | 1,06E-05 |
| MCRS1 | ENST00000546244,5 | protein_coding | tsl1 | 9,074 | 1,13E-05 |
| LTN1 | ENST00000389194,6 | protein_coding | tsl1 | 9,070 | 2,88E-06 |
| MTERF4 | ENST00000614476,4 | protein_coding | tsl1 | 9,064 | 3,17E-07 |
| CERS1 | ENST00000623882,3 | protein_coding | tsl1 | 9,053 | 1,18E-05 |
| GDF1 | ENST00000247005,7 | protein_coding | tsl1 | 9,053 | 1,18E-05 |
| RDH10 | ENST00000519380,1 | protein_coding | tsl1 | 9,047 | 1,21E-05 |
| RBPJ | ENST00000507561,5 | protein_coding | tsl1 | 9,039 | 4,87E-06 |
| WNT5B | ENST00000397196,6 | protein_coding | tsl1 | 9,008 | 1,33E-05 |
| ACTR3C | ENST00000478393,5 | protein_coding | tsl1 | 9,008 | 1,33E-05 |
| CYB561 | ENST00000392975,6 | protein_coding | tsl1 | 9,007 | 1,33E-05 |
| TMCO6 | ENST00000252100,6 | protein_coding | tsl1 | 9,001 | 1,35E-05 |
| CRMP1 | ENST00000397890,6 | protein_coding | tsl1 | 8,992 | 1,39E-05 |
| FERMT1 | ENST00000536936,1 | protein_coding | tsl1 | 8,965 | 1,48E-05 |
| AGAP5 | ENST00000443782,6 | protein_coding | tsl1 | 8,955 | 1,51E-05 |
| MIER1 | ENST00000371018,7 | protein_coding | tsl1 | 8,922 | 1,65E-05 |
| ECT2 | ENST00000417960,5 | protein_coding | tsl1 | 8,921 | 1,66E-05 |
| ARID3B | ENST00000622429,1 | protein_coding | tsl1 | 8,898 | 1,75E-05 |
| WDR46 | ENST00000457382,6 | protein_coding | tsl1 | 8,874 | 4,73E-06 |

**Supplemental Table S4. List of the first 100 downregulated DEGs in High fat (HF) treatment.**

| gene name | target id | transcript  biotype | transcript  tsl | H-FAT.  log FC | H-FAT.  PValue |
| --- | --- | --- | --- | --- | --- |
| PRIMPOL | ENST00000515774,5 | protein_coding | tsl1 | -8,560 | 2,78E-05 |
| CELF2 | ENST00000416382,6 | protein_coding | tsl1 | -8,602 | 2,51E-05 |
| PARP8 | ENST00000514067,6 | protein_coding | tsl1 | -8,604 | 2,39E-05 |
| IL11RA | ENST00000318041,13 | protein_coding | tsl1 | -8,609 | 2,47E-05 |
| MAGED4 | ENST00000599522,6 | protein_coding | tsl1 | -8,616 | 2,42E-05 |
| ACY1 | ENST00000404366,7 | protein_coding | tsl1 | -8,619 | 2,41E-05 |
| ACADVL | ENST00000350303,9 | protein_coding | tsl1 | -8,622 | 2,39E-05 |
| APAF1 | ENST00000359972,6 | protein_coding | tsl1 | -8,646 | 2,25E-05 |
| SYNE2 | ENST00000344113,8 | protein_coding | tsl1 | -8,647 | 2,24E-05 |
| ZNF654 | ENST00000309495,6 | protein_coding | tsl1 | -8,662 | 2,16E-05 |
| PTPN2 | ENST00000591115,5 | protein_coding | tsl1 | -8,662 | 2,16E-05 |
| C2CD2 | ENST00000329623,11 | protein_coding | tsl1 | -8,662 | 2,16E-05 |
| ANGEL2 | ENST00000535388,2 | protein_coding | tsl1 | -8,667 | 2,13E-05 |
| SPOCK3 | ENST00000357545,8 | protein_coding | tsl1 | -8,684 | 2,04E-05 |
| CCDC112 | ENST00000395557,4 | protein_coding | tsl1 | -8,693 | 2,00E-05 |
| PCDH9 | ENST00000456367,5 | protein_coding | tsl1 | -8,709 | 1,92E-05 |
| LRRC32 | ENST00000407242,6 | protein_coding | tsl1 | -8,711 | 1,91E-05 |
| PELP1 | ENST00000301396,8 | protein_coding | tsl1 | -8,731 | 1,81E-05 |
| EARS2 | ENST00000563232,1 | protein_coding | tsl1 | -8,759 | 1,69E-05 |
| PGRMC2 | ENST00000613358,4 | protein_coding | tsl1 | -8,775 | 1,63E-05 |
| DENND2A | ENST00000537639,5 | protein_coding | tsl1 | -8,787 | 4,22E-07 |
| DDX6 | ENST00000526070,2 | protein_coding | tsl1 | -8,799 | 1,53E-05 |
| GK | ENST00000378946,7 | protein_coding | tsl1 | -8,818 | 1,46E-05 |
| RBM33 | ENST00000341148,7 | protein_coding | tsl1 | -8,820 | 1,44E-05 |
| AKAP12 | ENST00000359755,5 | protein_coding | tsl1 | -8,829 | 1,42E-05 |
| FAM222B | ENST00000582266,5 | protein_coding | tsl1 | -8,831 | 1,17E-05 |
| HMGB3 | ENST00000430118,1 | protein_coding | tsl1 | -8,861 | 1,28E-05 |
| USP36 | ENST00000449938,6 | protein_coding | tsl1 | -8,865 | 1,29E-05 |
| COMMD10 | ENST00000632434,1 | protein_coding | tsl1 | -8,881 | 9,99E-07 |
| ARFGAP1 | ENST00000353546,7 | protein_coding | tsl1 | -8,885 | 3,76E-07 |
| LDB3 | ENST00000263066,10 | protein_coding | tsl1 | -8,904 | 1,17E-05 |
| GNL1 | ENST00000441604,5 | protein_coding | tsl1 | -8,910 | 1,15E-05 |
| NAA60 | ENST00000572584,1 | protein_coding | tsl1 | -8,931 | 1,09E-05 |
| ANTXR1 | ENST00000409349,7 | protein_coding | tsl1 | -8,960 | 1,02E-05 |
| USP28 | ENST00000537706,5 | protein_coding | tsl1 | -8,963 | 1,01E-05 |
| USP21 | ENST00000368001,1 | protein_coding | tsl1 | -8,968 | 9,95E-06 |
| ACAP3 | ENST00000353662,4 | protein_coding | tsl1 | -9,006 | 9,04E-06 |
| NR1H3 | ENST00000395397,7 | protein_coding | tsl1 | -9,013 | 8,88E-06 |
| NF1 | ENST00000356175,7 | protein_coding | tsl1 | -9,035 | 3,90E-06 |
| AKT3 | ENST00000621586,3 | protein_coding | tsl1 | -9,049 | 4,39E-08 |
| RSRP1 | ENST00000431849,3 | protein_coding | tsl1 | -9,088 | 7,34E-06 |
| ARID1B | ENST00000637810,1 | protein_coding | tsl1 | -9,099 | 7,12E-06 |
| TBC1D2 | ENST00000375064,5 | protein_coding | tsl1 | -9,100 | 7,11E-06 |
| CNTRL | ENST00000238341,9 | protein_coding | tsl1 | -9,129 | 6,61E-06 |
| E2F5 | ENST00000416274,6 | protein_coding | tsl1 | -9,144 | 6,35E-06 |
| CSRNP3 | ENST00000342316,8 | protein_coding | tsl1 | -9,159 | 6,12E-06 |
| SMG6 | ENST00000354901,8 | protein_coding | tsl1 | -9,174 | 5,88E-06 |
| EIF4G1 | ENST00000382330,7 | protein_coding | tsl1 | -9,189 | 3,71E-06 |
| GRAMD4 | ENST00000406902,5 | protein_coding | tsl1 | -9,203 | 5,46E-06 |
| LDLRAD3 | ENST00000528989,5 | protein_coding | tsl1 | -9,207 | 4,87E-06 |
| CPEB4 | ENST00000517880,1 | protein_coding | tsl1 | -9,243 | 4,93E-06 |
| C4A | ENST00000375295,8 | protein_coding | tsl1 | -9,323 | 4,01E-06 |
| CEP162 | ENST00000257766,8 | protein_coding | tsl1 | -9,330 | 3,94E-06 |
| LSR | ENST00000605618,5 | protein_coding | tsl1 | -9,367 | 3,59E-06 |
| RC3H1 | ENST00000258349,8 | protein_coding | tsl1 | -9,373 | 2,95E-08 |
| HNRNPK | ENST00000376263,7 | protein_coding | tsl1 | -9,391 | 3,37E-06 |
| AKNA | ENST00000374075,9 | protein_coding | tsl1 | -9,391 | 3,36E-06 |
| RPS6KA2 | ENST00000481261,6 | protein_coding | tsl1 | -9,413 | 1,70E-06 |
| PCDH18 | ENST00000507846,5 | protein_coding | tsl1 | -9,434 | 3,01E-06 |
| SH3BP2 | ENST00000356331,9 | protein_coding | tsl1 | -9,493 | 2,59E-06 |
| SGK3 | ENST00000396596,2 | protein_coding | tsl1 | -9,498 | 2,55E-06 |
| FAM192A | ENST00000389447,9 | protein_coding | tsl1 | -9,513 | 2,46E-06 |
| ZBTB22 | ENST00000418540,2 | protein_coding | tsl1 | -9,539 | 2,27E-06 |
| ZBTB22 | ENST00000416097,2 | protein_coding | tsl1 | -9,539 | 2,27E-06 |
| NDRG1 | ENST00000522476,5 | protein_coding | tsl1 | -9,540 | 2,29E-06 |
| ZNF630 | ENST00000276054,8 | protein_coding | tsl1 | -9,553 | 2,21E-06 |
| GMPR2 | ENST00000559836,5 | protein_coding | tsl1 | -9,625 | 1,83E-06 |
| GSDME | ENST00000419307,5 | protein_coding | tsl1 | -9,635 | 1,79E-06 |
| KIAA0586 | ENST00000423743,7 | protein_coding | tsl1 | -9,639 | 1,17E-06 |
| DDX27 | ENST00000622530,4 | protein_coding | tsl1 | -9,660 | 1,68E-06 |
| ZNF473 | ENST00000391821,6 | protein_coding | tsl1 | -9,675 | 1,61E-06 |
| NAPB | ENST00000398425,7 | protein_coding | tsl1 | -9,691 | 1,55E-06 |
| LRP8 | ENST00000371454,6 | protein_coding | tsl1 | -9,817 | 9,82E-07 |
| MLST8 | ENST00000569417,5 | protein_coding | tsl1 | -9,827 | 9,90E-07 |
| STEAP2 | ENST00000287908,7 | protein_coding | tsl1 | -9,836 | 1,06E-06 |
| EPS8L2 | ENST00000526198,5 | protein_coding | tsl1 | -9,841 | 7,51E-07 |
| MID1 | ENST00000380780,5 | protein_coding | tsl1 | -9,863 | 9,89E-07 |
| AAAS | ENST00000394384,7 | protein_coding | tsl1 | -9,874 | 9,60E-07 |
| KIF27 | ENST00000334204,6 | protein_coding | tsl1 | -9,889 | 9,23E-07 |
| LATS1 | ENST00000253339,9 | protein_coding | tsl1 | -9,905 | 8,85E-07 |
| SSBP1 | ENST00000571430,5 | protein_coding | tsl1 | -9,977 | 6,68E-07 |
| SOCS4 | ENST00000339298,2 | protein_coding | tsl1 | -9,988 | 7,12E-07 |
| ZNF569 | ENST00000392149,6 | protein_coding | tsl1 | -10,054 | 5,99E-07 |
| SEPT2 | ENST00000360051,7 | protein_coding | tsl1 | -10,088 | 4,11E-07 |
| TDRD3 | ENST00000535286,5 | protein_coding | tsl1 | -10,096 | 5,38E-07 |
| EIF4A1 | ENST00000577269,5 | protein_coding | tsl1 | -10,143 | 4,75E-07 |
| SLC6A9 | ENST00000357730,6 | protein_coding | tsl1 | -10,246 | 3,62E-07 |
| SLC26A11 | ENST00000572725,5 | protein_coding | tsl1 | -10,253 | 3,56E-07 |
| LNPK | ENST00000544803,5 | protein_coding | tsl1 | -10,423 | 2,27E-07 |
| LAMA4 | ENST00000522006,5 | protein_coding | tsl1 | -10,659 | 1,19E-07 |
| MBNL1 | ENST00000492948,5 | protein_coding | tsl1 | -10,746 | 9,68E-08 |
| ZMYND8 | ENST00000461685,5 | protein_coding | tsl1 | -10,764 | 9,23E-08 |
| PPIP5K1 | ENST00000334933,8 | protein_coding | tsl1 | -10,780 | 8,85E-08 |
| ARMC10 | ENST00000428183,6 | protein_coding | tsl1 | -10,957 | 5,41E-08 |
| KAT14 | ENST00000489634,2 | protein_coding | tsl1 | -11,031 | 4,54E-08 |
| CPNE2 | ENST00000535318,6 | protein_coding | tsl1 | -11,104 | 3,71E-08 |
| SERPINA5 | ENST00000554276,1 | protein_coding | tsl1 | -11,200 | 2,90E-08 |
| ACIN1 | ENST00000397341,7 | protein_coding | tsl1 | -11,287 | 2,05E-08 |
| MAMLD1 | ENST00000426613,4 | protein_coding | tsl1 | -11,362 | 1,87E-08 |
| LONP1 | ENST00000590729,5 | protein_coding | tsl1 | -11,731 | 7,08E-09 |

**Supplemental Table S5. List of the first 100 upregulated DEGs in Insulin (INS) treatment.**

| gene name | target id | transcript  biotype | transcript  tsl | Ins.  log FC | Ins.  PValue |
| --- | --- | --- | --- | --- | --- |
| TBCK | ENST00000394706.7 | protein_coding | tsl1 | 11,712 | 1,16E-08 |
| ATP2B4 | ENST00000341360.6 | protein_coding | tsl1 | 11,497 | 1,67E-08 |
| ZC3H11A | ENST00000332127.8 | protein_coding | tsl1 | 11,456 | 2,28E-08 |
| ATP6V0A1 | ENST00000343619.8 | protein_coding | tsl1 | 11,439 | 2,20E-08 |
| KDM6A | ENST00000536777.5 | protein_coding | tsl1 | 10,670 | 1,72E-07 |
| WDR81 | ENST00000632244.1 | protein_coding | tsl1 | 10,465 | 3,13E-07 |
| CLDND1 | ENST00000394181.6 | protein_coding | tsl1 | 10,456 | 3,20E-07 |
| NRF1 | ENST00000393230.6 | protein_coding | tsl1 | 10,392 | 3,79E-07 |
| RNF145 | ENST00000518802.5 | protein_coding | tsl1 | 10,346 | 4,14E-07 |
| UHRF1 | ENST00000620565.4 | protein_coding | tsl1 | 10,345 | 4,30E-07 |
| DYSF | ENST00000409582.7 | protein_coding | tsl1 | 10,326 | 4,52E-07 |
| KIF1B | ENST00000377083.5 | protein_coding | tsl1 | 10,325 | 4,52E-07 |
| PIK3R3 | ENST00000420542.5 | protein_coding | tsl1 | 10,136 | 7,42E-07 |
| SEPT6 | ENST00000394610.5 | protein_coding | tsl1 | 10,100 | 8,15E-07 |
| SLC39A10 | ENST00000409086.7 | protein_coding | tsl1 | 10,091 | 8,35E-07 |
| KIAA0232 | ENST00000307659.5 | protein_coding | tsl1 | 10,002 | 1,05E-06 |
| CARD8 | ENST00000520753.5 | protein_coding | tsl1 | 9,973 | 1,13E-06 |
| AP1B1 | ENST00000357586.6 | protein_coding | tsl1 | 9,969 | 7,17E-09 |
| TAB3 | ENST00000378933.5 | protein_coding | tsl1 | 9,911 | 1,29E-06 |
| DAPK1 | ENST00000358077.9 | protein_coding | tsl1 | 9,902 | 1,16E-06 |
| STAU2 | ENST00000519961.5 | protein_coding | tsl1 | 9,876 | 1,46E-06 |
| CHD2 | ENST00000420239.6 | protein_coding | tsl1 | 9,827 | 1,66E-06 |
| SFI1 | ENST00000432498.5 | protein_coding | tsl1 | 9,760 | 1,96E-06 |
| MARK2 | ENST00000408948.7 | protein_coding | tsl1 | 9,756 | 1,99E-06 |
| LASP1 | ENST00000435347.7 | protein_coding | tsl1 | 9,749 | 1,84E-06 |
| ARHGAP12 | ENST00000396144.8 | protein_coding | tsl1 | 9,742 | 1,09E-06 |
| TRIM25 | ENST00000537230.2 | protein_coding | tsl1 | 9,676 | 2,45E-06 |
| DDX31 | ENST00000372153.5 | protein_coding | tsl1 | 9,663 | 2,53E-06 |
| ATP13A1 | ENST00000291503.9 | protein_coding | tsl1 | 9,576 | 3,17E-06 |
| ZFX | ENST00000539115.5 | protein_coding | tsl1 | 9,560 | 3,27E-06 |
| LIPH | ENST00000424591.6 | protein_coding | tsl1 | 9,549 | 3,40E-06 |
| COL4A6 | ENST00000372216.8 | protein_coding | tsl1 | 9,531 | 3,56E-06 |
| NHLRC3 | ENST00000470258.5 | protein_coding | tsl1 | 9,513 | 3,73E-06 |
| FAM213A | ENST00000372187.9 | protein_coding | tsl1 | 9,503 | 3,83E-06 |
| ZMYND11 | ENST00000381584.5 | protein_coding | tsl1 | 9,502 | 3,83E-06 |
| RNF38 | ENST00000259605.10 | protein_coding | tsl1 | 9,497 | 3,89E-06 |
| ZNF439 | ENST00000455282.1 | protein_coding | tsl1 | 9,488 | 3,97E-06 |
| PRDM10 | ENST00000358825.9 | protein_coding | tsl1 | 9,487 | 3,98E-06 |
| AP4E1 | ENST00000560508.1 | protein_coding | tsl1 | 9,422 | 4,10E-06 |
| ZEB1 | ENST00000361642.9 | protein_coding | tsl1 | 9,402 | 3,49E-06 |
| C16orf62 | ENST00000438132.7 | protein_coding | tsl1 | 9,363 | 5,47E-06 |
| SLC38A6 | ENST00000451406.5 | protein_coding | tsl1 | 9,352 | 5,63E-06 |
| STARD13 | ENST00000255486.8 | protein_coding | tsl1 | 9,327 | 6,01E-06 |
| NFATC2IP | ENST00000564978.5 | protein_coding | tsl1 | 9,290 | 6,60E-06 |
| DRAM2 | ENST00000539140.5 | protein_coding | tsl1 | 9,277 | 2,48E-06 |
| GTF3C3 | ENST00000409364.3 | protein_coding | tsl1 | 9,275 | 6,85E-06 |
| CLN8 | ENST00000331222.5 | protein_coding | tsl1 | 9,274 | 2,09E-06 |
| C2CD3 | ENST00000414160.6 | protein_coding | tsl1 | 9,252 | 7,28E-06 |
| SP110 | ENST00000392048.7 | protein_coding | tsl1 | 9,239 | 7,52E-06 |
| CERS1 | ENST00000623882.3 | protein_coding | tsl1 | 9,226 | 7,71E-06 |
| GDF1 | ENST00000247005.7 | protein_coding | tsl1 | 9,226 | 7,71E-06 |
| SYTL5 | ENST00000456733.2 | protein_coding | tsl1 | 9,209 | 8,11E-06 |
| SCN9A | ENST00000454569.5 | protein_coding | tsl1 | 9,208 | 1,26E-06 |
| ZNF23 | ENST00000428724.5 | protein_coding | tsl1 | 9,200 | 8,30E-06 |
| ZBTB17 | ENST00000375733.6 | protein_coding | tsl1 | 9,172 | 8,92E-06 |
| ECHDC1 | ENST00000528402.5 | protein_coding | tsl1 | 9,171 | 8,93E-06 |
| TSPOAP1 | ENST00000268893.10 | protein_coding | tsl1 | 9,130 | 9,91E-06 |
| PISD | ENST00000266095.9 | protein_coding | tsl1 | 9,087 | 2,07E-06 |
| FUT8 | ENST00000394586.6 | protein_coding | tsl1 | 9,081 | 1,12E-05 |
| OGDH | ENST00000443864.6 | protein_coding | tsl1 | 9,077 | 1,13E-05 |
| SCYL3 | ENST00000367770.5 | protein_coding | tsl1 | 9,065 | 1,17E-05 |
| ZNF45 | ENST00000589703.5 | protein_coding | tsl1 | 9,057 | 5,18E-06 |
| SGCE | ENST00000447873.5 | protein_coding | tsl1 | 9,053 | 1,20E-05 |
| AMT | ENST00000273588.8 | protein_coding | tsl1 | 9,038 | 1,25E-05 |
| PROM1 | ENST00000447510.6 | protein_coding | tsl1 | 9,036 | 1,26E-05 |
| TTC7A | ENST00000394850.6 | protein_coding | tsl1 | 9,031 | 6,57E-06 |
| CEP152 | ENST00000399334.7 | protein_coding | tsl1 | 9,004 | 1,36E-05 |
| ZSWIM9 | ENST00000328759.11 | protein_coding | tsl1 | 8,995 | 1,39E-05 |
| ATXN7 | ENST00000484332.1 | protein_coding | tsl1 | 8,990 | 1,41E-05 |
| EIF2S1 | ENST00000466499.6 | protein_coding | tsl1 | 8,981 | 1,44E-05 |
| PRDM1 | ENST00000369089.3 | protein_coding | tsl1 | 8,978 | 1,45E-05 |
| ZNF18 | ENST00000580613.5 | protein_coding | tsl1 | 8,967 | 1,50E-05 |
| ZMAT1 | ENST00000372782.4 | protein_coding | tsl1 | 8,965 | 1,50E-05 |
| SART3 | ENST00000431469.6 | protein_coding | tsl1 | 8,952 | 1,55E-05 |
| PRKAR1B | ENST00000406797.5 | protein_coding | tsl1 | 8,933 | 1,63E-05 |
| SLC22A18 | ENST00000610526.4 | protein_coding | tsl1 | 8,879 | 1,87E-05 |
| C9orf3 | ENST00000375315.6 | protein_coding | tsl1 | 8,875 | 1,88E-05 |
| TMEM237 | ENST00000621467.4 | protein_coding | tsl1 | 8,873 | 1,12E-05 |
| SLC22A17 | ENST00000637426.1 | protein_coding | tsl1 | 8,868 | 1,92E-05 |
| LRRC7 | ENST00000415775.2 | protein_coding | tsl1 | 8,854 | 1,98E-05 |
| ECT2 | ENST00000417960.5 | protein_coding | tsl1 | 8,852 | 2,00E-05 |
| PYGL | ENST00000532462.5 | protein_coding | tsl1 | 8,834 | 2,09E-05 |
| TMEM185A | ENST00000613273.4 | protein_coding | tsl1 | 8,832 | 2,10E-05 |
| APC2 | ENST00000238483.5 | protein_coding | tsl1 | 8,827 | 2,12E-05 |
| ACTR3C | ENST00000478393.5 | protein_coding | tsl1 | 8,797 | 2,29E-05 |
| ARID3B | ENST00000622429.1 | protein_coding | tsl1 | 8,783 | 2,37E-05 |
| ATRIP | ENST00000346691.9 | protein_coding | tsl1 | 8,761 | 2,51E-05 |
| TAP1 | ENST00000439781.2 | protein_coding | tsl1 | 8,753 | 2,56E-05 |
| PITPNC1 | ENST00000580974.5 | protein_coding | tsl1 | 8,750 | 2,57E-05 |
| MTMR3 | ENST00000351488.7 | protein_coding | tsl1 | 8,719 | 2,78E-05 |
| STK17B | ENST00000409228.5 | protein_coding | tsl1 | 8,715 | 2,80E-05 |
| HYAL2 | ENST00000357750.8 | protein_coding | tsl1 | 8,690 | 2,99E-05 |
| AGAP5 | ENST00000443782.6 | protein_coding | tsl1 | 8,682 | 3,04E-05 |
| SNX27 | ENST00000368838.1 | protein_coding | tsl1 | 8,678 | 3,08E-05 |
| MCRS1 | ENST00000357123.8 | protein_coding | tsl1 | 8,675 | 2,71E-05 |
| COQ8A | ENST00000366778.5 | protein_coding | tsl1 | 8,670 | 3,14E-05 |
| DOC2A | ENST00000564979.5 | protein_coding | tsl1 | 8,665 | 3,18E-05 |
| CDC42SE2 | ENST00000503291.5 | protein_coding | tsl1 | 8,662 | 3,20E-05 |
| MTERF4 | ENST00000614476.4 | protein_coding | tsl1 | 8,662 | 9,17E-07 |
| SEMA3F | ENST00000413852.5 | protein_coding | tsl1 | 8,650 | 3,30E-05 |

**Supplemental Table S6. List of the first 100 downregulated DEGs in Insulin (INS) treatment.**

| gene name | target id | transcript  biotype | | transcript  tsl | | | Ins.  log FC | | Ins.  PValue | |
| --- | --- | --- | --- | --- | --- | --- | --- | --- | --- | --- |
| CDC14A | ENST00000361544.10 | | protein_coding | | tsl1 | -8,611 | | 3,01E-05 | |  |
| BTN3A2 | ENST00000377708.6 | | protein_coding | | tsl1 | -8,614 | | 2,98E-05 | |  |
| ACY1 | ENST00000404366.7 | | protein_coding | | tsl1 | -8,622 | | 2,92E-05 | |  |
| SNX24 | ENST00000513881.5 | | protein_coding | | tsl1 | -8,627 | | 2,89E-05 | |  |
| ZNF566 | ENST00000493391.5 | | protein_coding | | tsl1 | -8,658 | | 2,65E-05 | |  |
| SLFN5 | ENST00000592325.1 | | protein_coding | | tsl1 | -8,661 | | 2,65E-05 | |  |
| LAMA3 | ENST00000399516.7 | | protein_coding | | tsl1 | -8,667 | | 2,61E-05 | |  |
| OSBPL9 | ENST00000453295.5 | | protein_coding | | tsl1 | -8,670 | | 2,59E-05 | |  |
| HERC4 | ENST00000412272.6 | | protein_coding | | tsl1 | -8,678 | | 2,54E-05 | |  |
| AMACR | ENST00000512079.5 | | protein_coding | | tsl1 | -8,702 | | 2,40E-05 | |  |
| LRRC32 | ENST00000407242.6 | | protein_coding | | tsl1 | -8,714 | | 2,32E-05 | |  |
| ZNF446 | ENST00000610298.1 | | protein_coding | | tsl1 | -8,716 | | 2,31E-05 | |  |
| NBPF3 | ENST00000318249.9 | | protein_coding | | tsl1 | -8,717 | | 2,31E-05 | |  |
| CRY2 | ENST00000616080.1 | | protein_coding | | tsl1 | -8,719 | | 2,30E-05 | |  |
| KCNAB2 | ENST00000352527.5 | | protein_coding | | tsl1 | -8,719 | | 2,29E-05 | |  |
| PTPN13 | ENST00000316707.10 | | protein_coding | | tsl1 | -8,746 | | 2,14E-05 | |  |
| TCTN1 | ENST00000551590.5 | | protein_coding | | tsl1 | -8,769 | | 1,98E-05 | |  |
| TOP3A | ENST00000580095.5 | | protein_coding | | tsl1 | -8,800 | | 1,69E-05 | |  |
| DEF8 | ENST00000567999.5 | | protein_coding | | tsl1 | -8,830 | | 1,74E-05 | |  |
| AKAP12 | ENST00000359755.5 | | protein_coding | | tsl1 | -8,832 | | 1,73E-05 | |  |
| CADM1 | ENST00000331581.10 | | protein_coding | | tsl1 | -8,859 | | 1,62E-05 | |  |
| HMGB3 | ENST00000430118.1 | | protein_coding | | tsl1 | -8,875 | | 1,55E-05 | |  |
| LPAR2 | ENST00000586703.1 | | protein_coding | | tsl1 | -8,876 | | 1,55E-05 | |  |
| RUNX1T1 | ENST00000360348.6 | | protein_coding | | tsl1 | -8,892 | | 7,07E-07 | |  |
| TMEM44 | ENST00000381975.7 | | protein_coding | | tsl1 | -8,903 | | 4,03E-06 | |  |
| RGS3 | ENST00000343817.9 | | protein_coding | | tsl1 | -8,909 | | 1,42E-05 | |  |
| RPS6KB1 | ENST00000406116.7 | | protein_coding | | tsl1 | -8,923 | | 1,37E-05 | |  |
| ADAMTS14 | ENST00000373207.1 | | protein_coding | | tsl1 | -8,962 | | 1,24E-05 | |  |
| ANTXR1 | ENST00000409349.7 | | protein_coding | | tsl1 | -8,964 | | 1,24E-05 | |  |
| USP21 | ENST00000368001.1 | | protein_coding | | tsl1 | -8,972 | | 1,22E-05 | |  |
| DDAH2 | ENST00000454138.6 | | protein_coding | | tsl1 | -8,976 | | 3,17E-06 | |  |
| DDAH2 | ENST00000434464.6 | | protein_coding | | tsl1 | -8,976 | | 3,17E-06 | |  |
| DDAH2 | ENST00000447101.6 | | protein_coding | | tsl1 | -8,976 | | 3,17E-06 | |  |
| NINL | ENST00000422516.5 | | protein_coding | | tsl1 | -9,006 | | 1,11E-05 | |  |
| STARD8 | ENST00000374599.7 | | protein_coding | | tsl1 | -9,024 | | 1,06E-05 | |  |
| HEXIM2 | ENST00000591576.5 | | protein_coding | | tsl1 | -9,032 | | 1,04E-05 | |  |
| CHEK2 | ENST00000382580.6 | | protein_coding | | tsl1 | -9,061 | | 9,69E-06 | |  |
| ANKZF1 | ENST00000409849.5 | | protein_coding | | tsl1 | -9,072 | | 9,41E-06 | |  |
| PIK3R1 | ENST00000336483.9 | | protein_coding | | tsl1 | -9,082 | | 9,19E-06 | |  |
| ARID1B | ENST00000637810.1 | | protein_coding | | tsl1 | -9,103 | | 8,71E-06 | |  |
| CNTRL | ENST00000238341.9 | | protein_coding | | tsl1 | -9,132 | | 8,08E-06 | |  |
| SMPD3 | ENST00000563226.1 | | protein_coding | | tsl1 | -9,142 | | 7,88E-06 | |  |
| CSRNP3 | ENST00000342316.8 | | protein_coding | | tsl1 | -9,162 | | 7,49E-06 | |  |
| SCAF4 | ENST00000434667.3 | | protein_coding | | tsl1 | -9,164 | | 7,44E-06 | |  |
| SPSB1 | ENST00000377399.2 | | protein_coding | | tsl1 | -9,166 | | 7,40E-06 | |  |
| PTPA | ENST00000347048.8 | | protein_coding | | tsl1 | -9,181 | | 6,94E-06 | |  |
| MCM8 | ENST00000265187.4 | | protein_coding | | tsl1 | -9,192 | | 6,93E-06 | |  |
| NEDD1 | ENST00000557644.5 | | protein_coding | | tsl1 | -9,213 | | 6,58E-06 | |  |
| TOP3A | ENST00000321105.9 | | protein_coding | | tsl1 | -9,239 | | 6,11E-06 | |  |
| SIRT1 | ENST00000403579.1 | | protein_coding | | tsl1 | -9,302 | | 5,24E-06 | |  |
| CENPL | ENST00000345664.10 | | protein_coding | | tsl1 | -9,314 | | 5,07E-06 | |  |
| C4A | ENST00000375295.8 | | protein_coding | | tsl1 | -9,327 | | 4,91E-06 | |  |
| TMBIM1 | ENST00000396809.6 | | protein_coding | | tsl1 | -9,329 | | 4,81E-06 | |  |
| CEP162 | ENST00000257766.8 | | protein_coding | | tsl1 | -9,334 | | 4,83E-06 | |  |
| SBNO1 | ENST00000420886.6 | | protein_coding | | tsl1 | -9,362 | | 3,79E-06 | |  |
| HNRNPK | ENST00000376263.7 | | protein_coding | | tsl1 | -9,394 | | 4,13E-06 | |  |
| AKNA | ENST00000374075.9 | | protein_coding | | tsl1 | -9,395 | | 4,13E-06 | |  |
| CYP2U1 | ENST00000508453.1 | | protein_coding | | tsl1 | -9,425 | | 3,82E-06 | |  |
| RUFY1 | ENST00000393438.6 | | protein_coding | | tsl1 | -9,445 | | 3,63E-06 | |  |
| ZNF419 | ENST00000424930.6 | | protein_coding | | tsl1 | -9,455 | | 3,47E-06 | |  |
| BAZ2A | ENST00000379441.7 | | protein_coding | | tsl1 | -9,490 | | 3,23E-06 | |  |
| ERMN | ENST00000410096.5 | | protein_coding | | tsl1 | -9,496 | | 3,17E-06 | |  |
| SH3BP2 | ENST00000356331.9 | | protein_coding | | tsl1 | -9,496 | | 3,17E-06 | |  |
| SGK3 | ENST00000396596.2 | | protein_coding | | tsl1 | -9,497 | | 3,15E-06 | |  |
| FAM192A | ENST00000389447.9 | | protein_coding | | tsl1 | -9,516 | | 3,02E-06 | |  |
| ZNF630 | ENST00000276054.8 | | protein_coding | | tsl1 | -9,556 | | 2,72E-06 | |  |
| DDX27 | ENST00000622530.4 | | protein_coding | | tsl1 | -9,664 | | 2,06E-06 | |  |
| PUF60 | ENST00000453551.6 | | protein_coding | | tsl1 | -9,665 | | 1,55E-06 | |  |
| SLC12A6 | ENST00000560611.5 | | protein_coding | | tsl1 | -9,733 | | 1,70E-06 | |  |
| BICRAL | ENST00000394168.1 | | protein_coding | | tsl1 | -9,755 | | 1,62E-06 | |  |
| COG2 | ENST00000366668.7 | | protein_coding | | tsl1 | -9,768 | | 1,57E-06 | |  |
| ABCF1 | ENST00000376545.7 | | protein_coding | | tsl1 | -9,781 | | 2,16E-07 | |  |
| MTRR | ENST00000264668.6 | | protein_coding | | tsl1 | -9,832 | | 1,33E-06 | |  |
| CRNKL1 | ENST00000377327.8 | | protein_coding | | tsl1 | -9,838 | | 1,20E-06 | |  |
| KIAA0586 | ENST00000423743.7 | | protein_coding | | tsl1 | -9,854 | | 1,26E-06 | |  |
| KIF27 | ENST00000334204.6 | | protein_coding | | tsl1 | -9,892 | | 1,14E-06 | |  |
| PTPA | ENST00000452489.6 | | protein_coding | | tsl1 | -9,894 | | 1,12E-06 | |  |
| LATS1 | ENST00000253339.9 | | protein_coding | | tsl1 | -9,908 | | 1,09E-06 | |  |
| ZSCAN25 | ENST00000334715.7 | | protein_coding | | tsl1 | -9,932 | | 5,03E-07 | |  |
| SOCS4 | ENST00000339298.2 | | protein_coding | | tsl1 | -9,992 | | 8,76E-07 | |  |
| EPS8L2 | ENST00000526198.5 | | protein_coding | | tsl1 | -10,014 | | 8,27E-07 | |  |
| SSBP1 | ENST00000571430.5 | | protein_coding | | tsl1 | -10,030 | | 7,93E-07 | |  |
| NFIB | ENST00000543693.5 | | protein_coding | | tsl1 | -10,057 | | 7,40E-07 | |  |
| ZNF569 | ENST00000392149.6 | | protein_coding | | tsl1 | -10,059 | | 7,37E-07 | |  |
| PUS3 | ENST00000227474.7 | | protein_coding | | tsl1 | -10,082 | | 6,56E-07 | |  |
| RGS11 | ENST00000397770.7 | | protein_coding | | tsl1 | -10,123 | | 6,22E-07 | |  |
| ATXN3 | ENST00000393287.9 | | protein_coding | | tsl1 | -10,207 | | 4,90E-07 | |  |
| TMEM63B | ENST00000371893.6 | | protein_coding | | tsl1 | -10,224 | | 4,77E-07 | |  |
| CEP128 | ENST00000281129.7 | | protein_coding | | tsl1 | -10,270 | | 4,23E-07 | |  |
| CTTN | ENST00000376561.7 | | protein_coding | | tsl1 | -10,346 | | 3,46E-07 | |  |
| MYH7B | ENST00000262873.11 | | protein_coding | | tsl1 | -10,389 | | 3,09E-07 | |  |
| NUP98 | ENST00000429801.5 | | protein_coding | | tsl1 | -10,407 | | 2,95E-07 | |  |
| MICB | ENST00000443156.2 | | protein_coding | | tsl1 | -10,571 | | 1,92E-07 | |  |
| ZFYVE1 | ENST00000318876.9 | | protein_coding | | tsl1 | -10,628 | | 1,65E-07 | |  |
| DENND2A | ENST00000537639.5 | | protein_coding | | tsl1 | -10,673 | | 1,47E-07 | |  |
| TNIP1 | ENST00000524280.5 | | protein_coding | | tsl1 | -10,776 | | 2,80E-08 | |  |
| ZBTB22 | ENST00000418724.1 | | protein_coding | | tsl1 | -10,837 | | 9,49E-08 | |  |
| ITPR1 | ENST00000456211.7 | | protein_coding | | tsl1 | -11,040 | | 5,54E-08 | |  |
| EHBP1 | ENST00000263991.9 | | protein_coding | | tsl1 | -11,205 | | 3,59E-08 | |  |
| RC3H1 | ENST00000258349.8 | | protein_coding | | tsl1 | -11,327 | | 7,65E-09 | |  |

**Supplemental Table S7. Complete list of significant (p-value ≤ 0,05) enriched canonical pathways of the differentially expressed genes using Ingenuity Pathway Analysis (IPA) software for insulin (INS) treatment.**

| Insulin Treatment |  |  |  |  |
| --- | --- | --- | --- | --- |
| Ingenuity canonical pathways | **- Log(p-value)** | **Ratio** | **Z-score** | **Molecules** |
| Calcium signaling | 7,98E00 | 2,12E-01 | -0,426 | ACTC1,ASPH,ATF2,ATP2A1,ATP2A3,ATP2B1,ATP2B4,ATP2C1,CABIN1,CACNA1C,CACNA2D1,CACNA2D3,CACNB2,CAMK2A,CAMK2B,CAMK2D,CHRFAM7A,CHRNA7,GRIA1,GRIA3,GRIA4,GRIK1,GRIN2A,ITPR1,MCU,MEF2C,MYH7B,NFATC2,NFATC3,PPP3CA,PRKAR1B,RCAN1,RCAN2,RCAN3,TNNI1,TNNT2,TPM1,TRPC1,TRPC3,TRPC4,TRPC6,TRPM8 |
| Neuropathic pain signaling in dorsal horn neurons | 5,29E00 | 2,19E-01 | -1,800 | ATM,CAMK2A,CAMK2B,CAMK2D,FGFR3,FRS2,GRIA1,GRIA3,GRIA4,GRIN2A,GRM8,ITPR1,NTRK2,PIK3C2B,PIK3CD,PIK3R1,PIK3R3,PLCB3,PLCB4,PLCE1,PLCH1,PLCL1,PRKAR1B,PRKCH,SRC |
| P53 signaling | 5,00 E00 | 2,16E-01 | 0,218 | ATM,BBC3,CDK2,CHEK2,COQ8A,FAS,FGFR3,FRS2,GADD45A,MAPK8,MDM4,PIDD1,PIK3C2B,PIK3CD,PIK3R1,PIK3R3,PLAGL1,PMAIP1,RRM2B,SERPINE2,SIRT1,STAG1,TNFRSF10A,TP73 |
| Signaling by rho family gtpases | 4,72E00 | 1,63E-01 | 0,324 | ACTC1,ACTR2,ARHGEF10,ARHGEF16,ARHGEF18,ARHGEF9,ATM,CDH10,CDH17,CDH23,CDH24,CDH7,FGFR3,FRS2,GNAO1,GNB4,GNB5,ITGA4,MAP3K21,MAPK10,MAPK12,MAPK8,NEDD4,NOX4,PAK6,PIK3C2B,PIK3CD,PIK3R1,PIK3R3,PIP5K1C,PLD1,PPP1R12A,PPP1R12B,PTK2,RDX,RELA,RHOT1,SEPT4,SEPT6,STMN1,WASF1 |
| Superpathway of inositol phosphate compounds | 4,62E00 | 1,67E-01 | 0,000 | ATM,CDC25A,DUSP14,EGFR,EPHX2,FGFR3,FRS2,INPP5J,IP6K3,MET,NUDT9,PIK3C2B,PIK3CD,PIK3R1,PIK3R3,PIP5K1C,PLCB3,PLCB4,PLCE1,PLCH1,PPFIA3,PPFIA4,PPFIBP2,PPM1K,PPP1R12A,PPP1R7,PPP2R3A,PPP2R5D,PPP3CA,PTPA,PTPN13,PTPN22,PTPRC,PXYLP1,SEC16A,STYX,SYNJ1,SYNJ2 |
| B cell receptor signaling | 4,56E00 | 1,76E-01 | -1,095 | ATF2,ATM,CAMK2A,CAMK2B,CAMK2D,FGFR3,FRS2,IKBKG,INPP5J,MAP3K6,MAP3K8,MAPK12,MAPK8,MEF2C,NFATC2,NFATC3,NFKBID,PAX5,PIK3C2B,PIK3CD,PIK3R1,PIK3R3,POU2F2,PPP3CA,PTK2,PTPRC,RASSF5,RELA,RPS6KB1,SYNJ1,SYNJ2,TCF3,VAV2 |
| Molecular mechanisms of cancer | 4,55E00 | 1,44E-01 | 0,000 | APH1B,ARHGEF10,ARHGEF16,ARHGEF18,ARHGEF9,ATM,BBC3,BID,CAMK2A,CAMK2B,CAMK2D,CASP8,CCNE1,CDC25A,CDK15,CDK2,CDK20,CHEK2,DAXX,DIABLO,E2F8,FAS,FGFR3,FRS2,GNAO1,IKBKG,ITGA4,LRP1,MAPK10,MAPK12,MAPK8,NAIP,NCSTN,NFKBID,PAK6,PIK3C2B,PIK3CD,PIK3R1,PIK3R3,PLCB3,PLCB4,PMAIP1,PRKAR1B,PRKCH,PTK2,RASGRP1,RBPJ,RELA,RHOT1,SMAD9,SRC,TCF3,WNT3,WNT5B,WNT8B,ZBTB17 |
| Gap junction signaling | 4,41E00 | 1,73E-01 | 0,000 | ACTC1,ATM,CSNK1E,CSNK1G1,EGFR,FGFR3,FRS2,GJB3,GRIA1,GRIA3,GRIA4,GRIK1,GUCY1A2,GUCY1B1,HTR2A,ITPR1,MAPK7,PIK3C2B,PIK3CD,PIK3R1,PIK3R3,PLCB3,PLCB4,PLCE1,PLCH1,PLCL1,PPP3CA,PRKAR1B,PRKCH,PRKG1,SRC,TJP2,TUBA8 |
| Calcium transport i | 4,31E00 | 0,6 | 0,000 | ATP2A1,ATP2A3,ATP2B1,ATP2B4,ATP2C1,ATP2C2 |
| Gα12/13 signaling | 3,98E00 | 1,85E-01 | -1,633 | ATM,CDH10,CDH17,CDH23,CDH24,CDH7,FGFR3,FRS2,IKBKG,LPAR2,MAPK10,MAPK12,MAPK7,MAPK8,MEF2C,NFKBID,PIK3C2B,PIK3CD,PIK3R1,PIK3R3,PTK2,PXN,RELA,SRC,VAV2 |
| Atm signaling | 3,96E00 | 2,06E-01 | 0,000 | ATF2,ATM,BID,CCNB3,CDC25A,CDK2,CHEK2,GADD45A,MAPK10,MAPK12,MAPK8,MDM4,PPP2R2B,PPP2R3A,PPP2R5D,PTPA,RAD50,RBBP8,TP73,ZEB1 |
| Creb signaling in neurons | 3,95E00 | 1,62E-01 | -1,961 | ATF2,ATM,CACNA1C,CACNA2D1,CACNA2D3,CACNB2,CAMK2A,CAMK2B,CAMK2D,FGFR3,FRS2,GNAO1,GNB4,GNB5,GRIA1,GRIA3,GRIA4,GRID2,GRIK1,GRIN2A,GRM8,ITPR1,PIK3C2B,PIK3CD,PIK3R1,PIK3R3,PLCB3,PLCB4,PLCE1,PLCH1,PLCL1,POLR2J2/POLR2J3,PRKAR1B,PRKCH |
| Nf-κb signaling | 3,87E00 | 1,69E-01 | -1,095 | ATM,CASP8,CSNK2B,EGFR,FGFR3,FRS2,IKBKG,IL18,IL1R1,IL37,IRAK1,MAP3K8,MAPK8,NFKBID,NTRK2,PIK3C2B,PIK3CD,PIK3R1,PIK3R3,RELA,TAB3,TGFA,TIRAP,TNFRSF11A,TNFRSF17,TNFRSF1B,TNFSF13B,TNIP1,UBE2V1,ZAP70 |
| Reelin signaling in neurons | 3,8E00 | 2,07E-01 | 0,000 | APP,ARHGEF10,ARHGEF16,ARHGEF9,ATM,FGFR3,FRS2,ITGA4,ITGAL,MAPK10,MAPK12,MAPK8,MAPT,PAFAH1B2,PIK3C2B,PIK3CD,PIK3R1,PIK3R3,SRC |
| Role of macrophages, fibroblasts and endothelial cells in rheumatoid arthritis | 3,77E00 | 1,45E-01 | 0,000 | APC2,ATF2,ATM,CAMK2A,CAMK2B,CAMK2D,CSF2,FGFR3,FN1,FRS2,GNAO1,IKBKG,IL18,IL1R1,IL1RAP,IL37,IRAK1,LRP1,NFATC2,NFATC3,NFKBID,NOS2,PIK3C2B,PIK3CD,PIK3R1,PIK3R3,PLCB3,PLCB4,PLCE1,PLCH1,PLCL1,PPP3CA,PRKCH,PRSS3,RELA,SRC,TCF3,TCF7L2,TNFRSF1B,TNFSF13B,VEGFA,WNT3,WNT5B,WNT8B |
| Role of nfat in cardiac hypertrophy | 3,71E00 | 1,57E-01 | -1,768 | ATM,CABIN1,CACNA1C,CACNA2D1,CACNA2D3,CACNB2,CAMK2A,CAMK2B,CAMK2D,FGFR3,FRS2,GNB4,GNB5,ITPR1,MAPK10,MAPK12,MAPK8,MEF2C,PIK3C2B,PIK3CD,PIK3R1,PIK3R3,PLCB3,PLCB4,PLCE1,PLCH1,PLCL1,PPP3CA,PRKAR1B,PRKCH,RCAN1,RCAN2,RCAN3,SRC |
| Thrombin signaling | 3,61E00 | 1,59E-01 | -0,928 | ARHGEF10,ARHGEF16,ARHGEF9,ATM,CAMK2A,CAMK2B,CAMK2D,EGFR,FGFR3,FRS2,GNAO1,GNB4,GNB5,ITPR1,MAPK12,PIK3C2B,PIK3CD,PIK3R1,PIK3R3,PLCB3,PLCB4,PLCE1,PLCH1,PLCL1,PPP1R12A,PPP1R12B,PRKCH,PTK2,RELA,RHOT1,RPS6KB1,SRC |
| Role of osteoblasts, osteoclasts and chondrocytes in rheumatoid arthritis | 3,58E00 | 1,54E-01 | 0,000 | ADAM17,APC2,ATM,CSF1R,CSF2,FGFR3,FRS2,IKBKG,IL18,IL1R1,IL1RAP,IL37,LRP1,MAPK10,MAPK12,MAPK8,NAIP,NFATC2,NFATC3,NFKBID,PIK3C2B,PIK3CD,PIK3R1,PIK3R3,PPP3CA,RELA,SMAD9,SRC,TCF3,TCF7L2,TNFRSF11A,TNFRSF1B,WNT3,WNT5B,WNT8B |
| Induction of apoptosis by hiv1 | 3,52E00 | 2,33E-01 | 0,000 | BBC3,BID,CASP8,DAXX,DIABLO,FAS,IKBKG,MAPK10,MAPK12,MAPK8,NAIP,NFKBID,RELA,TNFRSF1B |
| Breast cancer regulation by stathmin1 | 3,5E00 | 1,57E-01 | 0,000 | ARHGEF10,ARHGEF16,ARHGEF18,ARHGEF9,ATM,CAMK2A,CAMK2B,CAMK2D,CCNE1,CDK2,E2F8,FGFR3,FRS2,GNB4,GNB5,ITPR1,PIK3C2B,PIK3CD,PIK3R1,PIK3R3,PLCB3,PLCB4,PPP1R12A,PPP1R7,PPP2R2B,PPP2R3A,PPP2R5D,PRKAR1B,PRKCH,PTPA,STMN1,TUBA8 |
| Pkcθ signaling in t lymphocytes | 3,45E00 | 1,69E-01 | -0,426 | ATM,CACNA1C,CACNA2D1,CACNA2D3,CACNB2,CAMK2A,CAMK2B,CAMK2D,FGFR3,FRS2,IKBKG,LAT,MAP3K6,MAP3K8,MAPK8,NFATC2,NFATC3,NFKBID,PIK3C2B,PIK3CD,PIK3R1,PIK3R3,PPP3CA,RELA,VAV2,ZAP70 |
| Egf signaling | 3,45E00 | 2,21E-01 | -1,291 | ATM,CSNK2B,EGFR,FGFR3,FRS2,ITPR1,MAPK12,MAPK8,PIK3C2B,PIK3CD,PIK3R1,PIK3R3,RPS6KB1,SRC,STAT1 |
| Ctla4 signaling in cytotoxic t lymphocytes | 3,43E00 | 1,94E-01 | 0,000 | AP1B1,AP1G2,AP1S3,AP2B1,ATM,CD8A,FGFR3,FRS2,LAT,PIK3C2B,PIK3CD,PIK3R1,PIK3R3,PPP2R2B,PPP2R3A,PPP2R5D,PTPA,PTPN22,ZAP70 |
| Role of nfat in regulation of the immune response | 3,38E00 | 1,6E-01 | -1,043 | ATF2,ATM,CABIN1,CSNK1E,CSNK1G1,FGFR3,FRS2,GNAO1,GNB4,GNB5,IKBKG,ITPR1,LAT,MEF2C,NFATC2,NFATC3,NFKBID,PIK3C2B,PIK3CD,PIK3R1,PIK3R3,PLCB3,PLCB4,PPP3CA,RCAN1,RCAN2,RCAN3,RELA,ZAP70 |
| Role of il-17a in arthritis | 3,38E00 | 2,17E-01 | 0,000 | ATF2,ATM,FGFR3,FRS2,IKBKG,MAPK10,MAPK12,MAPK8,NFKBID,NOS2,PIK3C2B,PIK3CD,PIK3R1,PIK3R3,RELA |
| Angiopoietin signaling | 3,33E00 | 2,08E-01 | 1,414 | ATM,FGFR3,FRS2,GRB14,GRB7,IKBKG,NFKBID,PAK6,PIK3C2B,PIK3CD,PIK3R1,PIK3R3,PTK2,RELA,TIE1,TNIP1 |
| Rank signaling in osteoclasts | 3,32E00 | 1,9E-01 | -0,728 | ATM,FGFR3,FRS2,IKBKG,MAP3K6,MAP3K8,MAPK10,MAPK12,MAPK8,NFATC2,NFKBID,PIK3C2B,PIK3CD,PIK3R1,PIK3R3,PPP3CA,RELA,SRC,TNFRSF11A |
| Ceramide signaling | 3,27E00 | 1,94E-01 | 0,000 | ATM,CNKSR1,DIABLO,FGFR3,FRS2,MAPK8,PIK3C2B,PIK3CD,PIK3R1,PIK3R3,PPP2R2B,PPP2R3A,PPP2R5D,PTPA,RELA,SMPD3,SPHK1,TNFRSF1B |
| Rhogdi signaling | 3,23E00 | 1,59E-01 | -1,400 | ACTC1,ACTR2,ARHGAP12,ARHGAP4,ARHGAP6,ARHGAP9,ARHGDIA,ARHGEF10,ARHGEF16,ARHGEF18,ARHGEF9,CDH10,CDH17,CDH23,CDH24,CDH7,GNAO1,GNB4,GNB5,ITGA4,PAK6,PIP5K1C,PPP1R12A,PPP1R12B,RDX,RHOT1,SRC,WASF1 |
| Il-4 signaling | 3,23E00 | 1,98E-01 | 0,000 | ATM,FGFR3,FRS2,IL13RA1,INPP5J,NFATC2,NFATC3,NR3C1,NR3C2,PIK3C2B,PIK3CD,PIK3R1,PIK3R3,RPS6KB1,STAT6,SYNJ1,SYNJ2 |
| Axonal guidance signaling | 3,22E00 | 1,28E-01 | 0,000 | ABLIM2,ABLIM3,ACTR2,ADAM17,ADAM22,ADAM8,ADAMTS9,AOPEP,ATM,ENPEP,EPHA3,EPHA5,EPHB6,FES,FGFR3,FRS2,GNAO1,GNB4,GNB5,ITGA4,LRRC4C,MET,MME,NFATC2,NFATC3,NTN4,NTRK2,PAK6,PIK3C2B,PIK3CD,PIK3R1,PIK3R3,PLCB3,PLCB4,PLCE1,PLCH1,PLCL1,PPP3CA,PRKAR1B,PRKCH,PTK2,PXN,RASSF5,RGS3,RTN4,SEMA3F,SEMA4D,SEMA6A,SEMA6B,SRGAP2,SRGAP3,TUBA8,UNC5C,VEGFA,WNT3,WNT5B,WNT8B |
| 3-phosphoinositide biosynthesis | 3,21E00 | 1,55E-01 | 0,000 | ATM,CDC25A,DUSP14,EGFR,EPHX2,FGFR3,FRS2,MET,NUDT9,PIK3C2B,PIK3CD,PIK3R1,PIK3R3,PIP5K1C,PPFIA3,PPFIA4,PPFIBP2,PPM1K,PPP1R12A,PPP1R7,PPP2R3A,PPP2R5D,PPP3CA,PTPA,PTPN13,PTPN22,PTPRC,PXYLP1,STYX,SYNJ1 |
| Synaptic long term depression | 3,21E00 | 1,61E-01 | -1,732 | CACNA1C,CACNA2D1,CACNA2D3,CACNB2,GNAO1,GRIA1,GRIA3,GRIA4,GRID2,GRM8,GUCY1A2,GUCY1B1,ITPR1,NOS2,PAFAH1B2,PLA2G1B,PLCB3,PLCB4,PLCE1,PLCH1,PLCL1,PPP2R2B,PPP2R3A,PPP2R5D,PRKCH,PRKG1,PTPA |
| Tec kinase signaling | 3,21E00 | 1,61E-01 | -0,209 | ACTC1,ATM,FAS,FGFR3,FRS2,GNAO1,GNB4,GNB5,ITGA4,MAPK10,MAPK12,MAPK8,PAK6,PIK3C2B,PIK3CD,PIK3R1,PIK3R3,PRKCH,PTK2,RELA,RHOT1,SRC,STAT1,STAT6,TNFRSF10A,TNFSF10,VAV2 |
| Amyotrophic lateral sclerosis signaling | 3,2E00 | 1,82E-01 | 0,000 | ATM,BID,CACNA1C,CAPN3,CAPN8,FGFR3,FRS2,GRIA1,GRIA3,GRIA4,GRID2,GRIK1,GRIN2A,NAIP,PIK3C2B,PIK3CD,PIK3R1,PIK3R3,PPP3CA,VEGFA |
| 3-phosphoinositide degradation | 3,17E00 | 1,64E-01 | 0,000 | CDC25A,DUSP14,EPHX2,INPP4B,INPP5J,MTMR1,MTMR3,NUDT9,PPFIA3,PPFIA4,PPFIBP2,PPM1K,PPP1R12A,PPP1R7,PPP2R3A,PPP2R5D,PPP3CA,PTPA,PTPN13,PTPN22,PTPRC,PXYLP1,STYX,SYNJ1,SYNJ2 |
| Synaptic long term potentiation | 3,16E00 | 1,76E-01 | -2,683 | ATF2,CACNA1C,CAMK2A,CAMK2B,CAMK2D,GRIA1,GRIA3,GRIA4,GRIN2A,GRM8,ITPR1,PLCB3,PLCB4,PLCE1,PLCH1,PLCL1,PPP1R12A,PPP1R7,PPP3CA,PRKAR1B,PRKCH |
| Leukocyte extravasation signaling | 3,09E00 | 1,5E-01 | -1,347 | ACTC1,AFDN,ARHGAP12,ARHGAP4,ARHGAP6,ARHGAP9,ATM,CLDN15,CLDN2,CLDN7,CTTN,FGFR3,FRS2,ICAM3,ITGA4,ITGAL,MAPK10,MAPK12,MAPK8,PIK3C2B,PIK3CD,PIK3R1,PIK3R3,PRKCH,PTK2,PXN,RASGRP1,RASSF5,RDX,SRC,VAV2 |
| Uva-induced mapk signaling | 3,05E00 | 1,81E-01 | -0,832 | ATM,EGFR,FGFR3,FRS2,MAPK10,MAPK12,MAPK8,PIK3C2B,PIK3CD,PIK3R1,PIK3R3,PLCB3,PLCB4,PLCE1,PLCH1,PLCL1,RPS6KB1,SMPD3,STAT1 |
| Fmlp signaling in neutrophils | 3,01E00 | 1,72E-01 | -0,229 | ACTR2,ATM,FGFR3,FRS2,GNB4,GNB5,IKBKG,ITPR1,NFATC2,NFATC3,NFKBID,NOX4,PIK3C2B,PIK3CD,PIK3R1,PIK3R3,PLCB3,PLCB4,PPP3CA,PRKCH,RELA |
| P70s6k signaling | 2,98E00 | 1,68E-01 | -1,706 | ATM,EGFR,FGFR3,FRS2,MAPT,PIK3C2B,PIK3CD,PIK3R1,PIK3R3,PLCB3,PLCB4,PLCE1,PLCH1,PLCL1,PLD1,PPP2R2B,PPP2R3A,PPP2R5D,PRKCH,PTPA,RPS6KB1,SRC |
| T cell receptor signaling | 2,89E00 | 1,76E-01 | 0,000 | ATM,CD8A,FGFR3,FRS2,IKBKG,LAT,MAPK8,NFATC2,NFATC3,PIK3C2B,PIK3CD,PIK3R1,PIK3R3,PPP3CA,PTPRC,RASGRP1,RELA,VAV2,ZAP70 |
| Gαq signaling | 2,88E00 | 1,57E-01 | -0,853 | ARHGEF25,ATM,FGFR3,FRS2,GNB4,GNB5,GYS1,HTR2A,IKBKG,ITPR1,NFATC2,NFATC3,NFKBID,PIK3C2B,PIK3CD,PIK3R1,PIK3R3,PLCB3,PLCB4,PLD1,PPP3CA,PRKCH,RELA,RGS7,RHOT1 |
| Dopamine-darpp32 feedback in camp signaling | 2,84E00 | 1,56E-01 | -1,460 | ATF2,ATP2A1,ATP2A3,CACNA1C,CSNK1E,CSNK1G1,GRIN2A,GUCY1A2,GUCY1B1,ITPR1,PLCB3,PLCB4,PLCE1,PLCH1,PLCL1,PPP1R12A,PPP1R7,PPP2R2B,PPP2R3A,PPP2R5D,PPP3CA,PRKAR1B,PRKCH,PRKG1,PTPA |
| B cell activating factor signaling | 2,83E00 | 2,44E-01 | 0,333 | IKBKG,MAPK10,MAPK12,MAPK8,NFATC2,NFATC3,NFKBID,RELA,TNFRSF17,TNFSF13B |
| Icos-icosl signaling in t helper cells | 2,81E00 | 1,69E-01 | 0,000 | ATM,CAMK2A,CAMK2B,CAMK2D,FGFR3,FRS2,IKBKG,ITPR1,LAT,NFATC2,NFATC3,NFKBID,PIK3C2B,PIK3CD,PIK3R1,PIK3R3,PPP3CA,PTPRC,RELA,ZAP70 |
| Cd28 signaling in t helper cells | 2,78E00 | 1,65E-01 | -0,447 | ACTR2,ATM,FGFR3,FRS2,IKBKG,ITPR1,LAT,MAPK10,MAPK12,MAPK8,NFATC2,NFATC3,NFKBID,PIK3C2B,PIK3CD,PIK3R1,PIK3R3,PPP3CA,PTPRC,RELA,ZAP70 |
| Gnrh signaling | 2,76E00 | 1,54E-01 | -0,655 | ATF2,CACNA1C,CACNA2D1,CACNA2D3,CACNB2,CAMK2A,CAMK2B,CAMK2D,EGFR,ITPR1,MAP3K6,MAP3K8,MAPK10,MAPK12,MAPK7,MAPK8,PAK6,PLCB3,PLCB4,PRKAR1B,PRKCH,PTK2,PXN,RELA,SRC |
| Il-6 signaling | 2,74E00 | 1,64E-01 | -0,655 | ATM,CSNK2B,FGFR3,FRS2,HSPB7,IKBKG,IL18,IL1R1,IL1RAP,IL37,MAPK10,MAPK12,MAPK8,NFKBID,PIK3C2B,PIK3CD,PIK3R1,PIK3R3,RELA,TNFRSF1B,VEGFA |
| Pedf signaling | 2,73E00 | 1,84E-01 | -0,258 | ATM,CASP8,FAS,FGFR3,FRS2,IKBKG,MAPK12,NFKBID,PIK3C2B,PIK3CD,PIK3R1,PIK3R3,PPARG,RELA,TCF7L2,ZEB1 |
| Pancreatic adenocarcinoma signaling | 2,72E00 | 1,67E-01 | -0,243 | ATM,CCNE1,CDK2,CYP2E1,E2F8,EGFR,FGFR3,FRS2,MAPK10,MAPK12,MAPK8,PIK3C2B,PIK3CD,PIK3R1,PIK3R3,PLD1,RELA,STAT1,TGFA,VEGFA |
| Phenylethylamine degradation i | 2,68E00 | 7,5E-01 | 0,000 | ALDH3A2,AOC2,AOC3 |
| Amyloid processing | 2,67E00 | 2,2E-01 | -0,447 | APH1B,APP,CAPN3,CAPN8,CSNK1E,CSNK2B,MAPK12,MAPT,MARK1,NCSTN,PRKAR1B |
| D-myo-inositol-5-phosphate metabolism | 2,66E00 | 1,54E-01 | 0,000 | CDC25A,DUSP14,EPHX2,NUDT9,PLCB3,PLCB4,PLCE1,PLCH1,PPFIA3,PPFIA4,PPFIBP2,PPM1K,PPP1R12A,PPP1R7,PPP2R3A,PPP2R5D,PPP3CA,PTPA,PTPN13,PTPN22,PTPRC,PXYLP1,STYX,SYNJ1 |
| Gm-csf signaling | 2,63E00 | 1,92E-01 | -0,577 | ATM,CAMK2A,CAMK2B,CAMK2D,CSF2,CSF2RA,FGFR3,FRS2,PIK3C2B,PIK3CD,PIK3R1,PIK3R3,PPP3CA,STAT1 |
| Hgf signaling | 2,61E00 | 1,67E-01 | -0,688 | ATF2,ATM,CDK2,FGFR3,FRS2,ITGA4,MAP3K6,MAP3K8,MAPK10,MAPK12,MAPK8,MET,PIK3C2B,PIK3CD,PIK3R1,PIK3R3,PRKCH,PTK2,PXN |
| Ilk signaling | 2,61E00 | 1,45E-01 | -1,633 | ACTC1,ATF2,ATM,FERMT2,FGFR3,FN1,FRS2,LIMS1,LIMS2,MAPK10,MAPK12,MAPK8,MYH7B,NOS2,PIK3C2B,PIK3CD,PIK3R1,PIK3R3,PPP1R12A,PPP2R2B,PPP2R3A,PPP2R5D,PTK2,PTPA,PXN,RELA,RHOT1,VEGFA |
| Uvb-induced mapk signaling | 2,59E00 | 1,97E-01 | -0,277 | ATM,EGFR,FGFR3,FRS2,MAPK10,MAPK12,MAPK8,PIK3C2B,PIK3CD,PIK3R1,PIK3R3,PRKCH,RPS6KB1 |
| Pdgf signaling | 2,57E00 | 1,78E-01 | -1,000 | ABL2,ATM,CSNK2B,FGFR3,FRS2,INPP5J,MAPK8,PIK3C2B,PIK3CD,PIK3R1,PIK3R3,SPHK1,SRC,STAT1,SYNJ1,SYNJ2 |
| Type i diabetes mellitus signaling | 2,55E00 | 1,68E-01 | 0,000 | BID,CASP8,FAS,ICA1,IKBKG,IL1R1,IL1RAP,IRAK1,MAPK10,MAPK12,MAPK8,NFKBID,NOS2,RELA,SOCS4,SOCS7,STAT1,TNFRSF1B |
| Endothelin-1 signaling | 2,54E00 | 1,45E-01 | -2,502 | ATM,CASP8,FGFR3,FRS2,GNAO1,GUCY1A2,GUCY1B1,ITPR1,MAPK10,MAPK12,MAPK7,MAPK8,NOS2,PAFAH1B2,PIK3C2B,PIK3CD,PIK3R1,PIK3R3,PLA2G1B,PLCB3,PLCB4,PLCE1,PLCH1,PLCL1,PLD1,PRKCH,SRC |
| Agrin interactions at neuromuscular junction | 2,53E00 | 1,94E-01 | -0,832 | ACTC1,CTTN,EGFR,GABPB1,ITGA4,ITGAL,MAPK10,MAPK12,MAPK8,PAK6,PTK2,PXN,SRC |
| Nitric oxide signaling in the cardiovascular system | 2,5E00 | 1,67E-01 | 0,471 | ATM,ATP2A1,ATP2A3,CACNA1C,FGFR3,FRS2,GUCY1A2,GUCY1B1,HSP90B1,ITPR1,PIK3C2B,PIK3CD,PIK3R1,PIK3R3,PRKAR1B,PRKCH,PRKG1,VEGFA |
| Type ii diabetes mellitus signaling | 2,48E00 | 1,51E-01 | -1,000 | ATM,CACNA1C,CACNA2D1,CACNA2D3,CACNB2,FGFR3,FRS2,IKBKG,MAPK10,MAPK12,MAPK8,NFKBID,PIK3C2B,PIK3CD,PIK3R1,PIK3R3,PPARG,PRKCH,RELA,SMPD3,SOCS4,SOCS7,TNFRSF1B |
| Death receptor signaling | 2,47E00 | 1,74E-01 | -0,500 | ACTC1,BID,CASP8,DAXX,DIABLO,FAS,GAS2,HSPB7,IKBKG,MAPK8,NAIP,NFKBID,RELA,TNFRSF10A,TNFRSF1B,TNFSF10 |
| Glioblastoma multiforme signaling | 2,47E00 | 1,49E-01 | -1,279 | ATM,CCNE1,CDK2,E2F8,EGFR,FGFR3,FRS2,ITPR1,PIK3C2B,PIK3CD,PIK3R1,PIK3R3,PLCB3,PLCB4,PLCE1,PLCH1,PLCL1,RHOT1,RPS6KB1,SRC,TCF3,WNT3,WNT5B,WNT8B |
| Pi3k signaling in b lymphocytes | 2,46E00 | 1,59E-01 | -1,789 | ATF2,CAMK2A,CAMK2B,CAMK2D,IKBKG,ITPR1,NFATC2,NFATC3,NFKBID,PIK3CD,PIK3R1,PLCB3,PLCB4,PLCE1,PLCH1,PLCL1,PPP3CA,PTPRC,RELA,VAV2 |
| Xenobiotic metabolism signaling | 2,46E00 | 1,32E-01 | 0,000 | ALDH3A2,ALDH4A1,ATM,CAMK2A,CAMK2B,CAMK2D,CES1,FGFR3,FRS2,GAL3ST2,GSTM1,HS3ST4,HSP90B1,MAP3K6,MAP3K8,MAPK12,MAPK7,MAPK8,NCOR2,NOS2,NRIP1,PIK3C2B,PIK3CD,PIK3R1,PIK3R3,PPARGC1A,PPP2R2B,PPP2R3A,PPP2R5D,PRKCH,PTPA,RELA,SULT1A1,SULT1C2,SULT4A1,UGT1A7 (includes others) |
| Cholecystokinin/gastrin-mediated signaling | 2,44E00 | 1,68E-01 | -1,213 | ATF2,EGFR,IL18,IL37,ITPR1,MAPK10,MAPK12,MAPK7,MAPK8,MEF2C,PLCB3,PLCB4,PRKCH,PTK2,PXN,RHOT1,SRC |
| April mediated signaling | 2,42E00 | 2,31E-01 | 1,000 | IKBKG,MAPK10,MAPK12,MAPK8,NFATC2,NFATC3,NFKBID,RELA,TNFRSF17 |
| Paxillin signaling | 2,42E00 | 1,64E-01 | -1,500 | ACTC1,ATM,FGFR3,FRS2,ITGA10,ITGA4,ITGAL,MAPK10,MAPK12,MAPK8,PAK6,PIK3C2B,PIK3CD,PIK3R1,PIK3R3,PTK2,PXN,SRC |
| Small cell lung cancer signaling | 2,42E00 | 1,76E-01 | -0,577 | ATM,BID,CCNE1,CDK2,FGFR3,FRS2,IKBKG,NFKBID,PIK3C2B,PIK3CD,PIK3R1,PIK3R3,PTK2,RELA,RXRB |
| Leptin signaling in obesity | 2,42E00 | 1,76E-01 | 0,000 | ATM,FGFR3,FRS2,GHRL,LEPR,PIK3C2B,PIK3CD,PIK3R1,PIK3R3,PLCB3,PLCB4,PLCE1,PLCH1,PLCL1,PRKAR1B |
| Dna double-strand break repair by homologous recombination | 2,39E00 | 3,57E-01 | 0,000 | ATM,ATRX,LIG1,POLA1,RAD50 |
| Dna double-strand break repair by non-homologous end joining | 2,39E00 | 3,57E-01 | 0,000 | ATM,DCLRE1C,LIG3,RAD50,XRCC4 |
| Protein kinase a signaling | 2,37E00 | 1,22E-01 | -2,058 | AKAP12,AKAP14,AKAP6,AKAP7,ATF2,CAMK2A,CAMK2B,CAMK2D,CDC16,CDC25A,CDKN3,EPM2A,EYA3,GNB4,GNB5,GYS1,IKBKG,ITPR1,MTMR3,NFATC2,NFATC3,NFKBID,PDE4A,PLCB3,PLCB4,PLCE1,PLCH1,PLCL1,PPP1R12A,PPP1R7,PPP3CA,PRKAR1B,PRKCH,PTK2,PTPDC1,PTPN13,PTPN22,PTPRB,PTPRC,PTPRU,PXN,PYGL,RELA,TCF3,TCF7L2,TH,TNNI1 |
| Sertoli cell-sertoli cell junction signaling | 2,37E00 | 1,45E-01 | 0,000 | ACTC1,AFDN,ATF2,CLDN15,CLDN2,CLDN7,GUCY1A2,GUCY1B1,ITGA4,MAGI2,MAP3K6,MAP3K8,MAPK10,MAPK12,MAPK8,NECTIN3,NOS2,PLS1,PRKAR1B,PRKG1,SORBS1,SPTB,SRC,TJP2,TUBA8 |
| Hippo signaling | 2,37E00 | 1,74E-01 | 0,000 | AMOT,CSNK1E,DLG2,DLG4,LATS1,PPP1R12A,PPP1R7,PPP2R2B,PPP2R3A,PPP2R5D,PTPA,SCRIB,STK4,TEAD2,TJP2 |
| Wnt/ca+ pathway | 2,37E00 | 1,94E-01 | -1,155 | ATF2,CAMK2A,NFATC2,NFATC3,PLCB3,PLCB4,PLCE1,PLCH1,PLCL1,PPP3CA,RELA,WNT5B |
| Myc mediated apoptosis signaling | 2,36E00 | 1,86E-01 | 0,000 | ATM,BID,CASP8,FAS,FGFR3,FRS2,MAPK10,MAPK12,MAPK8,PIK3C2B,PIK3CD,PIK3R1,PIK3R3 |
| Production of nitric oxide and reactive oxygen species in macrophages | 2,32E00 | 1,4E-01 | -0,192 | APOC1,ATM,FGFR3,FRS2,IKBKG,MAP3K6,MAP3K8,MAPK10,MAPK12,MAPK8,NFKBID,NOS2,PIK3C2B,PIK3CD,PIK3R1,PIK3R3,PPP1R12A,PPP1R7,PPP2R2B,PPP2R3A,PPP2R5D,PRKCH,PTPA,RELA,RHOT1,STAT1,TNFRSF1B |
| Lps-stimulated mapk signaling | 2,32E00 | 1,72E-01 | -0,775 | ATF2,ATM,FGFR3,FRS2,IKBKG,MAPK10,MAPK12,MAPK8,NFKBID,PIK3C2B,PIK3CD,PIK3R1,PIK3R3,PRKCH,RELA |
| 14-3-3-mediated signaling | 2,31E00 | 1,54E-01 | -1,414 | ATM,FGFR3,FRS2,MAPK10,MAPK12,MAPK8,MAPT,PIK3C2B,PIK3CD,PIK3R1,PIK3R3,PLCB3,PLCB4,PLCE1,PLCH1,PLCL1,PRKCH,SRC,TP73,TUBA8 |
| Fcγriib signaling in b lymphocytes | 2,31E00 | 1,77E-01 | -0,632 | ATM,CACNA1C,CACNA2D1,CACNA2D3,CACNB2,FGFR3,FRS2,MAPK10,MAPK12,MAPK8,PIK3C2B,PIK3CD,PIK3R1,PIK3R3 |
| Dendritic cell maturation | 2,28E00 | 1,41E-01 | -2,353 | ATF2,ATM,COL10A1,CSF2,FGFR3,FRS2,IKBKG,IL18,IL37,LEPR,MAPK10,MAPK12,MAPK8,NFKBID,PIK3C2B,PIK3CD,PIK3R1,PIK3R3,PLCB3,PLCB4,PLCE1,PLCH1,PLCL1,RELA,STAT1,TNFRSF1B |
| Rhoa signaling | 2,28E00 | 1,56E-01 | -1,213 | ABL2,ACTC1,ACTR2,ARHGAP12,ARHGAP4,ARHGAP6,ARHGAP9,LPAR2,NEDD4,PIP5K1C,PLD1,PPP1R12A,PPP1R12B,PTK2,RDX,SEMA3F,SEPT4,SEPT6,WASF1 |
| Aldosterone signaling in epithelial cells | 2,27E00 | 1,44E-01 | -1,886 | ATM,DNAJB5,DNAJC22,FGFR3,FRS2,HSP90B1,HSPA8,HSPB7,ITPR1,NEDD4,NR3C2,PIK3C2B,PIK3CD,PIK3R1,PIK3R3,PIP5K1C,PLCB3,PLCB4,PLCE1,PLCH1,PLCL1,PRKCH,SACS,SGK1 |
| Pyridoxal 5'-phosphate salvage pathway | 2,25E00 | 1,88E-01 | 0,000 | CDK2,DAPK1,DMPK,IRAK1,MAK,MAP3K6,MAP3K8,MAPK7,MAPK8,NEK2,PRKCH,SGK1 |
| Pi3k/akt signaling | 2,24E00 | 1,54E-01 | 0,000 | GYS1,HSP90B1,IKBKG,INPP5J,ITGA4,LIMS1,MAP3K8,NFKBID,PIK3CD,PIK3R1,PIK3R3,PPP2R2B,PPP2R3A,PPP2R5D,PTPA,RELA,RPS6KB1,SYNJ1,SYNJ2 |
| Igf-1 signaling | 2,22E00 | 1,6E-01 | -1,500 | ATM,CSNK2B,FGFR3,FRS2,GRB10,MAPK8,NEDD4,PIK3C2B,PIK3CD,PIK3R1,PIK3R3,PRKAR1B,PTK2,PXN,RPS6KB1,SOCS4,SOCS7 |
| Role of chk proteins in cell cycle checkpoint control | 2,2E00 | 1,93E-01 | -0,333 | ATM,CDC25A,CDK2,CHEK2,CLSPN,E2F8,PPP2R2B,PPP2R3A,PPP2R5D,PTPA,RAD50 |
| Fak signaling | 2,2E00 | 1,63E-01 | 0,000 | ACTC1,ATM,CAPN3,CAPN8,EGFR,FGFR3,FRS2,ITGA4,PAK6,PIK3C2B,PIK3CD,PIK3R1,PIK3R3,PTK2,PXN,SRC |
| Netrin signaling | 2,2E00 | 1,85E-01 | 0,000 | ABLIM2,ABLIM3,CACNA1C,CACNA2D1,CACNA2D3,CACNB2,NFATC2,NFATC3,PPP3CA,PRKAR1B,PRKG1,UNC5C |
| Rac signaling | 2,17E00 | 1,55E-01 | -1,414 | ACTR2,ATM,FGFR3,FRS2,ITGA4,MAPK8,NOX4,PAK6,PIK3C2B,PIK3CD,PIK3R1,PIK3R3,PIP5K1C,PLD1,PTK2,RELA,RPS6KB1,WASF1 |
| Acute myeloid leukemia signaling | 2,09E00 | 1,63E-01 | -0,832 | ATM,CSF1R,CSF2RA,FGFR3,FRS2,IDH3B,IDH3G,PIK3C2B,PIK3CD,PIK3R1,PIK3R3,RELA,RPS6KB1,TCF3,TCF7L2 |
| Fc epsilon ri signaling | 2,06E00 | 1,51E-01 | -0,243 | ATM,CSF2,FGFR3,FRS2,INPP5J,LAT,MAPK10,MAPK12,MAPK8,PIK3C2B,PIK3CD,PIK3R1,PIK3R3,PLA2G1B,PRKCH,SYNJ1,SYNJ2,VAV2 |
| Pten signaling | 2,06E00 | 1,51E-01 | 0,500 | CSNK2B,EGFR,FGFR3,IKBKG,INPP5J,ITGA4,MAGI2,MCRS1,NTRK2,PIK3CD,PIK3R1,PIK3R3,PTK2,RELA,RPS6KB1,SYNJ1,SYNJ2,TNFRSF11A |
| Cd27 signaling in lymphocytes | 2,04E00 | 1,92E-01 | 0,333 | BID,CASP8,IKBKG,MAP3K6,MAP3K8,MAPK10,MAPK12,MAPK8,NFKBID,RELA |
| Virus entry via endocytic pathways | 2,03E00 | 1,57E-01 | 0,000 | ACTC1,AP1B1,AP1G2,AP2B1,ATM,CXADR,FGFR3,FRS2,ITGA4,ITGAL,PIK3C2B,PIK3CD,PIK3R1,PIK3R3,PRKCH,SRC |
| Ephrin a signaling | 2,03E00 | 1,83E-01 | 0,000 | ATM,EPHA3,EPHA5,FGFR3,FRS2,PIK3C2B,PIK3CD,PIK3R1,PIK3R3,PTK2,VAV2 |
| D-myo-inositol (1,4,5,6)-tetrakisphosphate biosynthesis | 2,02E00 | 1,45E-01 | 0,000 | CDC25A,DUSP14,EPHX2,NUDT9,PPFIA3,PPFIA4,PPFIBP2,PPM1K,PPP1R12A,PPP1R7,PPP2R3A,PPP2R5D,PPP3CA,PTPA,PTPN13,PTPN22,PTPRC,PXYLP1,STYX,SYNJ1 |
| D-myo-inositol (3,4,5,6)-tetrakisphosphate biosynthesis | 2,02E00 | 1,45E-01 | 0,000 | CDC25A,DUSP14,EPHX2,NUDT9,PPFIA3,PPFIA4,PPFIBP2,PPM1K,PPP1R12A,PPP1R7,PPP2R3A,PPP2R5D,PPP3CA,PTPA,PTPN13,PTPN22,PTPRC,PXYLP1,STYX,SYNJ1 |
| Ngf signaling | 2,02E00 | 1,5E-01 | -0,471 | ATF2,ATM,FGFR3,FRS2,IKBKG,MAP3K6,MAP3K8,MAPK10,MAPK12,MAPK7,MAPK8,PIK3C2B,PIK3CD,PIK3R1,PIK3R3,RELA,RPS6KB1,SMPD3 |
| Neuroinflammation signaling pathway | 2,02E00 | 1,23E-01 | -0,667 | ACVR1,APH1B,APP,ATF2,ATM,CASP8,CSF1R,FAS,FGFR3,FRS2,GABBR1,GRIA1,GRIN2A,IKBKG,IL18,IL1R1,IL34,IRAK1,MAPK10,MAPK12,MAPK7,MAPK8,MAPT,NCSTN,NFATC2,NFATC3,NOS2,NOX4,PIK3C2B,PIK3CD,PIK3R1,PIK3R3,PLA2G1B,PPP3CA,RELA,STAT1,TIRAP |
| Non-small cell lung cancer signaling | 2 | 1,69E-01 | -0,302 | ATM,EGFR,FGFR3,FRS2,ITPR1,PIK3C2B,PIK3CD,PIK3R1,PIK3R3,RASSF5,RXRB,STK4,TGFA |
| Il-9 signaling | 1,99E00 | 0,2 | 0,333 | ATM,FGFR3,FRS2,PIK3C2B,PIK3CD,PIK3R1,PIK3R3,RELA,STAT1 |
| Sapk/jnk signaling | 1,99E00 | 1,55E-01 | -1,500 | ATF2,ATM,DAXX,FGFR3,FRS2,GADD45A,HNRNPK,MAPK10,MAPK12,MAPK8,MINK1,NFATC3,PIK3C2B,PIK3CD,PIK3R1,PIK3R3 |
| Renin-angiotensin signaling | 1,98E00 | 1,49E-01 | -0,243 | ATF2,ATM,FGFR3,FRS2,ITPR1,MAPK10,MAPK12,MAPK8,PAK6,PIK3C2B,PIK3CD,PIK3R1,PIK3R3,PRKAR1B,PRKCH,PTK2,RELA,STAT1 |
| Il-7 signaling pathway | 1,98E00 | 1,63E-01 | -0,277 | ATM,CDC25A,CDK2,FGFR3,FRS2,MAPK12,MET,PAX5,PIK3C2B,PIK3CD,PIK3R1,PIK3R3,PTK2,STAT1 |
| Prostate cancer signaling | 1,96E00 | 1,58E-01 | 0,000 | AR,ATF2,ATM,CCNE1,CDK2,FGFR3,FRS2,HSP90B1,IKBKG,NFKBID,PIK3C2B,PIK3CD,PIK3R1,PIK3R3,RELA |
| Cd40 signaling | 1,95E00 | 1,67E-01 | 0,277 | ATM,FGFR3,FRS2,IKBKG,MAPK10,MAPK12,MAPK8,NFKBID,PIK3C2B,PIK3CD,PIK3R1,PIK3R3,RELA |
| Il-17a signaling in airway cells | 1,95E00 | 1,67E-01 | 0,277 | ATM,FGFR3,FRS2,IKBKG,MAPK10,MAPK12,MAPK8,NFKBID,PIK3C2B,PIK3CD,PIK3R1,PIK3R3,RELA |
| Antioxidant action of vitamin c | 1,95E00 | 1,54E-01 | 3,000 | CSF2,CSF2RA,IKBKG,MAPK10,MAPK12,MAPK8,NFKBID,PAFAH1B2,PLA2G1B,PLCB3,PLCB4,PLCE1,PLCH1,PLCL1,PLD1,RELA |
| Nnos signaling in neurons | 1,93E00 | 1,96E-01 | -2,000 | CAMK2A,CAPN3,CAPN8,DLG2,DLG4,GRIN2A,PFKM,PPP3CA,PRKCH |
| Germ cell-sertoli cell junction signaling | 1,92E00 | 1,36E-01 | 0,000 | ACTC1,AFDN,ATM,FGFR3,FRS2,MAP3K6,MAP3K8,MAPK10,MAPK12,MAPK8,NECTIN3,PAK6,PIK3C2B,PIK3CD,PIK3R1,PIK3R3,PLS1,PTK2,PXN,RHOT1,SORBS1,SRC,TUBA8 |
| Erbb signaling | 1,88E00 | 1,55E-01 | -0,258 | ATM,EGFR,FGFR3,FRS2,MAPK10,MAPK12,MAPK8,PAK6,PIK3C2B,PIK3CD,PIK3R1,PIK3R3,PRKCH,RPS6KB1,TGFA |
| Mitotic roles of polo-like kinase | 1,88E00 | 1,75E-01 | 0,447 | CCNB3,CDC16,CDC25A,CHEK2,FZR1,HSP90B1,KIF23,PPP2R2B,PPP2R3A,PPP2R5D,PTPA |
| Guanosine nucleotides degradation iii | 1,87E00 | 3,33E-01 | 0,000 | ACPP,NT5C2,NT5C3A,NT5M |
| Regulation of cellular mechanics by calpain protease | 1,87E00 | 1,82E-01 | 0,000 | CAPN3,CAPN8,CAST,CCNE1,CDK2,EGFR,ITGA4,PTK2,PXN,SRC |
| 4-1bb signaling in t lymphocytes | 1,86E00 | 2,19E-01 | -0,816 | ATF2,IKBKG,MAPK10,MAPK12,MAPK8,NFKBID,RELA |
| Il-12 signaling and production in macrophages | 1,83E00 | 1,39E-01 | 0,000 | APOC1,ATM,FGFR3,FRS2,IKBKG,IL18,MAP3K8,MAPK10,MAPK12,MAPK8,NOS2,PIK3C2B,PIK3CD,PIK3R1,PIK3R3,PPARG,PRKCH,RELA,STAT1,STAT6 |
| Erythropoietin signaling | 1,82E00 | 1,6E-01 | 0,000 | ATM,FGFR3,FRS2,IKBKG,NFKBID,PIK3C2B,PIK3CD,PIK3R1,PIK3R3,PRKCH,RELA,RPS6KB1,SRC |
| Glutamate receptor signaling | 1,82E00 | 1,79E-01 | -1,414 | DLG4,GRIA1,GRIA3,GRIA4,GRID2,GRIK1,GRIN2A,GRM8,HOMER1,SLC1A7 |
| Ccr3 signaling in eosinophils | 1,81E00 | 1,43E-01 | -0,832 | ATM,FGFR3,FRS2,GNB4,GNB5,ITPR1,MAPK12,PAK6,PIK3C2B,PIK3CD,PIK3R1,PIK3R3,PLA2G1B,PLCB3,PLCB4,PPP1R12A,PPP1R12B,PRKCH |
| Antiproliferative role of somatostatin receptor 2 | 1,8E00 | 1,64E-01 | -0,707 | ATM,FGFR3,FRS2,GNB4,GNB5,GUCY1A2,GUCY1B1,PIK3C2B,PIK3CD,PIK3R1,PIK3R3,SRC |
| Cxcr4 signaling | 1,79E00 | 1,34E-01 | -1,147 | ATM,FGFR3,FRS2,GNAO1,GNB4,GNB5,ITPR1,MAPK10,MAPK12,MAPK8,PAK6,PIK3C2B,PIK3CD,PIK3R1,PIK3R3,PLCB3,PLCB4,PRKCH,PTK2,PXN,RHOT1,SRC |
| Aryl hydrocarbon receptor signaling | 1,79E00 | 1,4E-01 | -0,535 | ALDH3A2,ALDH4A1,ATM,CCNE1,CDK2,CHEK2,FAS,GSTM1,HSP90B1,HSPB7,MAPK8,NCOR2,NFIB,NRIP1,POLA1,RELA,RXRB,SRC,TP73 |
| Fgf signaling | 1,77E00 | 1,54E-01 | -1,069 | ATF2,ATM,FGF1,FGF17,FGFR3,FRS2,ITPR1,MAPK12,MAPK8,MET,PIK3C2B,PIK3CD,PIK3R1,PIK3R3 |
| Pak signaling | 1,77E00 | 1,5E-01 | -1,291 | ATM,EPHA3,FGFR3,FRS2,ITGA4,MAPK10,MAPK12,MAPK8,PAK6,PIK3C2B,PIK3CD,PIK3R1,PIK3R3,PTK2,PXN |
| Role of pkr in interferon induction and antiviral response | 1,76E00 | 1,95E-01 | 0,000 | ATF2,BID,CASP8,EIF2S1,IKBKG,NFKBID,RELA,STAT1 |
| Stat3 pathway | 1,75E00 | 1,62E-01 | -1,155 | CDC25A,EGFR,FGFR3,MAP3K21,MAPK10,MAPK12,MAPK8,NTRK2,SOCS4,SOCS7,SRC,TNFRSF11A |
| Urate biosynthesis/inosine 5'-phosphate degradation | 1,74E00 | 3,08E-01 | 0,000 | ACPP,NT5C2,NT5C3A,NT5M |
| Ampk signaling | 1,72E00 | 1,26E-01 | -0,626 | AK5,ATF2,ATM,CHRNA7,DPF1,FGFR3,FRS2,GYS1,MAPK12,PFKFB3,PFKFB4,PFKM,PIK3C2B,PIK3CD,PIK3R1,PIK3R3,PPARGC1A,PPM1B,PPP2R2B,PPP2R3A,PPP2R5D,PRKAR1B,PTPA,RAB27A,RPS6KB1,SIRT1,SRC |
| 4-hydroxyproline degradation i | 1,71E00 | 6,67E-01 | 0,000 | ALDH4A1,HOGA1 |
| Glycerol-3-phosphate shuttle | 1,71E00 | 6,67E-01 | 0,000 | GPD1,GPD2 |
| Il-8 signaling | 1,71E00 | 1,28E-01 | -1,043 | ATM,EGFR,FGFR3,FRS2,GNB4,GNB5,IKBKG,IRAK1,LASP1,MAPK10,MAPK12,MAPK8,NOX4,PIK3C2B,PIK3CD,PIK3R1,PIK3R3,PLD1,PRKCH,PTK2,RELA,RHOT1,RPS6KB1,SRC,VEGFA |
| Lymphotoxin β receptor signaling | 1,69E00 | 1,64E-01 | -0,707 | ATM,DIABLO,FGFR3,FRS2,IKBKG,NFKBID,PIK3C2B,PIK3CD,PIK3R1,PIK3R3,RELA |
| Il-15 signaling | 1,67E00 | 1,58E-01 | 0,000 | ATM,CSF2,FGFR3,FRS2,MAPK12,PIK3C2B,PIK3CD,PIK3R1,PIK3R3,PTK2,RELA,STAT6 |
| Gdnf family ligand-receptor interactions | 1,67E00 | 1,58E-01 | -0,632 | ARTN,ATM,FGFR3,FRS2,ITPR1,MAPK10,MAPK12,MAPK8,PIK3C2B,PIK3CD,PIK3R1,PIK3R3 |
| Il-17 signaling | 1,66E00 | 1,53E-01 | 0,000 | ATF2,ATM,FGFR3,FRS2,MAPK10,MAPK12,MAPK8,NOS2,PIK3C2B,PIK3CD,PIK3R1,PIK3R3,RELA |
| Clathrin-mediated endocytosis signaling | 1,66E00 | 1,26E-01 | 0,000 | ACTC1,ACTR2,AP1B1,AP1G2,AP2B1,APOC1,ATM,CSNK2B,CTTN,FGF1,FGF17,FGFR3,FRS2,HSPA8,MET,PIK3C2B,PIK3CD,PIK3R1,PIK3R3,PIP5K1C,PPP3CA,SH3GLB2,SRC,SYNJ1,VEGFA |
| Acute phase response signaling | 1,66E00 | 1,3E-01 | -0,229 | C4BPB,FN1,HNRNPK,IKBKG,IL18,IL1R1,IL1RAP,IL37,IRAK1,ITIH4,MAPK12,MAPK8,NFKBID,NR3C1,PIK3CD,PIK3R1,PIK3R3,RELA,SOCS4,SOCS7,TCF3,TNFRSF1B |
| Wnt/β-catenin signaling | 1,66E00 | 1,3E-01 | -0,447 | ACVR1,APC2,CSNK1E,CSNK1G1,CSNK2B,GNAO1,LRP1,MARK2,NR5A2,POU5F1,PPP2R2B,PPP2R3A,PPP2R5D,PTPA,SOX13,SOX5,SRC,TCF3,TCF7L2,WNT3,WNT5B,WNT8B |
| Mif regulation of innate immunity | 1,64E00 | 1,86E-01 | -0,707 | IKBKG,MAPK10,MAPK12,MAPK8,NFKBID,NOS2,PLA2G1B,RELA |
| Chemokine signaling | 1,64E00 | 1,62E-01 | -2,111 | CAMK2A,CAMK2B,CAMK2D,MAPK12,MAPK8,PLCB3,PLCB4,PPP1R12A,PPP1R12B,PTK2,SRC |
| Glioma signaling | 1,63E00 | 1,42E-01 | -0,277 | ATM,CAMK2A,CAMK2B,CAMK2D,E2F8,EGFR,FGFR3,FRS2,IDH3B,IDH3G,PIK3C2B,PIK3CD,PIK3R1,PIK3R3,PRKCH,TGFA |
| Neuregulin signaling | 1,62E00 | 1,51E-01 | -1,000 | ADAM17,DLG4,EGFR,ERBIN,GRB7,HSP90B1,ITGA4,PIK3R1,PIK3R3,PRKCH,RPS6KB1,SRC,TGFA |
| Docosahexaenoic acid (dha) signaling | 1,6E00 | 1,73E-01 | 0,000 | APP,ATM,BID,FGFR3,FRS2,PIK3C2B,PIK3CD,PIK3R1,PIK3R3 |
| Nad salvage pathway ii | 1,6E00 | 2,38E-01 | 0,000 | ACPP,NT5C2,NT5C3A,NT5M,PXYLP1 |
| P2y purigenic receptor signaling pathway | 1,59E00 | 1,35E-01 | -1,000 | ATF2,ATM,FGFR3,FRS2,GNB4,GNB5,PIK3C2B,PIK3CD,PIK3R1,PIK3R3,PLCB3,PLCB4,PLCE1,PLCH1,PLCL1,PRKAR1B,PRKCH,RELA |
| Nf-κb activation by viruses | 1,59E00 | 1,49E-01 | -0,277 | ATM,FGFR3,FRS2,IKBKG,ITGA4,ITGAL,NFKBID,PIK3C2B,PIK3CD,PIK3R1,PIK3R3,PRKCH,RELA |
| Sphingosine-1-phosphate signaling | 1,58E00 | 1,37E-01 | -1,213 | ATM,CASP8,FGFR3,FRS2,PIK3C2B,PIK3CD,PIK3R1,PIK3R3,PLCB3,PLCB4,PLCE1,PLCH1,PLCL1,PTK2,RHOT1,SMPD3,SPHK1 |
| Melatonin signaling | 1,56E00 | 1,57E-01 | -0,905 | CAMK2A,CAMK2B,CAMK2D,GNAO1,PLCB3,PLCB4,PLCE1,PLCH1,PLCL1,PRKAR1B,PRKCH |
| Erbb4 signaling | 1,56E00 | 1,57E-01 | 0,333 | ADAM17,APH1B,ATM,FGFR3,FRS2,NCSTN,PIK3C2B,PIK3CD,PIK3R1,PIK3R3,PRKCH |
| Regulation of il-2 expression in activated and anergic t lymphocytes | 1,55E00 | 1,52E-01 | 0,000 | IKBKG,LAT,MAPK10,MAPK12,MAPK8,NFATC2,NFATC3,NFKBID,PPP3CA,RELA,VAV2,ZAP70 |
| Tnfr2 signaling | 1,54E00 | 2,07E-01 | -1,633 | IKBKG,MAPK8,NAIP,NFKBID,RELA,TNFRSF1B |
| Cell cycle regulation by btg family proteins | 1,53E00 | 1,89E-01 | 0,000 | CCNE1,CDK2,E2F8,PPP2R2B,PPP2R3A,PPP2R5D,PTPA |
| Cdk5 signaling | 1,52E00 | 1,43E-01 | 0,000 | FOSB,MAPK10,MAPK12,MAPK7,MAPK8,MAPT,NTRK2,PPP1R12A,PPP1R7,PPP2R2B,PPP2R3A,PPP2R5D,PRKAR1B,PTPA |
| Adenosine nucleotides degradation ii | 1,52E00 | 2,67E-01 | 0,000 | ACPP,NT5C2,NT5C3A,NT5M |
| Factors promoting cardiogenesis in vertebrates | 1,51E00 | 1,46E-01 | 0,000 | ACVR1,ATF2,CCNE1,CDK2,LRP1,MEF2C,NODAL,NOX4,PRKCH,SMAD9,TCF3,TCF7L2,WNT3 |
| Crosstalk between dendritic cells and natural killer cells | 1,51E00 | 1,46E-01 | 0,000 | ACTC1,CAMK2A,CAMK2B,CAMK2D,CSF2,FAS,ICAM3,IL18,ITGAL,MICB,RELA,TNFRSF1B,TNFSF10 |
| Apoptosis signaling | 1,51E00 | 1,46E-01 | -1,387 | BID,CAPN3,CAPN8,CASP8,DIABLO,FAS,GAS2,IKBKG,MAPK8,NAIP,NFKBID,RELA,TNFRSF1B |
| Tight junction signaling | 1,48E00 | 1,27E-01 | 0,000 | ACTC1,AFDN,CASK,CLDN15,CLDN2,CLDN7,CPSF3,GPAA1,MAGI2,MARK2,MYH7B,NECTIN3,NSF,PPP2R2B,PPP2R3A,PPP2R5D,PRKAR1B,PTPA,RELA,TJP2,TNFRSF1B |
| Insulin receptor signaling | 1,48E00 | 1,31E-01 | 0,000 | ATM,FGFR3,FRS2,GRB10,GYS1,INPP5J,MAPK8,PIK3C2B,PIK3CD,PIK3R1,PIK3R3,PPP1R12A,PPP1R7,PRKAR1B,RPS6KB1,SGK1,SYNJ1,SYNJ2 |
| Renal cell carcinoma signaling | 1,48E00 | 1,48E-01 | -0,333 | ATM,EGLN3,FGFR3,FRS2,MET,PAK6,PIK3C2B,PIK3CD,PIK3R1,PIK3R3,TGFA,VEGFA |
| Macropinocytosis signaling | 1,48E00 | 1,48E-01 | -0,577 | ABI1,ATM,CSF1R,FGFR3,FRS2,MET,PIK3C2B,PIK3CD,PIK3R1,PIK3R3,PRKCH,SRC |
| Opioid signaling pathway | 1,46E00 | 1,18E-01 | -0,577 | AP1B1,AP1G2,AP2B1,ATF2,CACNA1C,CACNA2D1,CACNA2D3,CACNB2,CAMK2A,CAMK2B,CAMK2D,FOSB,GNAO1,GRIN2A,ITPR1,MAPK12,MAPK7,OPRK1,PPP3CA,PRKAR1B,PRKCH,RGS11,RGS20,RGS3,RGS7,RPS6KB1,SRC,TH |
| Hepatic cholestasis | 1,44E00 | 1,27E-01 | 0,000 | CSF2,FABP6,IKBKG,IL17B,IL18,IL1R1,IL1RAP,IL37,IRAK1,MAPK10,MAPK12,MAPK8,NFKBID,NR5A2,PRKAR1B,PRKCH,RELA,TIRAP,TJP2,TNFRSF1B |
| 2-ketoglutarate dehydrogenase complex | 1,44E00 | 0,5 | 0,000 | DLST,OGDH |
| Cardiac β-adrenergic signaling | 1,43E00 | 1,29E-01 | -0,577 | AKAP12,AKAP14,AKAP6,AKAP7,ATP2A1,ATP2A3,CACNA1C,GNB4,GNB5,PDE4A,PKIB,PPP1R12A,PPP1R7,PPP2R2B,PPP2R3A,PPP2R5D,PRKAR1B,PTPA |
| Natural killer cell signaling | 1,42E00 | 1,33E-01 | 0,000 | ATM,FGFR3,FRS2,INPP5J,LAT,PAK6,PIK3C2B,PIK3CD,PIK3R1,PIK3R3,PRKCH,SH3BP2,SYNJ1,SYNJ2,VAV2,ZAP70 |
| Sperm motility | 1,42E00 | 1,33E-01 | -1,500 | GUCY1A2,GUCY1B1,ITPR1,PAFAH1B2,PDE4A,PLA2G1B,PLCB3,PLCB4,PLCE1,PLCH1,PLCL1,PRKAR1B,PRKCH,PRKG1,PTK2,TWF1 |
| p38 mapk signaling | 1,42E00 | 1,33E-01 | 0,000 | ATF2,DAXX,FAS,HSPB7,IL18,IL1R1,IL1RAP,IL37,IRAK1,MAPK12,MAPT,MEF2C,PLA2G1B,RPS6KB1,STAT1,TNFRSF1B |
| Il-1 signaling | 1,41E00 | 1,41E-01 | -0,302 | GNAO1,GNB4,GNB5,IKBKG,IL1R1,IL1RAP,IRAK1,MAPK10,MAPK12,MAPK8,NFKBID,PRKAR1B,RELA |
| Ppar signaling | 1,41E00 | 1,41E-01 | 1,387 | HSP90B1,IKBKG,IL18,IL1R1,IL1RAP,IL37,NCOR2,NFKBID,NRIP1,PPARG,PPARGC1A,RELA,TNFRSF1B |
| Cell cycle control of chromosomal replication | 1,41E00 | 1,61E-01 | 0,000 | CDK15,CDK2,CDK20,CHEK2,DNA2,LIG1,MCM8,ORC1,POLA1 |
| Melatonin degradation i | 1,41E00 | 1,61E-01 | 0,000 | CSGALNACT1,CYP2E1,CYP2U1,CYP4X1,EXT2,SULT1A1,SULT1C2,SULT4A1,UGT1A7 (includes others) |
| Erk/mapk signaling | 1,41E00 | 1,21E-01 | -1,225 | ATF2,ATM,FGFR3,FRS2,HSPB7,ITGA4,PAK6,PIK3C2B,PIK3CD,PIK3R1,PIK3R3,PLA2G1B,PPARG,PPP1R12A,PPP1R7,PPP2R2B,PPP2R3A,PPP2R5D,PRKAR1B,PTK2,PTPA,PXN,SRC,STAT1 |
| Flt3 signaling in hematopoietic progenitor cells | 1,4E00 | 1,45E-01 | -0,577 | ATF2,ATM,FGFR3,FRS2,MAPK12,PIK3C2B,PIK3CD,PIK3R1,PIK3R3,RPS6KB1,STAT1,STAT6 |
| Prolactin signaling | 1,4E00 | 1,45E-01 | 0,302 | ATM,FGFR3,FRS2,NR3C1,PIK3C2B,PIK3CD,PIK3R1,PIK3R3,PRKCH,SOCS4,SOCS7,STAT1 |
| Jak/stat signaling | 1,4E00 | 1,45E-01 | 0,577 | ATM,FGFR3,FRS2,PIK3C2B,PIK3CD,PIK3R1,PIK3R3,RELA,SOCS4,SOCS7,STAT1,STAT6 |
| Thrombopoietin signaling | 1,4E00 | 1,54E-01 | 0,632 | ATM,FGFR3,FRS2,MPL,PIK3C2B,PIK3CD,PIK3R1,PIK3R3,PRKCH,STAT1 |
| Tnfr1 signaling | 1,38E00 | 1,67E-01 | -0,707 | BID,CASP8,IKBKG,MAPK8,NAIP,NFKBID,PAK6,RELA |
| Role of pattern recognition receptors in recognition of bacteria and viruses | 1,38E00 | 1,3E-01 | -0,277 | ATM,CSF2,EIF2S1,FGFR3,FRS2,IL17B,IL18,MAPK10,MAPK12,MAPK8,OAS1,PIK3C2B,PIK3CD,PIK3R1,PIK3R3,PRKCH,RELA |
| Hmgb1 signaling | 1,38E00 | 1,3E-01 | -0,728 | ATM,CSF2,FGFR3,FRS2,IL17B,IL18,IL1R1,MAPK10,MAPK12,MAPK8,PIK3C2B,PIK3CD,PIK3R1,PIK3R3,RELA,RHOT1,TNFRSF1B |
| Tca cycle ii (eukaryotic) | 1,36E00 | 2,08E-01 | 0,000 | DLST,IDH3B,IDH3G,LIPF,OGDH |
| Inhibition of angiogenesis by tsp1 | 1,35E00 | 1,88E-01 | -1,633 | GUCY1A2,GUCY1B1,MAPK10,MAPK12,MAPK8,VEGFA |
| Integrin signaling | 1,35E00 | 1,18E-01 | -1,043 | ACTC1,ACTR2,ATM,CAPN3,CAPN8,CTTN,FGFR3,FRS2,GRB7,ITGA10,ITGA4,ITGAL,LIMS1,MAPK8,PAK6,PIK3C2B,PIK3CD,PIK3R1,PIK3R3,PPP1R12A,PPP1R12B,PTK2,PXN,RHOT1,SRC |
| Salvage pathways of pyrimidine ribonucleotides | 1,35E00 | 1,38E-01 | 0,000 | AK5,CDK2,DAPK1,DMPK,IRAK1,MAK,MAP3K6,MAP3K8,MAPK7,MAPK8,NEK2,PRKCH,SGK1 |
| Cardiac hypertrophy signaling | 1,34E00 | 1,16E-01 | -0,816 | ATF2,ATM,CACNA1C,FGFR3,FRS2,GNAO1,GNB4,GNB5,MAP3K6,MAP3K8,MAPK10,MAPK12,MAPK8,MEF2C,PIK3C2B,PIK3CD,PIK3R1,PIK3R3,PLCB3,PLCB4,PLCE1,PLCH1,PLCL1,PPP3CA,PRKAR1B,RHOT1,RPS6KB1 |
| Growth hormone signaling | 1,34E00 | 1,41E-01 | 0,000 | ATM,FGFR3,FRS2,PIK3C2B,PIK3CD,PIK3R1,PIK3R3,PRKCH,RPS6KB1,SOCS4,SOCS7,STAT1 |
| Phagosome formation | 1,34E00 | 1,3E-01 | 0,000 | ATM,FGFR3,FN1,FRS2,ITGA4,PIK3C2B,PIK3CD,PIK3R1,PIK3R3,PLCB3,PLCB4,PLCE1,PLCH1,PLCL1,PRKCH,RHOT1 |
| Relaxin signaling | 1,3E00 | 1,23E-01 | 1,291 | ATM,FGFR3,FRS2,GNAO1,GNB4,GNB5,GUCY1A2,GUCY1B1,IKBKG,NFKBID,NOS2,PDE4A,PIK3C2B,PIK3CD,PIK3R1,PIK3R3,PRKAR1B,RELA,VEGFA |
| Il-17a signaling in gastric cells | 1,3E00 | 0,2 | 0,447 | EGFR,MAPK10,MAPK12,MAPK8,RELA |

**Supplemental Table S8. Complete list of significant (p-value ≤ 0,05) enriched canonical pathways of the differentially expressed genes using Ingenuity Pathway Analysis (IPA) software for high fat (HF) treatment.**

| HIGH FAT Treatment |  |  |  |  |
| --- | --- | --- | --- | --- |
| Ingenuity canonical pathways | -log(p-value) | Ratio | Z-score | Molecules |
| Calcium signaling | 9,77e00 | 2,37e-01 | 0,557 | ACTC1,ASPH,ATF2,ATP2A1,ATP2A3,ATP2B1,ATP2C1,CACNA1C,CACNA2D1,CACNA2D2,CACNA2D3,CACNB1,CACNB2,CACNG7,CAMK2A,CAMK2B,CAMK2D,CAMK2G,CAMKK1,CHRNB4,CREB3L4,GRIA2,GRIA3,GRIA4,HDAC10,HDAC11,HDAC6,ITPR1,MAPK1,MYH9,NFATC1,NFATC2,PPP3CA,PRKAR2A,RAP1B,RCAN1,RCAN2,RCAN3,RYR1,RYR2,SLC8A1,TNNC2,TNNI1,TPM1,TRPC1,TRPC3,TRPM8 |
| Amyloid processing | 5,52e00 | 3,2e-01 | 0,000 | AKT1,AKT3,APH1B,APP,BACE1,CAPN1,CAPN3,CAPN8,CSNK2B,MAPK1,MAPK12,MAPK14,MAPT,MARK1,NCSTN,PRKAR2A |
| B cell receptor signaling | 5,25e00 | 1,91e-01 | -0,686 | AKT1,AKT3,ATF2,BLNK,CAMK2A,CAMK2B,CAMK2D,CAMK2G,CARD10,CREB3L4,EBF1,FRS2,GAB2,INPP5J,MAP3K13,MAP3K14,MAP3K4,MAP3K6,MAP3K7,MAP3K8,MAPK1,MAPK12,MAPK14,MAPK8,NFATC1,NFATC2,NFKBID,PIK3R3,PPP3CA,PTK2,PTPN6,PTPRC,RAP1B,RASSF5,SYNJ2,VAV3 |
| Sertoli cell-sertoli cell junction signaling | 4,83e00 | 1,91e-01 | 0,000 | ACTC1,ACTN4,AFDN,AKT1,AKT3,ATF2,CLDN14,CLDN2,CLDN6,CTNNA1,CTNNA2,ITGA4,MAGI2,MAP3K13,MAP3K14,MAP3K4,MAP3K6,MAP3K7,MAP3K8,MAPK1,MAPK12,MAPK14,MAPK8,NECTIN3,OCLN,PLS1,PRKAR2A,PRKG1,SORBS1,SPTB,TGFB3,TGFBR3,TJP2 |
| Role of nfat in cardiac hypertrophy | 4,62e00 | 1,76e-01 | -0,822 | AKT1,AKT3,CACNA1C,CACNA2D1,CACNA2D2,CACNA2D3,CACNB1,CACNB2,CACNG7,CAMK2A,CAMK2B,CAMK2D,CAMK2G,FRS2,GNB4,HDAC10,HDAC11,HDAC6,ITPR1,MAP3K7,MAPK1,MAPK12,MAPK14,MAPK8,NOTUM,PIK3R3,PLCB4,PLCD4,PLCH1,PLCL2,PPP3CA,PRKAR2A,PRKCH,RCAN1,RCAN2,RCAN3,SLC8A1,TGFB3 |
| Gnrh signaling | 4,6e00 | 1,91e-01 | -1,633 | ATF2,CACNA1C,CACNA2D1,CACNA2D2,CACNA2D3,CACNB1,CACNB2,CACNG7,CAMK2A,CAMK2B,CAMK2D,CAMK2G,CREB3L4,ITPR1,MAP3K13,MAP3K14,MAP3K4,MAP3K6,MAP3K7,MAP3K8,MAPK1,MAPK12,MAPK14,MAPK7,MAPK8,MMP2,PLCB4,PRKAR2A,PRKCH,PTK2,PXN |
| Netrin signaling | 4,52e00 | 2,62e-01 | 0,000 | ABLIM2,ABLIM3,CACNA1C,CACNA2D1,CACNA2D2,CACNA2D3,CACNB1,CACNB2,CACNG7,NFATC1,NFATC2,PPP3CA,PRKAR2A,PRKG1,RYR1,RYR2,UNC5C |
| Protein kinase a signaling | 4,28e00 | 1,48e-01 | -1,342 | AKAP12,AKAP2,AKAP6,AKAP7,AKAP9,ATF2,CAMK2A,CAMK2B,CAMK2D,CAMK2G,CDC25A,CDKN3,CREB3L4,CREM,GNB4,GYS2,ITPR1,LEF1,MAPK1,MTMR3,NFATC1,NFATC2,NFKBID,NGFR,NOTUM,PDE1A,PDE4A,PDE4C,PDE8A,PLCB4,PLCD4,PLCH1,PLCL2,PPP1R11,PPP1R7,PPP3CA,PRKAR2A,PRKCH,PTK2,PTPDC1,PTPN13,PTPN2,PTPN22,PTPN6,PTPRB,PTPRC,PTPRK,PXN,PYGL,RAP1B,RYR1,RYR2,TCF4,TCF7L2,TDP2,TGFB3,TNNI1 |
| Pi3k signaling in b lymphocytes | 4,1e00 | 1,98e-01 | -1,400 | AKT1,AKT3,ATF2,ATF6B,ATF7,BLNK,CAMK2A,CAMK2B,CAMK2D,CAMK2G,CARD10,FYN,ITPR1,MAPK1,NFATC1,NFATC2,NFKBID,NOTUM,PLCB4,PLCD4,PLCH1,PLCL2,PPP3CA,PTPRC,VAV3 |
| Synaptic long term potentiation | 4,08e00 | 2,02e-01 | -1,460 | ATF2,CACNA1C,CAMK2A,CAMK2B,CAMK2D,CAMK2G,CREB3L4,GRIA2,GRIA3,GRIA4,GRM8,ITPR1,MAPK1,NOTUM,PLCB4,PLCD4,PLCH1,PLCL2,PPP1R11,PPP1R7,PPP3CA,PRKAR2A,PRKCH,RAP1B |
| Hgf signaling | 3,5e00 | 1,93e-01 | 0,000 | AKT1,AKT3,ATF2,CDK2,FRS2,ITGA4,MAP3K13,MAP3K14,MAP3K4,MAP3K6,MAP3K7,MAP3K8,MAPK1,MAPK12,MAPK8,MET,PIK3R3,PRKCH,PTK2,PXN,RAP1B,RAPGEF1 |
| Pkcθ signaling in t lymphocytes | 3,44e00 | 1,75e-01 | 0,000 | CACNA1C,CACNA2D1,CACNA2D2,CACNA2D3,CACNB1,CACNB2,CACNG7,CAMK2A,CAMK2B,CAMK2D,CAMK2G,FRS2,FYN,MAP3K13,MAP3K14,MAP3K4,MAP3K6,MAP3K7,MAP3K8,MAPK1,MAPK8,NFATC1,NFATC2,NFKBID,PIK3R3,PPP3CA,VAV3 |
| Molecular mechanisms of cancer | 3,37e00 | 1,39e-01 | 0,000 | AKT1,AKT3,APAF1,APH1B,ARHGEF1,ARHGEF4,ARHGEF7,AURKA,BAX,BID,CAMK2A,CAMK2B,CAMK2D,CAMK2G,CASP8,CDC25A,CDK15,CDK19,CDK2,CDK7,CTNNA1,CTNNA2,E2F5,FAS,FRS2,FYN,GAB2,GLI1,GNAO1,ITGA4,LEF1,LRP6,MAP3K7,MAPK1,MAPK12,MAPK14,MAPK8,MAX,NCSTN,NF1,NFKBID,PIK3R3,PLCB4,PRKAR2A,PRKCH,PTK2,RAP1B,RAPGEF1,RASGRP1,RBPJ,SMAD9,TCF4,TGFB3,WNT5B |
| Tight junction signaling | 3,26e00 | 1,69e-01 | 0,000 | ACTC1,AFDN,AKT1,AKT3,CASK,CLDN14,CLDN2,CLDN6,CPSF3,CTNNA1,GOSR2,GPAA1,MAGI2,MARK2,MYH9,NAPB,NECTIN3,NGFR,NSF,OCLN,PATJ,PPP2R3B,PRKAR2A,PTPA,STX16,TGFB3,TJP2,TNFRSF1B |
| Synaptic long term depression | 3,18e00 | 1,67e-01 | 0,000 | CACNA1C,CACNA2D1,CACNA2D2,CACNA2D3,CACNB1,CACNB2,CACNG7,GNAO1,GRIA2,GRIA3,GRIA4,GRM8,ITPR1,MAPK1,NOTUM,NPR3,PAFAH1B2,PLCB4,PLCD4,PLCH1,PLCL2,PNPLA8,PPP2R3B,PRKCH,PRKG1,PTPA,RYR1,RYR2 |
| Type ii diabetes mellitus signaling | 3,17e00 | 1,71e-01 | 0,000 | ACSL6,AKT1,AKT3,CACNA1C,CACNA2D1,CACNA2D2,CACNA2D3,CACNB1,CACNB2,CACNG7,CD36,FRS2,MAP3K14,MAP3K7,MAPK1,MAPK12,MAPK8,NFKBID,NGFR,PIK3R3,PPARG,PRKCH,SLC2A4,SOCS4,SOCS7,TNFRSF1B |
| Creb signaling in neurons | 3,17e00 | 1,57e-01 | -1,043 | AKT1,AKT3,ATF2,CACNA1C,CACNA2D1,CACNA2D2,CACNA2D3,CACNB1,CACNB2,CACNG7,CAMK2A,CAMK2B,CAMK2D,CAMK2G,CREB3L4,FRS2,GNAO1,GNB4,GRIA2,GRIA3,GRIA4,GRM8,ITPR1,MAPK1,NOTUM,PIK3R3,PLCB4,PLCD4,PLCH1,PLCL2,POLR2A,PRKAR2A,PRKCH |
| Calcium transport i | 3,06e00 | 0,5 | 0,000 | ATP2A1,ATP2A3,ATP2B1,ATP2C1,ATP2C2 |
| Death receptor signaling | 3,04e00 | 1,96e-01 | 0,000 | ACIN1,ACTC1,APAF1,BID,CASP8,FAS,GAS2,HSPB7,MAP3K14,MAPK8,NFKBID,PARP8,TIPARP,TNFRSF1B,TNFRSF25,TNFSF10,TNFSF15,ZC3HAV1 |
| Cd27 signaling in lymphocytes | 2,84e00 | 2,31e-01 | 0,302 | APAF1,BID,CASP8,MAP3K13,MAP3K14,MAP3K4,MAP3K6,MAP3K7,MAP3K8,MAPK12,MAPK8,NFKBID |
| Superpathway of inositol phosphate compounds | 2,84e00 | 1,49e-01 | 0,000 | ACP6,CDC25A,DUSP14,ERBB3,ERBB4,EYA4,FRS2,FYN,INPP5A,INPP5J,ITPKB,MET,MINPP1,NUDT9,PIK3R3,PIP5K1A,PLCB4,PLCD4,PLCH1,PPFIA4,PPIP5K1,PPP1R7,PPP3CA,PRUNE1,PTPA,PTPN13,PTPN2,PTPN22,PTPN6,PTPRC,PXYLP1,SEC16A,STYXL1,SYNJ2 |
| Acute phase response signaling | 2,8e00 | 1,6e-01 | -0,209 | AKT1,AKT3,APOH,C4BPB,CRABP1,HNRNPK,IL1RAP,IL1RN,IL6R,ITIH2,ITIH4,MAP3K14,MAP3K7,MAPK1,MAPK12,MAPK14,MAPK8,MYD88,NFKBID,NGFR,PIK3R3,SERPINA1,SERPINA3,SOCS4,SOCS7,TCF4,TNFRSF1B |
| Nnos signaling in skeletal muscle cells | 2,73e00 | 2,5e-01 | 0,000 | CACNA1C,CACNA2D1,CACNA2D2,CACNA2D3,CACNB1,CACNB2,CACNG7,CAPN3,RYR1,RYR2 |
| Neuropathic pain signaling in dorsal horn neurons | 2,71e00 | 1,75e-01 | 0,000 | BDNF,CAMK2A,CAMK2B,CAMK2D,CAMK2G,FRS2,GRIA2,GRIA3,GRIA4,GRM8,ITPR1,MAPK1,NOTUM,PIK3R3,PLCB4,PLCD4,PLCH1,PLCL2,PRKAR2A,PRKCH |
| Rank signaling in osteoclasts | 2,62e00 | 1,8e-01 | 0,000 | AKT1,AKT3,FRS2,MAP3K13,MAP3K14,MAP3K4,MAP3K6,MAP3K7,MAP3K8,MAPK1,MAPK12,MAPK14,MAPK8,NFATC1,NFATC2,NFKBID,PIK3R3,PPP3CA |
| Melatonin signaling | 2,59e00 | 0,2 | 0,277 | CAMK2A,CAMK2B,CAMK2D,CAMK2G,GNAO1,MAPK1,NOTUM,PLCB4,PLCD4,PLCH1,PLCL2,PRKAR2A,PRKCH,SLC2A4 |
| Opioid signaling pathway | 2,56e00 | 1,43e-01 | 0,174 | AKT1,AKT3,ATF2,CACNA1C,CACNA2D1,CACNA2D2,CACNA2D3,CACNB1,CACNB2,CACNG7,CAMK2A,CAMK2B,CAMK2D,CAMK2G,CREB3L4,FOSB,FYN,GNAO1,ITPR1,MAPK1,MAPK12,MAPK15,MAPK7,PDE1A,PPP3CA,PRKAR2A,PRKCH,RAP1B,RGS10,RGS20,RGS3,RPS6KA2,RYR1,RYR2 |
| Pyridoxal 5'-phosphate salvage pathway | 2,5e00 | 2,03e-01 | 0,000 | CDK2,CDK7,DAPK1,DMPK,HIPK1,MAK,MAP3K6,MAP3K8,MAPK1,MAPK7,MAPK8,NEK2,PRKCH |
| 3-phosphoinositide degradation | 2,49e00 | 1,58e-01 | 0,000 | ACP6,CDC25A,DUSP14,EYA4,INPP4B,INPP5J,MINPP1,MTMR1,MTMR3,NUDT9,PPFIA4,PPIP5K1,PPP1R7,PPP3CA,PRUNE1,PTPA,PTPN13,PTPN2,PTPN22,PTPN6,PTPRC,PXYLP1,STYXL1,SYNJ2 |
| Role of macrophages, fibroblasts and endothelial cells in rheumatoid arthritis | 2,48e00 | 1,35e-01 | 0,000 | AKT1,AKT3,ATF2,CAMK2A,CAMK2B,CAMK2D,CAMK2G,CREB3L4,DKK2,FRS2,GNAO1,IL17RC,IL1RAP,IL1RN,IL6R,LEF1,LRP6,MAP3K14,MAP3K7,MAPK1,MAPK14,MYD88,NFATC1,NFATC2,NFKBID,NGFR,NOTUM,PIK3R3,PLCB4,PLCD4,PLCH1,PLCL2,PPP3CA,PRKCH,PRSS2,PRSS3,TCF4,TCF7L2,TLR7,TNFRSF1B,WNT5B |
| Superpathway of d-myo-inositol (1,4,5)-trisphosphate metabolism | 2,45e00 | 2,92e-01 | 0,000 | IMPA1,INPP5A,INPP5J,ITPKB,MINPP1,SEC16A,SYNJ2 |
| Axonal guidance signaling | 2,45e00 | 1,26e-01 | 0,000 | ABLIM2,ABLIM3,ADAM15,ADAM17,ADAM22,ADAM28,ADAM8,AKT1,AKT3,ARHGEF7,BDNF,ECE2,EFNA1,ENPEP,EPHA3,EPHA4,FES,FRS2,FYN,GLI1,GNAO1,GNB4,ITGA4,LINGO1,LRRC4C,MAPK1,MET,MME,MMP2,NFATC1,NFATC2,NGEF,NGFR,NOTUM,PIK3R3,PLCB4,PLCD4,PLCH1,PLCL2,PLXNB1,PPP3CA,PRKAR2A,PRKCH,PTK2,PXN,RAP1B,RASSF5,RGS3,RTN4,SEMA3F,SEMA4A,SEMA6B,SLIT2,UNC5C,WIPF1,WNT5B |
| Ngf signaling | 2,44e00 | 1,67e-01 | 0,447 | AKT1,AKT3,ATF2,BAX,CREB3L4,FRS2,MAP3K13,MAP3K14,MAP3K4,MAP3K6,MAP3K7,MAP3K8,MAPK1,MAPK12,MAPK7,MAPK8,NGFR,PIK3R3,RAP1B,RPS6KA2 |
| Notch signaling | 2,42e00 | 2,43e-01 | 0,447 | ADAM17,APH1B,CNTN1,DTX2,FURIN,HEY1,HEY2,NCSTN,RBPJ |
| Leukocyte extravasation signaling | 2,42e00 | 1,46e-01 | -0,192 | ACTC1,ACTN4,AFDN,ARHGAP12,ARHGAP4,ARHGAP6,CLDN14,CLDN2,CLDN6,CTNNA1,CTNNA2,FRS2,ITGA4,ITGAL,MAP3K4,MAPK1,MAPK12,MAPK14,MAPK8,MMP2,MMP28,PIK3R3,PRKCH,PTK2,PXN,RAP1B,RASGRP1,RASSF5,VAV3,WIPF1 |
| D-myo-inositol (1,3,4)-trisphosphate biosynthesis | 2,36e00 | 3,16e-01 | 0,000 | INPP5A,INPP5J,ITPKB,MINPP1,SEC16A,SYNJ2 |
| Rhoa signaling | 2,35e00 | 1,64e-01 | -0,471 | ABL2,ACTC1,ARHGAP12,ARHGAP4,ARHGAP6,ARHGEF1,CDC42EP5,CIT,LPAR1,LPAR6,NEDD4,NGEF,PIP5K1A,PTK2,RAPGEF6,SEMA3F,SEPT2,SEPT6,SEPT8,WASF1 |
| Type i diabetes mellitus signaling | 2,29e00 | 1,68e-01 | -0,243 | APAF1,BID,CASP8,FAS,ICA1,IL1RAP,MAP3K14,MAP3K7,MAPK1,MAPK12,MAPK14,MAPK8,MYD88,NFKBID,NGFR,SOCS4,SOCS7,TNFRSF1B |
| Induction of apoptosis by hiv1 | 2,29e00 | 0,2 | 0,577 | APAF1,BAX,BID,CASP8,FAS,MAP3K14,MAPK12,MAPK8,NFKBID,NGFR,SLC25A13,TNFRSF1B |
| Inhibition of angiogenesis by tsp1 | 2,28e00 | 2,5e-01 | -0,707 | AKT1,AKT3,CD36,FYN,MAPK1,MAPK12,MAPK14,MAPK8 |
| Production of nitric oxide and reactive oxygen species in macrophages | 2,27e00 | 1,45e-01 | -0,392 | AKT1,AKT3,APOC1,CLU,FRS2,LYZ,MAP3K13,MAP3K14,MAP3K4,MAP3K6,MAP3K7,MAP3K8,MAPK1,MAPK12,MAPK14,MAPK8,NFKBID,NGFR,PIK3R3,PPP1R11,PPP1R7,PPP2R3B,PRKCH,PTPA,PTPN6,RAP1B,SERPINA1,TNFRSF1B |
| Wnt/β-catenin signaling | 2,2e00 | 1,48e-01 | -1,706 | ACVR1,AKT1,AKT3,AXIN2,CSNK1G1,CSNK2B,DKK2,GNAO1,LEF1,LRP6,MAP3K7,MAP4K1,MARK2,POU5F1,PPP2R3B,PTPA,RARA,RARB,SOX5,TCF4,TCF7L2,TGFB3,TGFBR3,TLE3,WNT5B |
| Corticotropin releasing hormone signaling | 2,17e00 | 1,56e-01 | -0,894 | ATF2,BDNF,CACNA1C,CACNA2D1,CACNA2D2,CACNA2D3,CACNB1,CACNB2,CACNG7,CREB3L4,CRHR2,GLI1,GNAO1,ITPR1,MAPK1,MAPK12,MAPK14,NPR3,PRKAR2A,PRKCH,RAP1B |
| Wnt/ca+ pathway | 2,17e00 | 1,94e-01 | -1,155 | ATF2,CAMK2A,CREB3L4,NFATC1,NFATC2,NOTUM,PLCB4,PLCD4,PLCH1,PLCL2,PPP3CA,WNT5B |
| Ampk signaling | 2,15e00 | 1,4e-01 | -0,626 | ACACA,AK4,AK5,AKT1,AKT3,ARID2,ATF2,CHRNB4,CPT1A,CPT1B,CPT1C,CREB3L4,DPF1,FRS2,GYS2,MAP3K7,MAPK1,MAPK12,MAPK14,MLST8,PFKFB1,PIK3R3,PPM1B,PPP2R3B,PRKAR2A,PTPA,RAB27A,SLC2A4,SMARCA2,TSC1 |
| Il-7 signaling pathway | 2,14e00 | 1,74e-01 | 0,000 | AKT1,AKT3,BAX,CDC25A,CDK2,EBF1,FRS2,FYN,MAPK1,MAPK12,MAPK14,MET,NFATC1,PIK3R3,PTK2 |
| Histamine degradation | 2,14e00 | 3,33e-01 | 0,000 | ALDH1A2,ALDH1L1,ALDH3A1,ALDH3B1,AOC1 |
| Adenosine nucleotides degradation ii | 2,14e00 | 3,33e-01 | 0,000 | ADA,ADA2,ADAL,NT5C3A,NT5M |
| Role of pkr in interferon induction and antiviral response | 2,11e00 | 2,2e-01 | 0,000 | AKT1,APAF1,ATF2,BID,CASP8,EIF2S1,MAP3K7,MAPK14,NFKBID |
| Pedf signaling | 2,1e00 | 1,72e-01 | 0,000 | AKT1,AKT3,BDNF,CASP8,FAS,FRS2,MAPK1,MAPK12,MAPK14,NFKBID,PIK3R3,PPARG,TCF4,TCF7L2,ZEB1 |
| Xenobiotic metabolism signaling | 2,07e00 | 1,32e-01 | 0,000 | ALDH1A2,ALDH1L1,ALDH3A1,ALDH3B1,CAMK2A,CAMK2B,CAMK2D,CAMK2G,CES3,CES4A,CHST15,CYP1A1,CYP3A5,FRS2,GSTM1,MAP3K13,MAP3K14,MAP3K4,MAP3K6,MAP3K7,MAP3K8,MAPK1,MAPK12,MAPK14,MAPK7,MAPK8,NCOR2,NDST2,NRIP1,PIK3R3,PPP2R3B,PRKCH,PTPA,SMOX,SULT4A1,UGT1A7 (INCLUDES OTHERS) |
| Antioxidant action of vitamin c | 2,07e00 | 1,63e-01 | 0,832 | CSF2RA,MAPK1,MAPK12,MAPK14,MAPK8,NFKBID,NOTUM,NXN,PAFAH1B2,PLCB4,PLCD4,PLCH1,PLCL2,PNPLA8,SLC2A14,SLC2A4,TXNRD2 |
| Cardiac β-adrenergic signaling | 2,03e00 | 1,51e-01 | -1,941 | AKAP12,AKAP2,AKAP6,AKAP7,AKAP9,ATP2A1,ATP2A3,CACNA1C,GNB4,PDE1A,PDE4A,PDE4C,PDE8A,PPP1R11,PPP1R7,PPP2R3B,PRKAR2A,PTPA,RYR2,SLC8A1,TDP2 |
| Atm signaling | 2,02e00 | 1,65e-01 | 0,258 | ATF2,BID,CDC25A,CDK2,CREB3L4,GADD45A,KAT5,MAPK12,MAPK14,MAPK8,MDM4,PPP2R3B,PTPA,RBBP8,SMC1B,ZEB1 |
| Apoptosis signaling | 2,01e00 | 1,69e-01 | 0,000 | ACIN1,APAF1,BAX,BID,CAPN1,CAPN3,CAPN8,CASP8,FAS,GAS2,MAP3K14,MAPK1,MAPK8,NFKBID,TNFRSF1B |
| Cdk5 signaling | 1,97e00 | 1,63e-01 | 0,000 | BDNF,CAPN1,FOSB,MAPK1,MAPK12,MAPK14,MAPK15,MAPK7,MAPK8,MAPT,NGFR,PPP1R11,PPP1R7,PPP2R3B,PRKAR2A,PTPA |
| Fak signaling | 1,97e00 | 1,63e-01 | 0,000 | ACTC1,AKT1,AKT3,ARHGAP26,ARHGEF7,CAPN1,CAPN3,CAPN8,FRS2,FYN,HMMR,ITGA4,MAPK1,PIK3R3,PTK2,PXN |
| Adenine and adenosine salvage iii | 1,96e00 | 0,5 | 0,000 | ADA,ADA2,ADAL |
| Purine ribonucleosides degradation to ribose-1-phosphate | 1,96e00 | 0,5 | 0,000 | ADA,ADA2,ADAL |
| Stat3 pathway | 1,95e00 | 1,76e-01 | 0,832 | CDC25A,DDR1,MAP3K21,MAPK1,MAPK12,MAPK14,MAPK8,NGFR,PTPN2,PTPN6,SOCS4,SOCS7,TGFBR3 |
| Germ cell-sertoli cell junction signaling | 1,92e00 | 1,42e-01 | 0,000 | ACTC1,ACTN4,AFDN,AKT1,CTNNA1,CTNNA2,FRS2,MAP3K13,MAP3K14,MAP3K4,MAP3K6,MAP3K7,MAP3K8,MAPK1,MAPK12,MAPK14,MAPK8,NECTIN3,PIK3R3,PLS1,PTK2,PXN,SORBS1,TGFB3 |
| Agrin interactions at neuromuscular junction | 1,9e00 | 1,79e-01 | 0,577 | ACTC1,ARHGEF7,ERBB3,ERBB4,GABPB1,ITGA4,ITGAL,MAPK1,MAPK12,MAPK8,PTK2,PXN |
| Reelin signaling in neurons | 1,88e00 | 1,63e-01 | 0,000 | AKT1,APP,ARHGEF1,ARHGEF4,FRS2,FYN,ITGA4,ITGAL,LRP8,MAP4K1,MAPK12,MAPK8,MAPT,PAFAH1B2,PIK3R3 |
| Aryl hydrocarbon receptor signaling | 1,84e00 | 1,47e-01 | 1,155 | ALDH1A2,ALDH1L1,ALDH3A1,ALDH3B1,APAF1,BAX,CDK2,CYP1A1,ESR2,FAS,GSTM1,HSPB7,MAPK1,MAPK8,NCOR2,NFIB,NRIP1,RARA,RARB,TGFB3 |
| Amyotrophic lateral sclerosis signaling | 1,84e00 | 1,55e-01 | 0,775 | AKT3,APAF1,BAX,BID,CACNA1C,CAPN1,CAPN3,CAPN8,CASP1,FRS2,GRIA2,GRIA3,GRIA4,PIK3R3,PPP3CA,RAB5C,RNF19A |
| Il-22 signaling | 1,82e00 | 2,5e-01 | -0,816 | AKT1,AKT3,MAPK1,MAPK12,MAPK14,MAPK8 |
| Tumoricidal function of hepatic natural killer cells | 1,82e00 | 2,5e-01 | 0,447 | APAF1,BAX,BID,CASP8,FAS,ITGAL |
| Gap junction signaling | 1,81e00 | 1,36e-01 | 0,000 | ACTC1,AKT1,AKT3,CSNK1G1,FRS2,GJB3,GRIA2,GRIA3,GRIA4,HTR2A,ITPR1,LPAR1,MAPK1,MAPK7,NOTUM,NPR3,PIK3R3,PLCB4,PLCD4,PLCH1,PLCL2,PPP3CA,PRKAR2A,PRKCH,PRKG1,TJP2 |
| Salvage pathways of pyrimidine ribonucleotides | 1,8e00 | 1,6e-01 | 0,000 | AK4,AK5,CDK2,CDK7,DAPK1,DMPK,HIPK1,MAK,MAP3K6,MAP3K8,MAPK1,MAPK7,MAPK8,NEK2,PRKCH |
| Glycogen degradation ii | 1,79e00 | 3,33e-01 | 0,000 | GDPGP1,PGM5,PYGL,TYMP |
| Purine nucleotides degradation ii (aerobic) | 1,78e00 | 2,78e-01 | 0,000 | ADA,ADA2,ADAL,NT5C3A,NT5M |
| D-myo-inositol (1,4,5)-trisphosphate degradation | 1,78e00 | 2,78e-01 | 0,000 | IMPA1,INPP5A,INPP5J,SEC16A,SYNJ2 |
| D-myo-inositol-5-phosphate metabolism | 1,77e00 | 1,41e-01 | 0,000 | ACP6,CDC25A,DUSP14,EYA4,MINPP1,NUDT9,PLCB4,PLCD4,PLCH1,PPFIA4,PPIP5K1,PPP1R7,PPP3CA,PRUNE1,PTPA,PTPN13,PTPN2,PTPN22,PTPN6,PTPRC,PXYLP1,STYXL1 |
| April mediated signaling | 1,75e00 | 2,05e-01 | 0,707 | MAP3K14,MAPK1,MAPK12,MAPK14,MAPK8,NFATC1,NFATC2,NFKBID |
| 4-1bb signaling in t lymphocytes | 1,73e00 | 2,19e-01 | 0,000 | ATF2,MAP3K14,MAPK1,MAPK12,MAPK14,MAPK8,NFKBID |
| 3-phosphoinositide biosynthesis | 1,73e00 | 1,34e-01 | 0,000 | ACP6,CDC25A,DUSP14,ERBB3,ERBB4,EYA4,FRS2,FYN,MET,MINPP1,NUDT9,PIK3R3,PIP5K1A,PPFIA4,PPIP5K1,PPP1R7,PPP3CA,PRUNE1,PTPA,PTPN13,PTPN2,PTPN22,PTPN6,PTPRC,PXYLP1,STYXL1 |
| Fcγriib signaling in b lymphocytes | 1,72e00 | 1,65e-01 | 0,816 | AKT1,BLNK,CACNA1C,CACNA2D1,CACNA2D2,CACNA2D3,CACNB1,CACNB2,CACNG7,FRS2,MAPK12,MAPK8,PIK3R3 |
| Regulation of cellular mechanics by calpain protease | 1,71e00 | 1,82e-01 | 0,707 | ACTN4,CAPN1,CAPN3,CAPN8,CAST,CDK2,ITGA4,MAPK1,PTK2,PXN |
| 1d-myo-inositol hexakisphosphate biosynthesis ii (mammalian) | 1,68e00 | 2,63e-01 | 0,000 | INPP5A,INPP5J,ITPKB,SEC16A,SYNJ2 |
| Putrescine degradation iii | 1,68e00 | 2,63e-01 | 0,000 | ALDH1A2,ALDH1L1,ALDH3A1,ALDH3B1,SMOX |
| Rar activation | 1,67e00 | 1,34e-01 | 0,000 | AKT1,AKT3,ALDH1A2,ARID2,CDK7,CRABP1,CSNK2B,DPF1,MAPK1,MAPK12,MAPK14,MAPK8,NCOR2,NRIP1,PIK3R3,PML,PRKAR2A,PRKCH,RARA,RARB,RDH10,SMAD9,SMARCA2,TGFB3,TNIP1 |
| Factors promoting cardiogenesis in vertebrates | 1,66e00 | 1,57e-01 | 0,000 | ACVR1,ATF2,CDK2,LEF1,LRP6,MAP3K7,MAPK14,NOX4,PRKCH,SMAD9,TCF4,TCF7L2,TGFB3,TGFBR3 |
| Dopamine-darpp32 feedback in camp signaling | 1,65e00 | 1,38e-01 | -1,147 | ATF2,ATP2A1,ATP2A3,CACNA1C,CAMKK1,CREB3L4,CREM,CSNK1G1,ITPR1,NOTUM,PLCB4,PLCD4,PLCH1,PLCL2,PPP1R11,PPP1R7,PPP2R3B,PPP3CA,PRKAR2A,PRKCH,PRKG1,PTPA |
| B cell activating factor signaling | 1,63e00 | 1,95e-01 | 0,378 | MAP3K14,MAPK1,MAPK12,MAPK14,MAPK8,NFATC1,NFATC2,NFKBID |
| Gm-csf signaling | 1,63e00 | 1,64e-01 | -0,632 | AKT1,AKT3,CAMK2A,CAMK2B,CAMK2D,CAMK2G,CSF2RA,FRS2,MAPK1,PIK3R3,PPP3CA,RUNX1 |
| Epithelial adherens junction signaling | 1,62e00 | 1,4e-01 | 0,000 | ACTC1,ACTN4,ACVR1,AFDN,AKT1,AKT3,CTNNA1,CTNNA2,LEF1,MAGI2,MET,MYH9,NECTIN3,RAP1B,RAPGEF1,SORBS1,TCF4,TCF7L2,TGFBR3,WASF1 |
| Huntington's disease signaling | 1,59e00 | 1,26e-01 | 0,000 | AKT1,AKT3,APAF1,ATF2,BAX,BDNF,CAPN1,CAPN3,CAPN8,CASP1,CASP8,CREB3L4,FRS2,GNB4,GOSR2,GPAA1,HDAC10,HDAC11,HDAC6,ITPR1,MAPK1,MAPK8,NAPB,NCOR2,NSF,PIK3R3,PLCB4,POLR2A,PRKCH,REST,STX16 |
| Lps/il-1 mediated inhibition of rxr function | 1,59e00 | 1,29e-01 | -0,577 | ACSL6,ALDH1A2,ALDH1L1,ALDH3A1,ALDH3B1,APOC1,CHST15,CPT1A,CPT1B,CPT1C,CYP2A6 (INCLUDES OTHERS),CYP3A5,FABP6,GSTM1,IL1RAP,IL1RN,MAP3K7,MAPK8,MYD88,NDST2,NGFR,NR1H3,RARA,SCARB1,SMOX,SULT4A1,TNFRSF1B |
| Tryptophan degradation x (mammalian, via tryptamine) | 1,59e00 | 2,5e-01 | 0,000 | ALDH1A2,ALDH1L1,ALDH3A1,ALDH3B1,SMOX |
| Flt3 signaling in hematopoietic progenitor cells | 1,56e00 | 1,57e-01 | -0,832 | AKT1,AKT3,ATF2,CREB3L4,FLT3LG,FRS2,GAB2,MAPK1,MAPK12,MAPK14,PIK3R3,RPS6KA2,STAT6 |
| Ephrin receptor signaling | 1,55e00 | 1,33e-01 | -1,414 | ABI1,AKT1,AKT3,ATF2,CREB3L4,EFNA1,EPHA3,EPHA4,FYN,GNAO1,GNB4,ITGA4,MAP3K14,MAPK1,NGEF,PTK2,PTPN13,PXN,RAP1B,RAPGEF1,RGS3,SORBS1,WIPF1 |
| Acute myeloid leukemia signaling | 1,55e00 | 1,52e-01 | -1,155 | AKT1,AKT3,CSF1R,CSF2RA,FLT3LG,FRS2,LEF1,MAPK1,PIK3R3,PML,RARA,RUNX1,TCF4,TCF7L2 |
| Glycogen degradation iii | 1,55e00 | 2,86e-01 | 0,000 | GDPGP1,PGM5,PYGL,TYMP |
| Il-6 signaling | 1,53e00 | 1,41e-01 | 0,000 | AKT1,AKT3,CSNK2B,FRS2,HSPB7,IL1RAP,IL1RN,IL6R,MAP3K14,MAP3K7,MAPK1,MAPK12,MAPK14,MAPK8,NFKBID,NGFR,PIK3R3,TNFRSF1B |
| Retinoic acid mediated apoptosis signaling | 1,51e00 | 1,69e-01 | -0,333 | APAF1,BID,CASP8,CRABP1,PARP8,RARA,RARB,TIPARP,TNFSF10,ZC3HAV1 |
| Ccr5 signaling in macrophages | 1,51e00 | 1,51e-01 | 0,447 | CACNA1C,CACNA2D1,CACNA2D2,CACNA2D3,CACNB1,CACNB2,CACNG7,FAS,GNB4,MAPK1,MAPK12,MAPK14,MAPK8,PRKCH |
| D-myo-inositol (1,4,5,6)-tetrakisphosphate biosynthesis | 1,5e00 | 1,38e-01 | 0,000 | ACP6,CDC25A,DUSP14,EYA4,MINPP1,NUDT9,PPFIA4,PPIP5K1,PPP1R7,PPP3CA,PRUNE1,PTPA,PTPN13,PTPN2,PTPN22,PTPN6,PTPRC,PXYLP1,STYXL1 |
| D-myo-inositol (3,4,5,6)-tetrakisphosphate biosynthesis | 1,5e00 | 1,38e-01 | 0,000 | ACP6,CDC25A,DUSP14,EYA4,MINPP1,NUDT9,PPFIA4,PPIP5K1,PPP1R7,PPP3CA,PRUNE1,PTPA,PTPN13,PTPN2,PTPN22,PTPN6,PTPRC,PXYLP1,STYXL1 |
| Sperm motility | 1,5e00 | 1,42e-01 | -0,728 | ITPR1,NOTUM,PAFAH1B2,PDE1A,PDE4A,PDE4C,PLCB4,PLCD4,PLCH1,PLCL2,PNPLA8,PRKAR2A,PRKCH,PRKG1,PTK2,TWF1,ZP3 |
| Ephrin a signaling | 1,47e00 | 1,67e-01 | 0,000 | EFNA1,EPHA3,EPHA4,FRS2,FYN,NGEF,NGFR,PIK3R3,PTK2,VAV3 |
| Camp-mediated signaling | 1,46e00 | 1,25e-01 | -1,706 | ADORA2A,AKAP12,AKAP2,AKAP6,AKAP7,AKAP9,ATF2,CAMK2A,CAMK2B,CAMK2D,CAMK2G,CREB3L4,CREM,FPR1,FSHR,GNAO1,GRM8,LPAR1,MAPK1,NPR3,PDE1A,PDE4A,PDE4C,PDE8A,PPP3CA,PRKAR2A,RGS10,TDP2 |
| Primary immunodeficiency signaling | 1,46e00 | 1,82e-01 | 0,000 | ADA,AIRE,BLNK,CD8A,DCLRE1C,PTPRC,RFXANK,TAP2 |
| Pparα/rxrα activation | 1,44e00 | 1,31e-01 | -1,000 | ACAA1,ACVR1,CD36,CPT1B,GK,GPD2,IL1RAP,MAP3K14,MAP3K7,MAPK1,MAPK14,MAPK8,NCOR2,NFKBID,NOTUM,PLCB4,PLCD4,PLCH1,PLCL2,PRKAR2A,TGFB3,TGFBR3 |
| Uva-induced mapk signaling | 1,41e00 | 1,43e-01 | 0,333 | FRS2,MAPK1,MAPK12,MAPK14,MAPK8,NOTUM,PARP8,PIK3R3,PLCB4,PLCD4,PLCH1,PLCL2,RPS6KA2,TIPARP,ZC3HAV1 |
| Myc mediated apoptosis signaling | 1,4e00 | 1,57e-01 | 0,000 | AKT1,AKT3,APAF1,BAX,BID,CASP8,FAS,FRS2,MAPK12,MAPK8,PIK3R3 |
| 2-ketoglutarate dehydrogenase complex | 1,39e00 | 0,5 | 0,000 | DLST,OGDH |
| Spermine and spermidine degradation i | 1,39e00 | 0,5 | 0,000 | PAOX,SMOX |
| Role of osteoblasts, osteoclasts and chondrocytes in rheumatoid arthritis | 1,38e00 | 1,23e-01 | 0,000 | ADAM17,AKT1,AKT3,CSF1R,DKK2,FRS2,IL1RAP,IL1RN,LEF1,LRP6,MAP3K14,MAP3K7,MAPK1,MAPK12,MAPK14,MAPK8,NFATC1,NFATC2,NFKBID,NGFR,PIK3R3,PPP3CA,RUNX2,SMAD9,TCF4,TCF7L2,TNFRSF1B,WNT5B |
| Dopamine degradation | 1,37e00 | 0,2 | 0,000 | ALDH1A2,ALDH1L1,ALDH3A1,ALDH3B1,SMOX,SULT4A1 |
| Role of brca1 in dna damage response | 1,35e00 | 1,5e-01 | 1,633 | ARID2,ATRIP,BABAM2,BARD1,DPF1,E2F5,FANCM,GADD45A,MLH1,RBBP8,SLC19A1,SMARCA2 |
| Granzyme b signaling | 1,35e00 | 2,5e-01 | 0,000 | APAF1,BID,CASP8,NUMA1 |
| Parkinson's signaling | 1,35e00 | 2,5e-01 | 0,000 | MAPK1,MAPK12,MAPK14,MAPK8 |
| Il-12 signaling and production in macrophages | 1,34e00 | 1,32e-01 | 0,000 | AKT1,AKT3,APOC1,CLU,FRS2,LYZ,MAP3K8,MAPK1,MAPK12,MAPK14,MAPK8,MYD88,PIK3R3,PPARG,PRKCH,SERPINA1,STAT6,TGFB3,ZNF668 |
| Gα12/13 signaling | 1,33e00 | 1,33e-01 | -0,728 | AKT1,AKT3,ARHGEF1,CDH17,CDH24,CDH4,FRS2,LPAR1,LPAR6,MAPK1,MAPK12,MAPK7,MAPK8,NFKBID,PIK3R3,PTK2,PXN,VAV3 |

**Supplemental Table S9. Complete list of significant (p-value ≤ 0,05) enriched canonical pathways of the differentially expressed genes using Ingenuity Pathway Analysis (IPA) software for high fat + insulin (HFIns) treatment.**

| HIGH FAT INSULIN TREATMENT |  |  |  |  |
| --- | --- | --- | --- | --- |
| Ingenuity canonical pathways | -log(p-value) | Ratio | z-score | Molecules |
| Rank signaling in osteoclasts | 4,68E00 | 2,2E-01 | -0,447 | FGFR1,FGFR4,FRS2,IKBKB,IKBKG,MAP2K7,MAP3K13,MAP3K4,MAP3K8,MAPK10,MAPK12,MAPK14,MITF,NFATC1,NFATC2,NFKBID,PIK3C2B,PIK3R3,PPP3CA,RELA,SRC,TNFRSF11A |
| Antioxidant action of vitamin c | 3,92E00 | 2,02E-01 | -1,886 | CSF2RB,IKBKB,IKBKG,MAPK10,MAPK12,MAPK14,NAPEPLD,NFKBID,PLCB3,PLCB4,PLCD4,PLCE1,PLCH2,PLCL1,PLCL2,PLD1,PNPLA8,RELA,SLC23A2,SLC2A14,TXNRD2 |
| Uva-induced mapk signaling | 3,86E00 | 0,2 | 1,291 | FGFR1,FGFR4,FRS2,MAPK10,MAPK12,MAPK14,PIK3C2B,PIK3R3,PLCB3,PLCB4,PLCD4,PLCE1,PLCH2,PLCL1,PLCL2,RPS6KA2,RPS6KA5,RPS6KB1,RRAS2,SMPD3,ZC3HAV1 |
| Il-6 signaling | 3,86E00 | 1,88E-01 | 0,000 | CSNK2B,FGFR1,FGFR4,FRS2,HSPB1,IKBKB,IKBKG,IL18,IL1R1,IL1RAP,IL1RL2,IL1RN,IL6R,MAP2K7,MAPK10,MAPK12,MAPK14,NFKBID,PIK3C2B,PIK3R3,RELA,RRAS2,TAB1,VEGFA |
| April mediated signaling | 3,61E00 | 2,82E-01 | -1,508 | IKBKB,IKBKG,MAP2K7,MAPK10,MAPK12,MAPK14,NFATC1,NFATC2,NFKBID,RELA,TNFRSF17 |
| Cd27 signaling in lymphocytes | 3,58E00 | 2,5E-01 | 0,000 | APAF1,BID,CASP8,IKBKB,IKBKG,MAP2K7,MAP3K13,MAP3K4,MAP3K8,MAPK10,MAPK12,NFKBID,RELA |
| Induction of apoptosis by hiv1 | 3,47E00 | 2,33E-01 | 0,000 | APAF1,BID,CASP8,DAXX,DIABLO,HTRA2,IKBKB,IKBKG,MAP2K7,MAPK10,MAPK12,NAIP,NFKBID,RELA |
| Xenobiotic metabolism signaling | 3,47E00 | 1,47E-01 | 0,000 | ALDH1L1,ALDH3A1,ALDH3A2,ALDH3B1,CES4A,CHST15,CYP1A1,CYP2B6,CYP3A7,EIF2AK3,FGFR1,FGFR4,FMO5,FRS2,GAL3ST2,GRIP1,GSTT2/GSTT2B,HS3ST4,MAP2K7,MAP3K13,MAP3K4,MAP3K8,MAPK12,MAPK14,MAPK7,NOS2,NRIP1,PIK3C2B,PIK3R3,PPARGC1A,PPP2R2B,PPP2R3B,PPP2R5C,PRKCH,PTPA,RELA,RRAS2,SULT1A1,SULT4A1,UGT1A7 (includes others) |
| Nf-κb signaling | 3,42E00 | 1,63E-01 | -0,186 | BTRC,CASP8,CSNK2B,FCER1G,FGFR1,FGFR4,FRS2,IKBKB,IKBKG,IL18,IL1R1,IL1RN,IRAK1,MAP2K7,MAP3K8,MYD88,NFKBID,NTRK1,NTRK2,PIK3C2B,PIK3R3,RELA,RRAS2,TAB1,TIRAP,TLR1,TNFRSF11A,TNFRSF17,UBE2V1 |
| B cell activating factor signaling | 3,4E00 | 2,68E-01 | -1,265 | IKBKB,IKBKG,MAP2K7,MAPK10,MAPK12,MAPK14,NFATC1,NFATC2,NFKBID,RELA,TNFRSF17 |
| Role of macrophages, fibroblasts and endothelial cells in rheumatoid arthritis | 3,38E00 | 1,42E-01 | 0,000 | APC2,CSF1,FGFR1,FGFR4,FRS2,FZD3,GNAO1,IKBKB,IKBKG,IL18,IL1R1,IL1RAP,IL1RL2,IL1RN,IL32,IL6R,IRAK1,LEF1,MAP2K7,MAPK14,MYD88,NFATC1,NFATC2,NFKBID,NOS2,PIK3C2B,PIK3R3,PLCB3,PLCB4,PLCD4,PLCE1,PLCH2,PLCL1,PLCL2,PPP3CA,PRKCH,PRSS3,RELA,RRAS2,SRC,TCF7L2,TLR1,VEGFA |
| Type i diabetes mellitus signaling | 3,3E00 | 1,87E-01 | -2,357 | APAF1,BID,CASP8,CD247,FCER1G,ICA1,IKBKB,IKBKG,IL1R1,IL1RAP,IRAK1,MAP2K7,MAPK10,MAPK12,MAPK14,MYD88,NFKBID,NOS2,RELA,SOCS4 |
| Role of nfat in cardiac hypertrophy | 3,29E00 | 1,53E-01 | 2,121 | CABIN1,CACNA1C,CACNA1D,CACNA2D1,CACNB1,CACNG7,FGFR1,FGFR4,FRS2,GATA4,GNAS,HDAC10,HDAC6,IGF1,MAP2K7,MAPK10,MAPK12,MAPK14,PIK3C2B,PIK3R3,PLCB3,PLCB4,PLCD4,PLCE1,PLCH2,PLCL1,PLCL2,PPP3CA,PRKCH,RCAN1,RCAN3,RRAS2,SRC |
| Il-4 signaling | 3,18E00 | 1,98E-01 | 0,000 | FGFR1,FGFR4,FRS2,IL13RA1,INPP5J,NFATC1,NFATC2,NR3C1,NR3C2,PIK3C2B,PIK3R3,PTPN6,RPS6KB1,RRAS2,SYNJ1,SYNJ2,TYK2 |
| Calcium transport i | 3,15E00 | 0,5 | 0,000 | ATP2A1,ATP2B1,ATP2B3,ATP2B4,ATP2C1 |
| Inos signaling | 3,11E00 | 2,5E-01 | -0,905 | IKBKB,IKBKG,IRAK1,MAPK12,MAPK14,MYD88,NFKBID,NOS2,RELA,TAB1,TYK2 |
| Endothelin-1 signaling | 3,11E00 | 1,56E-01 | 1,300 | CASP4,CASP8,EDNRA,FGFR1,FGFR4,FRS2,GNAO1,GNAS,GUCY1B1,MAPK10,MAPK12,MAPK14,MAPK7,NAPEPLD,NOS2,PIK3C2B,PIK3R3,PLCB3,PLCB4,PLCD4,PLCE1,PLCH2,PLCL1,PLCL2,PLD1,PNPLA8,PRKCH,RRAS2,SRC |
| Pkcθ signaling in t lymphocytes | 3,01E00 | 1,62E-01 | -1,147 | CACNA1C,CACNA1D,CACNA2D1,CACNB1,CACNG7,CD247,FCER1G,FGFR1,FGFR4,FRS2,IKBKB,IKBKG,LAT,MAP3K13,MAP3K4,MAP3K8,NFATC1,NFATC2,NFKBID,PIK3C2B,PIK3R3,PPP3CA,RELA,RRAS2,VAV2 |
| Stat3 pathway | 2,99E00 | 2,03E-01 | -0,258 | CDC25A,FGFR1,FGFR4,MAP3K21,MAPK10,MAPK12,MAPK14,NTRK1,NTRK2,PTPN6,RRAS2,SOCS4,SRC,TNFRSF11A,TYK2 |
| Rhoa signaling | 2,95E00 | 1,72E-01 | 1,606 | ACTR2,ARHGAP12,ARHGAP9,CIT,IGF1,KTN1,LPAR2,LPAR3,MYLK2,NRP2,PIP5K1A,PIP5K1C,PIP5KL1,PLD1,PPP1R12A,PPP1R12B,RND3,SEPT3,SEPT4,SEPT6,SEPT9 |
| P70s6k signaling | 2,92E00 | 1,68E-01 | 1,706 | FGFR1,FGFR4,FRS2,MAPT,PIK3C2B,PIK3R3,PLCB3,PLCB4,PLCD4,PLCE1,PLCH2,PLCL1,PLCL2,PLD1,PPP2R2B,PPP2R3B,PPP2R5C,PRKCH,PTPA,RPS6KB1,RRAS2,SRC |
| D-myo-inositol (1,4,5)-trisphosphate biosynthesis | 2,91E00 | 2,96E-01 | 0,000 | PIP5K1A,PIP5K1C,PIP5KL1,PLCB3,PLCB4,PLCD4,PLCE1,PLCH2 |
| Production of nitric oxide and reactive oxygen species in macrophages | 2,85E00 | 1,5E-01 | -1,134 | APOA1,APOL1,FGFR1,FGFR4,FRS2,IKBKB,IKBKG,MAP2K7,MAP3K13,MAP3K4,MAP3K8,MAPK10,MAPK12,MAPK14,NFKBID,NOS2,PIK3C2B,PIK3R3,PPP1R12A,PPP1R7,PPP2R2B,PPP2R3B,PPP2R5C,PRKCH,PTPA,PTPN6,RELA,RND3,TYK2 |
| Tweak signaling | 2,83E00 | 2,65E-01 | -0,333 | APAF1,BID,CASP8,IKBKB,IKBKG,NAIP,NFKBID,RELA,TNFRSF25 |
| Wnt/ca+ pathway | 2,8E00 | 2,1E-01 | 0,277 | FZD3,NFATC1,NFATC2,PDE6G,PLCB3,PLCB4,PLCD4,PLCE1,PLCH2,PLCL1,PLCL2,PPP3CA,RELA |
| Synaptic long term depression | 2,8E00 | 1,55E-01 | 1,569 | CACNA1C,CACNA1D,CACNA2D1,CACNB1,CACNG7,GNAO1,GNAS,GRID2,GRM5,GUCY1B1,IGF1,NOS2,PLCB3,PLCB4,PLCD4,PLCE1,PLCH2,PLCL1,PLCL2,PNPLA8,PPP2R2B,PPP2R3B,PPP2R5C,PRKCH,PTPA,RRAS2 |
| Acute phase response signaling | 2,76E00 | 1,54E-01 | -0,447 | APOA1,C1S,C4BPB,ECSIT,HNRNPK,IKBKB,IKBKG,IL18,IL1R1,IL1RAP,IL1RN,IL6R,IRAK1,KLKB1,MAP2K7,MAPK12,MAPK14,MYD88,NFKBID,NR3C1,PIK3R3,RELA,RRAS2,SERPIND1,SOCS4,TAB1 |
| Amyotrophic lateral sclerosis signaling | 2,74E00 | 1,73E-01 | -0,500 | APAF1,BID,CACNA1C,CACNA1D,CAPN10,CAPN3,FGFR1,FGFR4,FRS2,GRID2,GRIK2,GRIK5,GRIN2A,IGF1,NAIP,PIK3C2B,PIK3R3,PPP3CA,VEGFA |
| Hippo signaling | 2,74E00 | 1,86E-01 | 0,000 | AMOT,BTRC,DLG2,DLG3,FRMD6,LATS1,NF2,PPP1R12A,PPP1R7,PPP2R2B,PPP2R3B,PPP2R5C,PTPA,SMAD3,STK4,TAZ |
| Cardiac hypertrophy signaling | 2,72E00 | 1,42E-01 | 1,061 | CACNA1C,CACNA1D,EIF2B4,FGFR1,FGFR4,FRS2,GATA4,GNAO1,GNAS,HSPB1,IGF1,IL6R,MAP2K7,MAP3K13,MAP3K4,MAP3K8,MAPK10,MAPK12,MAPK14,PIK3C2B,PIK3R3,PLCB3,PLCB4,PLCD4,PLCE1,PLCH2,PLCL1,PLCL2,PPP3CA,RND3,RPS6KB1,RRAS2,TAB1 |
| Regulation of il-2 expression in activated and anergic t lymphocytes | 2,69E00 | 1,9E-01 | 0,000 | CD247,IKBKB,IKBKG,LAT,MAP2K7,MAPK10,MAPK12,NFATC1,NFATC2,NFKBID,PPP3CA,RELA,RRAS2,SMAD3,VAV2 |
| Calcium signaling | 2,68E00 | 1,46E-01 | 0,775 | ASPH,ATP2A1,ATP2B1,ATP2B3,ATP2B4,ATP2C1,CABIN1,CACNA1C,CACNA1D,CACNA2D1,CACNB1,CACNG7,CAMKK2,CHRFAM7A,CHRNA3,CHRNB4,GRIN2A,HDAC10,HDAC6,MCU,MYH10,MYH7B,NFATC1,NFATC2,PPP3CA,RCAN1,RCAN3,TNNC2,TRPC3 |
| Ngf signaling | 2,66E00 | 1,67E-01 | -0,447 | FGFR1,FGFR4,FRS2,IKBKB,IKBKG,MAP3K13,MAP3K4,MAP3K8,MAPK10,MAPK12,MAPK7,NTRK1,PIK3C2B,PIK3R3,RELA,RPS6KA2,RPS6KA5,RPS6KB1,RRAS2,SMPD3 |
| Phenylethylamine degradation i | 2,66E00 | 7,5E-01 | 0,000 | ALDH3A2,AOC2,AOC3 |
| Role of osteoblasts, osteoclasts and chondrocytes in rheumatoid arthritis | 2,59E00 | 1,4E-01 | 0,000 | ADAM17,APC2,CSF1,FGFR1,FGFR4,FRS2,FZD3,IGF1,IKBKB,IKBKG,IL18,IL1R1,IL1RAP,IL1RL2,IL1RN,LEF1,MAP2K7,MAPK10,MAPK12,MAPK14,NAIP,NFATC1,NFATC2,NFKBID,PIK3C2B,PIK3R3,PPP3CA,RELA,SPP1,SRC,TCF7L2,TNFRSF11A |
| Thrombin signaling | 2,58E00 | 1,44E-01 | 1,890 | ARHGEF16,ARHGEF9,FGFR1,FGFR4,FRS2,GATA2,GATA4,GNAO1,GNAS,IKBKB,MAPK12,MAPK14,PIK3C2B,PIK3R3,PLCB3,PLCB4,PLCD4,PLCE1,PLCH2,PLCL1,PLCL2,PPP1R12A,PPP1R12B,PRKCH,RELA,RND3,RPS6KB1,RRAS2,SRC |
| Neuropathic pain signaling in dorsal horn neurons | 2,56E00 | 1,67E-01 | 1,606 | FGFR1,FGFR4,FRS2,GRIN2A,GRM5,KCNN2,NTRK2,PIK3C2B,PIK3R3,PLCB3,PLCB4,PLCD4,PLCE1,PLCH2,PLCL1,PLCL2,PRKCH,SRC,TACR1 |
| Notch signaling | 2,55E00 | 2,43E-01 | -0,447 | ADAM17,CNTN1,DLL3,DTX1,FURIN,HES7,MAML1,NUMB,RBPJ |
| Ctla4 signaling in cytotoxic t lymphocytes | 2,53E00 | 1,73E-01 | 0,000 | AP1B1,AP1S2,AP2B1,CD247,FCER1G,FGFR1,FGFR4,FRS2,LAT,PIK3C2B,PIK3R3,PPP2R2B,PPP2R3B,PPP2R5C,PTPA,PTPN22,PTPN6 |
| Dendritic cell maturation | 2,51E00 | 1,46E-01 | 1,000 | COL10A1,FCER1G,FGFR1,FGFR4,FRS2,IKBKB,IKBKG,IL18,IL1RL2,IL1RN,IL32,MAPK10,MAPK12,MAPK14,MYD88,NFKBID,PIK3C2B,PIK3R3,PLCB3,PLCB4,PLCD4,PLCE1,PLCH2,PLCL1,PLCL2,RELA,TAB1 |
| Dopamine-darpp32 feedback in camp signaling | 2,45E00 | 1,5E-01 | 1,706 | ATP2A1,CACNA1C,CACNA1D,CAMKK2,CREM,CSNK1G1,GNAS,GRIN2A,GUCY1B1,PLCB3,PLCB4,PLCD4,PLCE1,PLCH2,PLCL1,PLCL2,PPP1R12A,PPP1R7,PPP2R2B,PPP2R3B,PPP2R5C,PPP3CA,PRKCH,PTPA |
| Il-1 signaling | 2,43E00 | 1,74E-01 | 0,000 | ECSIT,GNAO1,GNAS,IKBKB,IKBKG,IL1R1,IL1RAP,IRAK1,MAP2K7,MAPK10,MAPK12,MAPK14,MYD88,NFKBID,RELA,TAB1 |
| Death receptor signaling | 2,43E00 | 1,74E-01 | 0,500 | APAF1,BID,CASP8,DAXX,DIABLO,HSPB1,HTRA2,IKBKB,IKBKG,MAP2K7,NAIP,NFKBID,RELA,TNFRSF10A,TNFRSF25,ZC3HAV1 |
| B cell receptor signaling | 2,41E00 | 1,44E-01 | 0,000 | EBF1,FGFR1,FGFR4,FRS2,IKBKB,IKBKG,INPP5J,MAP2K7,MAP3K13,MAP3K4,MAP3K8,MAPK12,MAPK14,NFATC1,NFATC2,NFKBID,PIK3C2B,PIK3R3,PPP3CA,PTPN6,RASSF5,RELA,RPS6KB1,RRAS2,SYNJ1,SYNJ2,VAV2 |
| Cellular effects of sildenafil (viagra) | 2,41E00 | 1,59E-01 | 0,000 | CACNA1C,CACNA1D,CACNG7,GNAS,GUCY1B1,KCNN2,MYH10,MYH7B,PDE4A,PDE4B,PLCB3,PLCB4,PLCD4,PLCE1,PLCH2,PLCL1,PLCL2,PPP1R12A,PPP1R12B,SLC4A11 |
| Il-10 signaling | 2,37E00 | 1,88E-01 | 0,000 | IKBKB,IKBKG,IL18,IL1R1,IL1RAP,IL1RL2,IL1RN,MAPK12,MAPK14,NFKBID,RELA,TAB1,TYK2 |
| Pten signaling | 2,35E00 | 1,6E-01 | 0,943 | CSNK2B,FGFR1,FGFR4,FOXO3,IKBKB,IKBKG,INPP5J,ITGA4,MAGIX,MCRS1,NTRK1,NTRK2,PIK3R3,RELA,RPS6KB1,RRAS2,SYNJ1,SYNJ2,TNFRSF11A |
| Lps/il-1 mediated inhibition of rxr function | 2,33E00 | 1,39E-01 | 0,000 | ABCG1,ACSL6,ALDH1L1,ALDH3A1,ALDH3A2,ALDH3B1,CETP,CHST15,CYP2B6,CYP3A7,ECSIT,FMO5,GAL3ST2,GSTT2/GSTT2B,HS3ST4,IL18,IL1R1,IL1RAP,IL1RL2,IL1RN,IRAK1,MAP2K7,MYD88,NR1H2,NR1H3,NR5A2,PPARGC1A,SULT1A1,SULT4A1 |
| Superpathway of inositol phosphate compounds | 2,32E00 | 1,36E-01 | 0,000 | ACP6,CDC25A,FGFR1,FGFR4,FRS2,ILKAP,INPP5J,IP6K3,PIK3C2B,PIK3R3,PIP5K1A,PIP5K1C,PIP5KL1,PLCB3,PLCB4,PLCD4,PLCE1,PLCH2,PPM1K,PPP1R12A,PPP1R7,PPP3CA,PPP4C,PTPA,PTPN22,PTPN6,STYX,STYXL1,SYNJ1,SYNJ2,TPTE |
| Il-17a signaling in airway cells | 2,32E00 | 1,79E-01 | -0,277 | FGFR1,FGFR4,FRS2,IKBKB,IKBKG,IL19,MAPK10,MAPK12,MAPK14,NFKBID,PIK3C2B,PIK3R3,RELA,TYK2 |
| Pedf signaling | 2,27E00 | 1,72E-01 | -1,069 | CASP8,FGFR1,FGFR4,FRS2,IKBKB,IKBKG,MAPK12,MAPK14,NFKBID,PIK3C2B,PIK3R3,PPARG,RELA,RRAS2,TCF7L2 |
| Aldosterone signaling in epithelial cells | 2,21E00 | 1,44E-01 | 1,414 | DNAJC22,DNAJC6,FGFR1,FGFR4,FRS2,HSPA4L,HSPA8,HSPB1,NR3C2,PIK3C2B,PIK3R3,PIP5K1A,PIP5K1C,PIP5KL1,PLCB3,PLCB4,PLCD4,PLCE1,PLCH2,PLCL1,PLCL2,PRKCH,SACS,SGK1 |
| Molecular mechanisms of cancer | 2,2E00 | 1,21E-01 | 0,000 | APAF1,ARHGEF16,ARHGEF18,ARHGEF7,ARHGEF9,AURKA,BID,CASP8,CDC25A,CDK14,CDK15,CDK20,CTNNA1,DAXX,DIABLO,E2F5,E2F8,FGFR1,FGFR4,FRS2,FZD3,GNAO1,GNAS,IKBKG,ITGA4,LEF1,MAPK10,MAPK12,MAPK14,MAX,NAIP,NFKBID,PIK3C2B,PIK3R3,PLCB3,PLCB4,PMAIP1,PRKCH,RAPGEF1,RBPJ,RELA,RND3,RRAS2,SMAD3,SRC,TAB1,TYK2 |
| Atm signaling | 2,2E00 | 1,65E-01 | -1,291 | BID,CCNB1,CCNB3,CDC25A,MAPK10,MAPK12,MAPK14,MDM4,PPP2R2B,PPP2R3B,PPP2R5C,PTPA,RAD17,RAD50,RBBP8,TP73 |
| Pparα/rxrα activation | 2,18E00 | 1,43E-01 | 0,500 | ACAA1,APOA1,GK,GNAS,GPD1,IKBKB,IKBKG,IL1R1,IL1RAP,IL1RL2,MAP2K7,MAPK14,NFKBID,PLCB3,PLCB4,PLCD4,PLCE1,PLCH2,PLCL1,PLCL2,PPARGC1A,RELA,RRAS2,SMAD3 |
| Gαq signaling | 2,18E00 | 1,45E-01 | 0,426 | AVPR1B,FGFR1,FGFR4,FRS2,GNAS,GRM5,HTR2A,IKBKB,IKBKG,NAPEPLD,NFATC1,NFATC2,NFKBID,PIK3C2B,PIK3R3,PLCB3,PLCB4,PLD1,PPP3CA,PRKCH,RELA,RGS7,RND3 |
| Glycine betaine degradation | 2,17E00 | 0,4 | 0,000 | SARDH,SDSL,SHMT1,SHMT2 |
| Role of chk proteins in cell cycle checkpoint control | 2,17E00 | 1,93E-01 | 0,378 | CDC25A,CLSPN,E2F5,E2F8,PPP2R2B,PPP2R3B,PPP2R5C,PTPA,RAD17,RAD50,SLC19A1 |
| Glycine biosynthesis i | 2,16E00 | 1 | 0,000 | SHMT1,SHMT2 |
| Sphingosine-1-phosphate signaling | 2,15E00 | 1,53E-01 | 1,698 | ACER1,ASAH1,CASP4,CASP8,FGFR1,FGFR4,FRS2,PIK3C2B,PIK3R3,PLCB3,PLCB4,PLCD4,PLCE1,PLCH2,PLCL1,PLCL2,RND3,SMPD3,SPHK1 |
| Clathrin-mediated endocytosis signaling | 2,11E00 | 1,36E-01 | 0,000 | ACTR2,AP1B1,AP2B1,APOA1,APOL1,CSNK2B,CTTN,DNM1L,EPS15,FGF1,FGF17,FGFR1,FGFR4,FRS2,HSPA8,IGF1,LDLR,NUMB,PIK3C2B,PIK3R3,PIP5K1C,PPP3CA,RAB7A,SH3GLB2,SRC,SYNJ1,VEGFA |
| Type ii diabetes mellitus signaling | 2,11E00 | 1,45E-01 | 0,535 | ACSL6,CACNA1C,CACNA1D,CACNA2D1,CACNB1,CACNG7,FGFR1,FGFR4,FRS2,IKBKB,IKBKG,MAP2K7,MAPK10,MAPK12,NFKBID,PIK3C2B,PIK3R3,PPARG,PRKCH,RELA,SMPD3,SOCS4 |
| Pi3k signaling in b lymphocytes | 2,08E00 | 1,51E-01 | 0,471 | ATF6B,ATF7,FOXO3,IKBKB,IKBKG,NFATC1,NFATC2,NFKBID,PLCB3,PLCB4,PLCD4,PLCE1,PLCH2,PLCL1,PLCL2,PPP3CA,RELA,RRAS2,VAV2 |
| Gα12/13 signaling | 2,07E00 | 1,48E-01 | 1,147 | CDH24,CDH7,FGFR1,FGFR4,FRS2,IKBKB,IKBKG,LPAR2,LPAR3,MAP2K7,MAPK10,MAPK12,MAPK7,NFKBID,PIK3C2B,PIK3R3,RELA,RRAS2,SRC,VAV2 |
| Role of nfat in regulation of the immune response | 2,06E00 | 1,38E-01 | 0,853 | CABIN1,CD247,CSNK1G1,FCER1G,FGFR1,FGFR4,FRS2,GATA4,GNAO1,GNAS,IKBKB,IKBKG,LAT,NFATC1,NFATC2,NFKBID,PIK3C2B,PIK3R3,PLCB3,PLCB4,PPP3CA,RCAN1,RCAN3,RELA,RRAS2 |
| Toll-like receptor signaling | 2,06E00 | 1,73E-01 | 0,000 | ECSIT,IKBKB,IKBKG,IL18,IL1RN,IRAK1,MAPK12,MAPK14,MYD88,RELA,TAB1,TIRAP,TLR1 |
| Reelin signaling in neurons | 2,05E00 | 1,63E-01 | 0,000 | ARHGEF16,ARHGEF9,FGFR1,FGFR4,FRS2,ITGA4,ITGAL,LRP8,MAP2K7,MAPK10,MAPK12,MAPT,PIK3C2B,PIK3R3,SRC |
| Acute myeloid leukemia signaling | 2,05E00 | 1,63E-01 | -0,832 | CSF2RB,FGFR1,FGFR4,FLT3LG,FRS2,IDH3B,IDH3G,LEF1,MAP2K7,PIK3C2B,PIK3R3,RELA,RPS6KB1,RRAS2,TCF7L2 |
| Ppar signaling | 2,05E00 | 1,63E-01 | 0,258 | IKBKB,IKBKG,IL18,IL1R1,IL1RAP,IL1RL2,IL1RN,NFKBID,NR1H3,NRIP1,PPARG,PPARGC1A,RELA,RRAS2,TAB1 |
| Cd28 signaling in t helper cells | 2,04E00 | 1,5E-01 | 0,000 | ACTR2,CD247,FCER1G,FGFR1,FGFR4,FRS2,IKBKB,IKBKG,LAT,MAPK10,MAPK12,NFATC1,NFATC2,NFKBID,PIK3C2B,PIK3R3,PPP3CA,PTPN6,RELA |
| Creb signaling in neurons | 2,04E00 | 1,33E-01 | 1,789 | CACNA1C,CACNA1D,CACNA2D1,CACNB1,CACNG7,FGFR1,FGFR4,FRS2,GNAO1,GNAS,GRID2,GRIK2,GRIK5,GRIN2A,GRM5,PIK3C2B,PIK3R3,PLCB3,PLCB4,PLCD4,PLCE1,PLCH2,PLCL1,PLCL2,POLR2A,POLR2J2/POLR2J3,PRKCH,RRAS2 |
| Ceramide signaling | 2 | 1,61E-01 | -0,832 | CNKSR1,DIABLO,FGFR1,FGFR4,FRS2,PIK3C2B,PIK3R3,PPP2R2B,PPP2R3B,PPP2R5C,PTPA,RELA,RRAS2,SMPD3,SPHK1 |
| Small cell lung cancer signaling | 1,98E00 | 1,65E-01 | 0,632 | APAF1,BID,FGFR1,FGFR4,FRS2,IKBKB,IKBKG,MAX,NFKBID,PIK3C2B,PIK3R3,RARB,RELA,RXRB |
| Sperm motility | 1,97E00 | 1,5E-01 | 0,471 | GNAS,GUCY1B1,LTK,PDE4A,PDE4B,PLCB3,PLCB4,PLCD4,PLCE1,PLCH2,PLCL1,PLCL2,PNPLA8,PRKCH,PTK7,TWF1,TXK,ZP3 |
| Signaling by rho family gtpases | 1,97E00 | 1,27E-01 | 0,898 | ACTR2,ARHGEF16,ARHGEF18,ARHGEF7,ARHGEF9,CDH24,CDH7,CIT,FGFR1,FGFR4,FRS2,GNAO1,GNAS,ITGA4,MAP2K7,MAP3K21,MAPK10,MAPK12,PIK3C2B,PIK3R3,PIP5K1A,PIP5K1C,PIP5KL1,PLD1,PPP1R12A,PPP1R12B,RELA,RND3,SEPT3,SEPT4,SEPT6,SEPT9 |
| Role of il-17a in arthritis | 1,95E00 | 1,74E-01 | 0,000 | FGFR1,FGFR4,FRS2,IKBKG,MAPK10,MAPK12,MAPK14,NFKBID,NOS2,PIK3C2B,PIK3R3,RELA |
| Sapk/jnk signaling | 1,95E00 | 1,55E-01 | -0,500 | DAXX,FCER1G,FGFR1,FGFR4,FRS2,HNRNPK,MAP2K7,MAP3K13,MAP3K4,MAPK10,MAPK12,NFATC1,PIK3C2B,PIK3R3,RRAS2,TAB1 |
| Cardiac β-adrenergic signaling | 1,94E00 | 1,44E-01 | 0,471 | AKAP14,AKAP9,ATP2A1,CACNA1C,CACNA1D,GNAS,MPPE1,NAPEPLD,PDE4A,PDE4B,PDE6G,PDE8A,PKIB,PKIG,PPP1R12A,PPP1R7,PPP2R2B,PPP2R3B,PPP2R5C,PTPA |
| 14-3-3-mediated signaling | 1,94E00 | 1,46E-01 | 0,943 | FGFR1,FGFR4,FRS2,MAPK10,MAPK12,MAPT,PIK3C2B,PIK3R3,PLCB3,PLCB4,PLCD4,PLCE1,PLCH2,PLCL1,PLCL2,PRKCH,RRAS2,SRC,TP73 |
| Hepatic cholestasis | 1,92E00 | 1,39E-01 | 0,000 | CETP,ESR1,FGFR4,IKBKB,IKBKG,IL17B,IL18,IL1R1,IL1RAP,IL1RL2,IL1RN,IRAK1,MAP3K4,MAPK10,MAPK12,MYD88,NFKBID,NR1H3,NR5A2,PRKCH,RELA,TIRAP |
| Cd40 signaling | 1,92E00 | 1,67E-01 | 0,277 | FGFR1,FGFR4,FRS2,IKBKB,IKBKG,MAP2K7,MAPK10,MAPK12,MAPK14,NFKBID,PIK3C2B,PIK3R3,RELA |
| Bupropion degradation | 1,91E00 | 2,5E-01 | 0,000 | CYP1A1,CYP2B6,CYP2J2,CYP2U1,CYP3A7,CYP4X1 |
| Myc mediated apoptosis signaling | 1,9E00 | 1,71E-01 | 0,000 | APAF1,BID,CASP8,FGFR1,FGFR4,FRS2,IGF1,MAPK10,MAPK12,PIK3C2B,PIK3R3,RRAS2 |
| Lps-stimulated mapk signaling | 1,9E00 | 1,61E-01 | -0,535 | FGFR1,FGFR4,FRS2,IKBKB,IKBKG,MAPK10,MAPK12,MAPK14,NFKBID,PIK3C2B,PIK3R3,PRKCH,RELA,RRAS2 |
| Fcγriib signaling in b lymphocytes | 1,87E00 | 1,65E-01 | 0,000 | CACNA1C,CACNA1D,CACNA2D1,CACNB1,CACNG7,FGFR1,FGFR4,FRS2,MAPK10,MAPK12,PIK3C2B,PIK3R3,RRAS2 |
| Pi3k/akt signaling | 1,87E00 | 1,46E-01 | -1,213 | FOXO3,IKBKB,IKBKG,INPP5J,ITGA4,MAP3K8,NFKBID,PIK3R3,PPP2R2B,PPP2R3B,PPP2R5C,PTPA,RELA,RPS6KB1,RRAS2,SYNJ1,SYNJ2,TYK2 |
| Fatty acid α-oxidation | 1,86E00 | 2,78E-01 | 0,000 | ALDH1L1,ALDH3A1,ALDH3A2,ALDH3B1,ALOXE3 |
| Mitotic roles of polo-like kinase | 1,84E00 | 1,75E-01 | 0,447 | CCNB1,CCNB3,CDC25A,FZR1,KIF23,PPP2R2B,PPP2R3B,PPP2R5C,PTPA,RAD21,STAG2 |
| Opioid signaling pathway | 1,84E00 | 1,27E-01 | 0,756 | AP1B1,AP2B1,CACNA1C,CACNA1D,CACNA2D1,CACNB1,CACNG7,FOSB,GNAO1,GNAS,GRIN2A,GRK4,MAP2K7,MAPK12,MAPK7,OPRK1,PPP3CA,PRKCH,RGS12,RGS19,RGS20,RGS3,RGS5,RGS7,RPS6KA2,RPS6KA5,RPS6KB1,RRAS2,SLC12A5,SRC |
| 4-1bb signaling in t lymphocytes | 1,83E00 | 2,19E-01 | -0,816 | IKBKB,IKBKG,MAPK10,MAPK12,MAPK14,NFKBID,RELA |
| Glioblastoma multiforme signaling | 1,83E00 | 1,37E-01 | 0,447 | E2F5,E2F8,FGFR1,FGFR4,FRS2,FZD3,IGF1,LEF1,NF2,PIK3C2B,PIK3R3,PLCB3,PLCB4,PLCD4,PLCE1,PLCH2,PLCL1,PLCL2,RND3,RPS6KB1,RRAS2,SRC |
| Apoptosis signaling | 1,82E00 | 1,57E-01 | 0,000 | APAF1,BID,CAPN10,CAPN3,CASP8,DIABLO,HTRA2,IKBKB,IKBKG,MAP2K7,NAIP,NFKBID,RELA,RRAS2 |
| Cdk5 signaling | 1,8E00 | 1,53E-01 | 0,775 | FOSB,GNAS,MAPK10,MAPK12,MAPK14,MAPK7,MAPT,NTRK2,PPP1R12A,PPP1R7,PPP2R2B,PPP2R3B,PPP2R5C,PTPA,RRAS2 |
| Gnrh signaling | 1,8E00 | 1,36E-01 | -0,243 | CACNA1C,CACNA1D,CACNA2D1,CACNB1,CACNG7,GNAS,GNRH1,MAP2K7,MAP3K13,MAP3K4,MAP3K8,MAPK10,MAPK12,MAPK14,MAPK7,MMP2,PLCB3,PLCB4,PRKCH,RELA,RRAS2,SRC |
| Endometrial cancer signaling | 1,79E00 | 1,72E-01 | 0,707 | APC2,CTNNA1,FGFR1,FGFR4,FOXO3,FRS2,LEF1,MLH1,PIK3C2B,PIK3R3,RRAS2 |
| Erythropoietin signaling | 1,79E00 | 1,6E-01 | 0,000 | FGFR1,FGFR4,FRS2,IKBKG,NFKBID,PIK3C2B,PIK3R3,PRKCH,PTPN6,RELA,RPS6KB1,RRAS2,SRC |
| Tnfr1 signaling | 1,78E00 | 1,88E-01 | -1,000 | APAF1,BID,CASP8,IKBKB,IKBKG,MADD,NAIP,NFKBID,RELA |
| Melatonin degradation i | 1,78E00 | 1,79E-01 | 0,000 | CSGALNACT1,CYP1A1,CYP2B6,CYP2J2,CYP2U1,CYP3A7,CYP4X1,SULT1A1,SULT4A1,UGT1A7 (includes others) |
| Il-12 signaling and production in macrophages | 1,78E00 | 1,39E-01 | 0,000 | APOA1,APOL1,FGFR1,FGFR4,FRS2,IKBKB,IKBKG,IL18,MAP3K8,MAPK10,MAPK12,MAPK14,MYD88,NOS2,PIK3C2B,PIK3R3,PPARG,PRKCH,RAB7A,RELA |
| T cell receptor signaling | 1,76E00 | 1,48E-01 | 0,000 | CD247,FGFR1,FGFR4,FRS2,IKBKB,IKBKG,LAT,NFATC1,NFATC2,PIK3C2B,PIK3R3,PPP3CA,RELA,RRAS2,TXK,VAV2 |
| Aryl hydrocarbon receptor signaling | 1,74E00 | 1,4E-01 | 0,632 | ALDH1L1,ALDH3A1,ALDH3A2,ALDH3B1,APAF1,CYP1A1,ESR1,ESR2,GSTT2/GSTT2B,HSPB1,NFIB,NRIP1,POLA1,RARB,RARG,RELA,RXRB,SRC,TP73 |
| Role of pkr in interferon induction and antiviral response | 1,73E00 | 1,95E-01 | 0,000 | APAF1,BID,CASP8,IKBKB,IKBKG,MAPK14,NFKBID,RELA |
| Choline biosynthesis iii | 1,73E00 | 3,08E-01 | 0,000 | NAPEPLD,PCYT1B,PHKA1,PLD1 |
| Erk5 signaling | 1,7E00 | 1,67E-01 | 0,302 | FOXO3,MAP3K8,MAPK7,NTRK1,RPS6KA2,RPS6KA5,RPS6KB1,RRAS2,SGK1,SRC,WNK1 |
| Uvb-induced mapk signaling | 1,7E00 | 1,67E-01 | 0,302 | FGFR1,FGFR4,FRS2,MAPK10,MAPK12,MAPK14,PIK3C2B,PIK3R3,PRKCH,RPS6KA5,RPS6KB1 |
| Fc epsilon ri signaling | 1,7E00 | 1,43E-01 | -0,728 | FCER1G,FGFR1,FGFR4,FRS2,INPP5J,LAT,MAP2K7,MAPK10,MAPK12,MAPK14,PIK3C2B,PIK3R3,PRKCH,RRAS2,SYNJ1,SYNJ2,VAV2 |
| Natural killer cell signaling | 1,67E00 | 1,42E-01 | 0,000 | CD244,CD247,FCER1G,FGFR1,FGFR4,FRS2,HCST,INPP5J,LAT,PIK3C2B,PIK3R3,PRKCH,PTPN6,RRAS2,SYNJ1,SYNJ2,VAV2 |
| Inflammasome pathway | 1,67E00 | 2,5E-01 | -0,447 | CASP8,IL18,MYD88,NAIP,NLRP1 |
| Lymphotoxin β receptor signaling | 1,65E00 | 1,64E-01 | 0,000 | APAF1,DIABLO,FGFR1,FGFR4,FRS2,IKBKB,IKBKG,NFKBID,PIK3C2B,PIK3R3,RELA |
| Phospholipases | 1,63E00 | 1,69E-01 | 0,000 | NAPEPLD,PLCB3,PLCB4,PLCD4,PLCE1,PLCH2,PLCL1,PLCL2,PLD1,PNPLA8 |
| Growth hormone signaling | 1,63E00 | 1,53E-01 | 0,000 | FGFR1,FGFR4,FRS2,IGF1,IGFALS,PIK3C2B,PIK3R3,PRKCH,PTPN6,RPS6KA2,RPS6KA5,RPS6KB1,SOCS4 |
| Il-17a signaling in fibroblasts | 1,63E00 | 0,2 | 0,000 | IKBKB,IKBKG,MAPK12,MAPK14,NFKBID,NFKBIZ,RELA |
| Egf signaling | 1,61E00 | 1,62E-01 | 1,508 | CSNK2B,FGFR1,FGFR4,FRS2,MAP2K7,MAPK12,MAPK14,PIK3C2B,PIK3R3,RPS6KB1,SRC |
| Fmlp signaling in neutrophils | 1,6E00 | 1,39E-01 | 0,243 | ACTR2,FGFR1,FGFR4,FRS2,GNAS,IKBKG,NFATC1,NFATC2,NFKBID,PIK3C2B,PIK3R3,PLCB3,PLCB4,PPP3CA,PRKCH,RELA,RRAS2 |
| Angiopoietin signaling | 1,6E00 | 1,56E-01 | -1,633 | FGFR1,FGFR4,FRS2,GRB14,IKBKB,IKBKG,NFKBID,PIK3C2B,PIK3R3,RELA,RRAS2,TIE1 |
| Hereditary breast cancer signaling | 1,59E00 | 1,35E-01 | 0,000 | ARID2,CCNB1,FANCB,FANCM,FGFR1,FGFR4,FRS2,HDAC10,HDAC6,MLH1,MSH6,PALB2,PIK3C2B,PIK3R3,POLR2A,POLR2J2/POLR2J3,RAD50,RRAS2,SLC19A1 |
| Il-15 production | 1,59E00 | 2,14E-01 | 0,000 | LTK,PTK7,RELA,TWF1,TXK,TYK2 |
| Cntf signaling | 1,59E00 | 1,67E-01 | 0,000 | FGFR1,FGFR4,FRS2,PIK3C2B,PIK3R3,RPS6KA2,RPS6KA5,RPS6KB1,RRAS2,TYK2 |
| Adipogenesis pathway | 1,58E00 | 1,36E-01 | 0,000 | ARNTL,ATG5,DGKD,EBF1,EGR2,EZH2,FGF1,FGFR1,FGFR4,FZD3,HDAC10,HDAC6,LPIN1,PPARG,RBBP4,RPS6KB1,SMAD3,ZNF423 |
| Nad salvage pathway ii | 1,58E00 | 2,38E-01 | 0,000 | ACP6,ACPP,NMRK2,NT5C3A,NT5M |
| Phagosome formation | 1,57E00 | 1,38E-01 | 0,000 | FCER1G,FGFR1,FGFR4,FRS2,ITGA4,PIK3C2B,PIK3R3,PLCB3,PLCB4,PLCD4,PLCE1,PLCH2,PLCL1,PLCL2,PRKCH,RND3,TLR1 |
| Hgf signaling | 1,56E00 | 1,4E-01 | -0,775 | ELF3,FGFR1,FGFR4,FRS2,ITGA4,MAP2K7,MAP3K13,MAP3K4,MAP3K8,MAPK10,MAPK12,PIK3C2B,PIK3R3,PRKCH,RAPGEF1,RRAS2 |
| Axonal guidance signaling | 1,55E00 | 1,1E-01 | 0,000 | ABLIM2,ACE,ACTR2,ADAM17,ADAM19,ADAM8,ARHGEF7,EPHA10,EPHA3,EPHB4,EPHB6,FGFR1,FGFR4,FRS2,FZD3,GNAO1,GNAS,IGF1,ITGA4,MMP2,MYSM1,NCK2,NFATC1,NFATC2,NRP2,NTRK1,NTRK2,PIK3C2B,PIK3R3,PLCB3,PLCB4,PLCD4,PLCE1,PLCH2,PLCL1,PLCL2,PPP3CA,PRKCH,RASSF5,RGS3,ROBO3,RRAS2,SEMA3B,SEMA4A,SEMA6C,SLIT1,SRGAP2,SRGAP3,VEGFA |
| Nf-κb activation by viruses | 1,55E00 | 1,49E-01 | -0,832 | FGFR1,FGFR4,FRS2,IKBKB,IKBKG,ITGA4,ITGAL,NFKBID,PIK3C2B,PIK3R3,PRKCH,RELA,RRAS2 |
| Superpathway of melatonin degradation | 1,54E00 | 1,64E-01 | 0,000 | CSGALNACT1,CYP1A1,CYP2B6,CYP2J2,CYP2U1,CYP3A7,CYP4X1,SULT1A1,SULT4A1,UGT1A7 (includes others) |
| Melatonin signaling | 1,53E00 | 1,57E-01 | 2,111 | GNAO1,GNRH1,MAP2K7,PLCB3,PLCB4,PLCD4,PLCE1,PLCH2,PLCL1,PLCL2,PRKCH |
| Nicotine degradation ii | 1,52E00 | 1,7E-01 | 0,000 | CSGALNACT1,CYP1A1,CYP2B6,CYP2J2,CYP2U1,CYP3A7,CYP4X1,FMO5,UGT1A7 (includes others) |
| Acetone degradation i (to methylglyoxal) | 1,52E00 | 2,07E-01 | 0,000 | CYP1A1,CYP2B6,CYP2J2,CYP2U1,CYP3A7,CYP4X1 |
| Estrogen-dependent breast cancer signaling | 1,52E00 | 1,52E-01 | 0,000 | ESR1,FGFR1,FGFR4,FRS2,HSD17B3,IGF1,PIK3C2B,PIK3R3,RELA,RRAS2,SRC,TERT |
| Cyclins and cell cycle regulation | 1,52E00 | 1,52E-01 | 0,333 | BTRC,CCNB1,CCNB3,CDC25A,E2F5,E2F8,HDAC10,HDAC6,PPP2R2B,PPP2R3B,PPP2R5C,PTPA |
| Histamine degradation | 1,5E00 | 2,67E-01 | 0,000 | ALDH1L1,ALDH3A1,ALDH3A2,ALDH3B1 |
| Adenosine nucleotides degradation ii | 1,5E00 | 2,67E-01 | 0,000 | ACPP,ADAL,NT5C3A,NT5M |
| Regulation of eif4 and p70s6k signaling | 1,5E00 | 1,3E-01 | 0,000 | EIF2B4,EIF4G1,EIF4G2,FGFR1,FGFR4,FRS2,ITGA4,MAPK12,MAPK14,PABPC1,PAIP2,PIK3C2B,PIK3R3,PPP2R2B,PPP2R3B,PPP2R5C,PTPA,RPS6KB1,RPS9,RRAS2 |
| G-protein coupled receptor signaling | 1,48E00 | 1,17E-01 | 0,000 | ADORA2A,AVPR1B,FGFR1,FGFR4,FRS2,GNAO1,GNAS,GRK4,GRM5,HTR2A,IKBKB,IKBKG,MAP3K8,MPPE1,NAPEPLD,NFKBID,OPRK1,PDE4A,PDE4B,PDE6G,PDE8A,PIK3C2B,PIK3R3,PLCB3,PLCB4,PTH1R,RAP1GAP,RELA,RGS12,RGS7,RRAS2,SRC |
| Dopamine degradation | 1,46E00 | 0,2 | 0,000 | ALDH1L1,ALDH3A1,ALDH3A2,ALDH3B1,SULT1A1,SULT4A1 |
| D-myo-inositol-5-phosphate metabolism | 1,45E00 | 1,28E-01 | 0,000 | ACP6,CDC25A,ILKAP,PLCB3,PLCB4,PLCD4,PLCE1,PLCH2,PPM1K,PPP1R12A,PPP1R7,PPP3CA,PPP4C,PTPA,PTPN22,PTPN6,STYX,STYXL1,SYNJ1,TPTE |
| Pdgf signaling | 1,45E00 | 1,44E-01 | 0,277 | CSNK2B,FGFR1,FGFR4,FRS2,INPP5J,PIK3C2B,PIK3R3,RRAS2,SPHK1,SRC,SYNJ1,SYNJ2,TYK2 |
| Icos-icosl signaling in t helper cells | 1,44E00 | 1,36E-01 | -0,832 | CD247,FCER1G,FGFR1,FGFR4,FRS2,ICOSLG/LOC102723996,IKBKB,IKBKG,LAT,NFATC1,NFATC2,NFKBID,PIK3C2B,PIK3R3,PPP3CA,RELA |
| Rhogdi signaling | 1,43E00 | 1,25E-01 | -0,894 | ACTR2,ARHGAP12,ARHGAP9,ARHGEF16,ARHGEF18,ARHGEF7,ARHGEF9,CDH24,CDH7,ESR1,ESR2,GNAO1,GNAS,GRIP1,ITGA4,PIP5K1A,PIP5K1C,PIP5KL1,PPP1R12A,PPP1R12B,RND3,SRC |
| Leukocyte extravasation signaling | 1,43E00 | 1,21E-01 | -0,853 | ARHGAP12,ARHGAP9,CLDN14,CLDN2,CTNNA1,CTTN,FGFR1,FGFR4,FRS2,ITGA4,ITGAL,MAP3K4,MAPK10,MAPK12,MAPK14,MMP2,PIK3C2B,PIK3R3,PRKCH,RAP1GAP,RASSF5,SRC,TIMP2,TXK,VAV2 |
| Osteoarthritis pathway | 1,43E00 | 1,21E-01 | -1,877 | CASP4,CASP8,CCN4,COL10A1,CTNNA1,ELF3,FGFR1,FOXO3,FZD3,IL1R1,IL1RAP,IL1RL2,ITGA4,LEF1,NOS2,PPARG,PPARGC1A,PTH1R,RBPJ,RELA,SMAD3,SPHK1,SPP1,TCF7L2,VEGFA |
| Granzyme b signaling | 1,41E00 | 2,5E-01 | -1,000 | APAF1,BID,CASP8,LMNB1 |
| Nicotine degradation iii | 1,41E00 | 1,7E-01 | 0,000 | CSGALNACT1,CYP1A1,CYP2B6,CYP2J2,CYP2U1,CYP3A7,CYP4X1,UGT1A7 (includes others) |
| Paxillin signaling | 1,4E00 | 1,36E-01 | 0,535 | ARHGEF7,FGFR1,FGFR4,FRS2,GIT2,ITGA4,ITGAL,MAPK10,MAPK12,MAPK14,NCK2,PIK3C2B,PIK3R3,RRAS2,SRC |
| Thyroid cancer signaling | 1,39E00 | 1,79E-01 | 0,000 | LEF1,NTRK1,NTRK2,PPARG,RRAS2,RXRB,TCF7L2 |
| Estrogen biosynthesis | 1,39E00 | 1,79E-01 | 0,000 | CYP1A1,CYP2B6,CYP2J2,CYP2U1,CYP3A7,CYP4X1,HSD17B3 |
| P38 mapk signaling | 1,38E00 | 1,33E-01 | -0,832 | DAXX,HSPB1,IL18,IL1R1,IL1RAP,IL1RL2,IL1RN,IRAK1,MAPK12,MAPK14,MAPT,MAX,RPS6KA2,RPS6KA5,RPS6KB1,TAB1 |
| Netrin signaling | 1,37E00 | 1,54E-01 | 0,000 | ABLIM2,CACNA1C,CACNA1D,CACNA2D1,CACNB1,CACNG7,NCK2,NFATC1,NFATC2,PPP3CA |
| Flt3 signaling in hematopoietic progenitor cells | 1,37E00 | 1,45E-01 | 0,000 | FGFR1,FGFR4,FLT3LG,FRS2,MAPK12,MAPK14,PIK3C2B,PIK3R3,RPS6KA2,RPS6KA5,RPS6KB1,RRAS2 |
| P53 signaling | 1,37E00 | 1,35E-01 | -0,832 | APAF1,FGFR1,FGFR4,FRS2,MAPK14,MDM4,PIDD1,PIK3C2B,PIK3R3,PMAIP1,RRM2B,SERPINE2,STAG1,TNFRSF10A,TP73 |
| Wnt/β-catenin signaling | 1,37E00 | 1,24E-01 | -0,243 | APC2,BTRC,CSNK1G1,CSNK2B,FZD3,GNAO1,KREMEN1,LEF1,MARK2,NR5A2,PPP2R2B,PPP2R3B,PPP2R5C,PTPA,RARB,RARG,SOX5,SRC,TAB1,TCF7L2,TLE3 |
| Oxidative ethanol degradation iii | 1,32E00 | 2,35E-01 | 0,000 | ALDH1L1,ALDH3A1,ALDH3A2,ALDH3B1 |
| Il-17 signaling | 1,31E00 | 1,41E-01 | 0,000 | FGFR1,FGFR4,FRS2,IL19,MAPK10,MAPK12,MAPK14,NOS2,PIK3C2B,PIK3R3,RELA,RRAS2 |
| Leptin signaling in obesity | 1,31E00 | 1,41E-01 | 0,447 | FGFR1,FGFR4,FRS2,PIK3C2B,PIK3R3,PLCB3,PLCB4,PLCD4,PLCE1,PLCH2,PLCL1,PLCL2 |
| 3-phosphoinositide degradation | 1,3E00 | 1,25E-01 | 0,000 | ACP6,CDC25A,ILKAP,INPP4B,INPP5J,MTMR3,PPM1K,PPP1R12A,PPP1R7,PPP3CA,PPP4C,PTPA,PTPN22,PTPN6,STYX,STYXL1,SYNJ1,SYNJ2,TPTE |

**Supplemental Table S10 : List of most significant upstream regulators in Insulin (INS) treatment (p-value of overlap ≤ 0,05).**

| INSULIN TREATMENT | | | | | | |
| --- | --- | --- | --- | --- | --- | --- |
| Upstream Regulator | Expr Log Ratio | Molecule Type | Predicted Activation State | Activation z-score | p-value of overlap | Target molecules in dataset |
| ESR2 |  | ligand-dependent nuclear receptor |  | -0,873 | 1,23E-04 | BBC3,BID,EGFR,FOXM1,GRPR,MME,NDRG2,PGR,SCARB1,TGFA,TNFSF13B,VEGFA |
| HNRNPA2B1 |  | other |  |  | 1,68E-04 | ATRX,CACNB2,CADM1,FN1,GPRC5B,HMGB3,HYAL1,LEPR,LETM2,MAST4,MEIS2,NR3C2,NRCAM,NTS,OAS1,PEG10,PITPNC1,PKIB,PLCB4,PPARGC1A,PRSS3,SCN9A,SFI1,SULT1A1,WWOX |
| TP53 |  | transcription regulator |  | 1,516 | 4,31E-04 | AKAP12,AR,BAP1,BBC3,BID,BUB1,CASP8,CCNL1,CDC25A,CDH10,CDK2,CDKN3,CHEK2,CSF2,CYP26B1,DAPK1,DICER1,DLX1,EDA2R,EGFR,F5,FAS,FOXP3,GADD45A,GAL3ST4,GART,GOLGA4,HMGN2,HSPA8,KIF23,KRT15,LIPF,MAP3K8,MAP4,MAPK12,MDM4,NECTIN3,NEK2,NOS2,NOX4,OAS1,P2RX4,PACSIN1,PALLD,PCBP4,PEG10,PFKFB3,PFKM,PIDD1,PMAIP1,POLE2,POU5F1,PPP3CA,PRKG1,PROM1,PTK2,RPS6KB1,RRM2B,SCRIB,SELP,SESN1,SIRT1,SIRT6,SORBS1,SRC,SRGAP3,STMN1,SULF2,TAP1,TCF7L2,TGFA,TMEM97,TNFRSF10A,TNFRSF1B,TP73,TPD52L1,VEGFA,ZAP70,ZEB1 |
| ESR1 |  | ligand-dependent nuclear receptor |  | -1,799 | 5,41E-04 | ADAM17,BAZ2A,BCAS3,BCL2L2,C1QTNF6,CCNE1,CDKN3,CLN8,CSF2,EGFR,FAS,FOXM1,GPER1,GREB1,HELLS,KIF23,LEPR,MALL,MAPK12,MME,MND1,NRCAM,NRF1,OTUB2,PDZK1,PGR,PIMREG,PKIB,PLAC1,POLA1,RGS3,RNF38,SCARB1,SEC31A,SESN1,SGK1,SIRT1,SP110,STX3,TGFA,TMEM97,TNFRSF10A,TPM1,TSC22D3,UMODL1,VEGFA,WISP2 |
| FBXW7 |  | transcription regulator |  | 0,447 | 7,31E-04 | ADAM17,ADAM22,DDX11,HSPA8,ITGB3BP,PPARGC1A,STIP1 |
| IL2 |  | cytokine |  | 1,054 | 7,76E-04 | ATM,CCNE1,CDC25A,CSF2,CSF2RA,CSRNP1,DAPK1,DLST,FAS,FOXP3,GART,IL24,ING4,LRMP,LRRC32,NOP2,PDE4A,PIK3R3,POLE2,PPP2R2B,PRDM1,PTPRC,RGPD4 (includes others),SESN1,STIP1,STK17B,TNFRSF10A,TNFRSF11A,TNFRSF18,TNFRSF1B,TNFSF10,VEGFA |
| estrogen receptor |  | group |  | -0,908 | 1,07E-03 | ABCG1,CD68,CDH10,CLN8,CNKSR1,COL4A6,DICER1,EGFR,EGLN2,FGF1,FGFR3,FN1,GJB3,GREB1,ICAM2,ICAM3,KRT15,LY6E,MAPK12,MAPT,NRIP1,PCDH19,PGR,SMN1/SMN2,TGFA,TSC22D3,VEGFA,WNT3,WNT5B,ZEB1 |
| TCF4 |  | transcription regulator |  |  | 2,01E-03 | CDH17,FGF1,NCOR2,NOS2,PACSIN1,SGK1,TCF7L2,WISP2,ZEB1 |
| TIMP3 |  | other |  |  | 2,06E-03 | APP,FAS,MET |
| RET |  | kinase |  |  | 2,06E-03 | BBC3,MET,PMAIP1 |
| AURK |  | group |  | 0,905 | 2,41E-03 | BBC3,CDH10,CYP26B1,EDA2R,GAL3ST4,GOLGA4,SCRIB,SESN1,SRGAP3,SULF2,TNFRSF10A |
| ANLN |  | other |  | 0,905 | 2,41E-03 | BBC3,CDH10,CYP26B1,EDA2R,GAL3ST4,GOLGA4,SCRIB,SESN1,SRGAP3,SULF2,TNFRSF10A |
| NR3C1 | -5,169 | ligand-dependent nuclear receptor |  | 0,370 | 2,47E-03 | AIFM3,APOL3,APTX,BCL2L2,BMF,BRWD1,C1QTNF1,CARD14,CIDEA,COQ8A,CSF2,CSRNP1,CTNNBL1,DAPK2,DAXX,DIABLO,ELMOD1,FN1,GADD45A,IL18,IL1RAP,IL24,ING1,IP6K3,MAPK7,MDM4,NAIP,NOS2,NR3C1,PAK6,PIK3CD,PIK3R1,PIK3R3,PLAGL1,POU5F1,PPP3CA,PRKAR1B,RELA,RNF38,RTN4,SCARB1,SGK1,SLC19A2,STAT6,STK17B,TNFRSF10A,TNFRSF1B,TRIM45,TSC22D3 |
| EHMT2 |  | transcription regulator |  | 0,577 | 2,70E-03 | C8orf44-SGK3/SGK3,GREB1,PGR,PKIB,PMAIP1 |
| Histone h3 |  | group |  |  | 4,30E-03 | CCNE1,COL4A6,FMR1,FOXM1,FOXP4,HBE1,HOXA11,HOXB6,ICAM3,MEIS1,MET,MX1,NDUFB1,NODAL,NRF1,PALLD,PCDHA8,POU5F1,PPP2R2B,PSG5,PTGES,RCAN1,RPS9,RUNX1T1,SBF1,SEC31A,SPHK1,TNFSF10,WASF1,WNT8B,ZEB1,ZNF423 |
| MCM7 |  | enzyme |  |  | 4,84E-03 | CDK2,EGFR,MET |
| PANDAR |  | other |  |  | 4,84E-03 | BBC3,PIDD1,PMAIP1 |
| NFIL3 |  | transcription regulator |  |  | 4,84E-03 | FAS,GADD45A,TNFSF10 |
| OTUB1 |  | enzyme |  |  | 4,84E-03 | FOXM1,GREB1,PGR |
| HOTAIR |  | other |  | -0,468 | 5,49E-03 | ABL2,LAMB3,MX1,OAS1,ZEB1 |
| mir-17 |  | microrna |  | -1,049 | 5,49E-03 | APP,AR,ATM,FAS,POU5F1 |
| RABL6 |  | other |  | -0,577 | 6,01E-03 | BUB1,CDC25A,CDK2,CHEK2,DAPK1,FERMT2,KIF23,NEK2,PMAIP1,POLA1,POLE2,TMEM97 |
| PDGFB |  | growth factor |  |  | 6,71E-03 | BBC3,VEGFA |
| miR-1298-5p (and other miRNAs w/seed UCAUUCG) |  | mature microrna |  |  | 6,71E-03 | LAMB3,PTK2 |
| MGAT5 |  | enzyme |  |  | 6,71E-03 | EGFR,PROM1 |
| SNRNP70 |  | other |  |  | 6,71E-03 | APP,MDM4 |
| KMT2A |  | transcription regulator |  | 1,000 | 7,45E-03 | HOXA11,MEIS1,PGR,PROM1,SCARB1 |
| CSF1 |  | cytokine | Inhibited | -2,433 | 7,67E-03 | FAS,FN1,HSP90B1,OSCAR,STAT1,TNFRSF1B,VEGFA |
| TNFRSF11A | -8,564 | transmembrane receptor |  |  | 9,09E-03 | FN1,MME,POU5F1 |
| TNFSF11 |  | cytokine |  |  | 9,27E-03 | CSF1R,ITGAL,STAT1,TNFRSF11A |
| POU5F1 | 6,851 | transcription regulator |  | 1,445 | 1,04E-02 | BCL2L2,BID,BNIP1,CIDEA,CIDEB,DAPK1,DLX1,FAS,GADD45A,MEF2C,MEIS1,NAIP,PAX6,PMAIP1,POU5F1,TNFRSF10A,TNFSF10,TP73,ZEB1 |
| APOE |  | transporter |  |  | 1,04E-02 | ADGRG1,ATXN3,CLN8,DIP2A,KCNAB1,NELL2,PIDD1,PPFIBP2,PRPSAP2,SEMA6B,SIRT1,SORBS1,TNIP1 |
| PTEN |  | phosphatase |  | 0,218 | 1,12E-02 | AR,BBC3,CDC25A,EGFR,ING4,PXN,SRC |
| MYOCD |  | transcription regulator |  | 0,544 | 1,14E-02 | ACTC1,CACNA1C,HSPB7,MEF2C,NFATC2,PHKA1,PTPRB,TNNI1,TNNT2,TPM1 |
| TLR3 |  | transmembrane receptor |  |  | 1,20E-02 | CSF2,LMNB1,MAP3K8,MX1,NOS2,OAS1,PFDN6,PIK3CD,PIK3R1,PIK3R3,PMAIP1,STAT1,TNFSF10,TNFSF13B |
| mir-30 |  | microrna |  | -0,577 | 1,30E-02 | AR,BBC3,GADD45A,PRDM1 |
| IDH1 |  | enzyme |  |  | 1,30E-02 | CLDN7,FN1,MAPK10,ZEB1 |
| E2F6 |  | transcription regulator |  |  | 1,48E-02 | CCNE1,CDC25A,CHEK2,DDX11,GRIK1,LIG1,NEK11,RBBP8,ZNF43 |
| E2F7 |  | transcription regulator |  |  | 1,49E-02 | ECT2,KIF23,RACGAP1 |
| TRPV2 |  | ion channel |  |  | 1,49E-02 | CASP8,CCNE1,FAS |
| PURA |  | transcription regulator |  |  | 1,49E-02 | AR,CASP8,ITGAL |
| miR-491-5p (and other miRNAs w/seed GUGGGGA) |  | mature microrna |  |  | 1,49E-02 | AR,PTK2,PXN |
| MAPK3 |  | kinase |  |  | 1,49E-02 | SIRT6,SLC4A7,TNFRSF10A |
| ID2 |  | transcription regulator |  |  | 1,49E-02 | NTRK2,POU2F2,SEMA3F |
| KAT5 |  | transcription regulator |  | -1,212 | 1,50E-02 | BBC3,CACNB2,EPG5,GRIA4,MAPT,OPRK1,PLCH1,POLI,SCN2A,SNPH,TDG,VPS53 |
| APP | 7,612 | other |  | -0,076 | 1,61E-02 | APP,MME,NOS2,SLC11A2,TNFSF10 |
| NR3C2 | 6,971 | ligand-dependent nuclear receptor |  |  | 1,61E-02 | CABIN1,NDRG2,SGK1,TSC22D3,XIRP1 |
| GATA4 |  | transcription regulator |  | 0,688 | 1,65E-02 | ACTC1,CLDN2,HSPB7,NFATC2,NR5A2,NUSAP1,PHKA1,PTPRB,TNNT2,TPM1 |
| MMP14 |  | peptidase |  | 0,152 | 1,76E-02 | CSF2,DAPK1,NR3C1,PIK3CD |
| Beta Secretase |  | group |  |  | 1,90E-02 | APP,MME |
| SMYD2 |  | enzyme |  |  | 1,90E-02 | GREB1,PGR |
| NAA30 | 5,382 | enzyme |  |  | 1,90E-02 | FAS,PMAIP1 |
| SF3B1 |  | other |  |  | 1,90E-02 | MDM4,PUF60 |
| miR-615-3p (miRNAs w/seed CCGAGCC) |  | mature microrna |  |  | 1,90E-02 | AR,PPARG |
| DNAJA3 |  | other |  |  | 1,90E-02 | EGFR,VEGFA |
| SRSF5 |  | other |  |  | 1,90E-02 | NR3C1,VEGFA |
| Lh |  | complex |  | 0,303 | 2,03E-02 | ACPP,ACTR2,ADGRG1,AKAP12,AR,ATP2B1,DAPK1,DUSP14,EGFR,GPRC5B,HSD3B1,ITPR1,PIK3CD,PLCL1,PMAIP1,PPFIA4,RAB27A,REXO5,RGS7,SGK1,STAT1,STIP1,STK24,TPM1,TRIB1,VEGFA |
| SMAD4 |  | transcription regulator |  | -0,351 | 2,06E-02 | BBC3,CCNE1,CELF2,CYB561,DAXX,GADD45A,IL17B,MAPK7,PAX6,PGRMC2,PTPRC,SGK1,TNFRSF10A,VEGFA |
| ERBB2 |  | kinase |  | -0,181 | 2,09E-02 | AR,BUB1,CADM1,CCNE1,CDC16,CDC25A,CDCA4,CDKN3,CGB3 (includes others),CSF1R,E2F8,EGFR,FN1,GART,LIG1,LIG3,MCM8,NEK2,ORC1,PAICS,PIK3CD,PMEPA1,POLA1,POLE2,POLI,POLR2J2/POLR2J3,POLR3H,PORCN,PPARG,PRSS3,SCARB1,TPM1,VEGFA,WNT5B |
| CDX2 |  | transcription regulator |  |  | 2,15E-02 | BORCS5,CDH17,CLDN2,FGF1,POU5F1,PRDM1,SOX5,TCF7L2,UGT1A7 (includes others) |
| AKT1 |  | kinase |  | 0,342 | 2,15E-02 | CCNE1,CIDEA,DIAPH2,FOSB,NOX4,SIRT6,SLC4A7 |
| ERG |  | transcription regulator |  | 0,745 | 2,15E-02 | ADGRG1,APOC1,AR,DIAPH2,DIP2A,DYNC1I1,EGFL7,IKBKG,MET,NPHP1,NRCAM,PTPN22,PXN,RAPGEF5,RAPH1,RCAN2,RGS3,TSC22D3,ZEB1 |
| E2F8 | 5,460 | transcription regulator |  |  | 2,25E-02 | ECT2,KIF23,RACGAP1 |
| mir-185 |  | microrna |  |  | 2,25E-02 | AR,NTRK2,STIM1 |
| USP18 |  | peptidase |  |  | 2,25E-02 | MX1,OAS1,TNFSF10 |
| EGFR | 8,424 | kinase |  | -0,450 | 2,40E-02 | AR,CLDN2,CSF2,EGFR,FOXP3,HSP90B1,KRT17,MET,NOS2,POSTN,PTGES,RELA,SPHK1,VEGFA,ZEB1 |
| PRDM5 |  | transcription regulator |  |  | 2,45E-02 | ARID3B,CACNA1C,EED,ISLR,ISLR2,LRRC37A3 (includes others),PAX5,PAX6,POU5F1,PPP1R12B,PSG1,RXRB,TINAGL1 |
| RNF31 |  | enzyme |  | 0,447 | 2,47E-02 | C8orf44-SGK3/SGK3,GREB1,NR5A2,PDZK1,SCARB1 |
| TCF7L2 | -7,959 | transcription regulator |  | 0,743 | 2,49E-02 | ADGRG1,ASPH,CLDN2,EGFR,STK17B,VEGFA,WISP2 |
| TBX5 |  | transcription regulator |  | 0,349 | 2,49E-02 | ACTC1,HSPB7,NFATC2,PHKA1,PTPRB,TNNT2,TPM1 |
| HAND2 |  | transcription regulator |  | 0,345 | 2,49E-02 | ACTC1,HSPB7,NFATC2,PHKA1,PTPRB,TNNT2,TPM1 |
| PELP1 | -2,482 | other |  |  | 2,49E-02 | BCAS3,KCNK2,NCOR2,PPARGC1A,SRC,TCF7L2,ZNF804A |
| TNF |  | cytokine |  | 0,894 | 2,62E-02 | ADAM8,ADGRG6,ALOX15B,ATP2B1,BBC3,BID,CSF2,EGFR,FAS,FN1,FOXP3,GADD45A,GNB4,GPD2,IL1R1,IL37,IRF5,ITGA4,ITGAL,KRT15,LAMA3,LAMB3,MET,NOS2,NR3C1,NR5A2,NUP98,OAS1,PDPN,PIK3CD,PMAIP1,PPARG,PPARGC1A,PRDM1,PTGES,PTPRC,RELA,RGS20,SCARB1,SELP,STAT1,TAP1,TGFA,TH,TNFRSF1B,TNFSF10,TNFSF13B,TSC22D3,VEGFA,WISP1 |
| SRF |  | transcription regulator |  |  | 2,67E-02 | AKAP12,EGR2,EPG5,FGF1,FHL2,FILIP1L,FOSB,LRP1,MEIS1,NLRP1,ODF2L,RAI2,ZNF701 |
| SUZ12 |  | enzyme |  |  | 2,67E-02 | ABL2,CALU,CDC25A,HOXB6,KIF2C,KRT17,LAMB3,MGAT4B,PGR,PLCB4,PPP2R2B,TNFRSF1B,VAMP7 |
| let-7 |  | microrna |  | -1,571 | 2,90E-02 | APC2,AR,BCAT1,CDC25A,DDX56,DICER1,FN1,PAICS,PPP1R12B,RELA,STARD13,TRIB1,ZC3H3 |
| miR-124-3p (and other miRNAs w/seed AAGGCAC) |  | mature microrna |  | 0,000 | 2,95E-02 | CD164,NR3C2,SUCLG2,SURF4 |
| FOXP1 |  | other |  | -0,152 | 2,95E-02 | CSF1R,NR5A2,POU5F1,PRDM1 |
| EIF4G1 |  | translation regulator |  | 1,342 | 2,98E-02 | ATM,ATRIP,ATRX,GADD45A,RAD50 |
| mir-1 |  | microrna |  | 0,200 | 2,98E-02 | ATP6V0A1,CSF2,MET,TWF1,VEGFA |
| TENM1 |  | transmembrane receptor |  |  | 3,17E-02 | SCARB1,SEMA6A,SOX5 |
| PTGER2 |  | g-protein coupled receptor |  |  | 3,17E-02 | PGR,PXN,VEGFA |
| TOPBP1 |  | other |  |  | 3,17E-02 | BBC3,COQ8A,GADD45A |
| IL17D |  | cytokine |  |  | 3,17E-02 | IFNLR1,MX1,OAS1 |
| FSH |  | complex |  | 0,447 | 3,38E-02 | ACPP,ACTR2,ADGRG1,AKAP12,AKAP7,ALDH3A2,ATP2B1,DAPK1,DUSP14,FILIP1L,GPRC5B,HSD3B1,ING1,ITPR1,LRRC32,PIK3CD,PLCL1,PMAIP1,PPFIA4,RAB27A,REXO5,RGS7,SCARB1,SGK1,STAT1,STIP1,STK24,TPM1,TRIB1,VEGFA,ZEB1 |
| MAPK1 |  | kinase |  | -0,351 | 3,44E-02 | ADAM17,APBB3,AR,BICRAL,FN1,HOXA11,KRT17,OAS1,OLFML2A,PHF11,PLD1,PRKG1,QKI,SLC4A7,SP110,STAT1,TAP1,TNFSF10,TRIM14,TRIM25,TRPC1,VDR |
| TSPYL5 |  | other |  |  | 3,56E-02 | CAST,FUT8,GLDN,NR3C1,NR3C2 |
| NFYA |  | transcription regulator |  |  | 3,56E-02 | BBC3,GADD45A,GUCY1B3,PIDD1,PMAIP1 |
| ZNF282 |  | transcription regulator |  |  | 3,60E-02 | GREB1,PGR |
| BCAS2 |  | other |  |  | 3,60E-02 | BBC3,PMAIP1 |
| PHF8 |  | enzyme |  |  | 3,60E-02 | CCNE1,CDC25A |
| HCAR1 |  | g-protein coupled receptor |  |  | 3,60E-02 | BSG,PPARGC1A |
| GIT1 |  | kinase |  |  | 3,60E-02 | PTK2,PXN |
| miR-515-5p (and other miRNAs w/seed UCUCCAA) |  | mature microrna |  |  | 3,60E-02 | PIK3C2B,SPHK1 |
| mir-322 |  | microrna |  |  | 3,60E-02 | CCNE1,CDC25A |
| IRS2 |  | enzyme |  |  | 3,60E-02 | MX1,VEGFA |
| ISG15 |  | other |  |  | 3,60E-02 | MX1,OAS1 |
| APEX1 |  | enzyme |  |  | 3,60E-02 | SIRT1,VEGFA |
| DDB1 |  | other |  |  | 3,60E-02 | BBC3,STAT1 |
| ACTA2 |  | other |  |  | 3,60E-02 | MET,PTK2 |
| CCR2 |  | g-protein coupled receptor |  |  | 3,60E-02 | APP,ITGAL |
| DEFB103A/DEFB103B |  | other |  |  | 3,60E-02 | BID,IL18 |
| YAP1 |  | transcription regulator | Activated | 2,438 | 3,71E-02 | CASP8,DICER1,EGFR,FOXM1,NEK2,PMAIP1,TP73 |
| CNOT7 |  | transcription regulator |  |  | 3,74E-02 | HERC6,OAS1,PPM1K,SP110,STAT1,TAP1 |
| PPARG | 6,945 | ligand-dependent nuclear receptor |  | -1,451 | 3,79E-02 | ABCG1,ALOX15B,APH1B,APP,FN1,PPARG,PTGES,SCARB1,TNFSF10,VEGFA |
| P38 MAPK |  | group |  | -0,505 | 4,01E-02 | ADAM17,BBC3,CCNE1,EGR2,FAS,FN1,LAMA3,MAP4,PPARGC1A,RCAN1,STAT1,STIM1,TGFA,TJP2,TNFSF10,TNIP1,TRIB1,VDR,VEGFA |
| AR | 7,678 | ligand-dependent nuclear receptor |  | 0,586 | 4,02E-02 | ABCG1,ALOX15B,AR,ATP1A3,BUB1,CALU,CAST,CLGN,DHCR24,EGFR,IL1R1,KIF2C,MEF2C,MME,MYOM1,NEK2,NUSAP1,PGR,PMEPA1,SLC43A1,STAT1,TPD52L1,VEGFA |
| ETS1 |  | transcription regulator |  | -0,873 | 4,13E-02 | CCNE1,CDK2,CSF2,FN1,MET,PEG10,PHF12,TBXAS1,TGFA,TRPC1,ZEB1 |
| KMT2D |  | transcription regulator |  | 1,172 | 4,20E-02 | ADGRG1,LAMB3,PPP2R2B,SCARB1,TNNT2 |
| mir-15 |  | microrna |  | 0,285 | 4,20E-02 | AR,BCL2L2,EGFR,MX1,OAS1 |
| NMNAT1 |  | enzyme |  |  | 4,26E-02 | NAT1,NELL2,PEG10 |
| SRSF1 |  | other |  |  | 4,26E-02 | FN1,SMN1/SMN2,VEGFA |
| BCL6B |  | transcription regulator |  |  | 4,26E-02 | ATM,CASP8,VEGFA |
| NR2F2 |  | ligand-dependent nuclear receptor |  |  | 4,26E-02 | HBE1,POU5F1,PSG1 |
| NCOA3 |  | transcription regulator |  | -0,387 | 4,30E-02 | CCNE1,CDC25A,CDK2,OTUB2,PGR,PPARG |
| USP7 |  | peptidase |  | 1,000 | 4,51E-02 | BMF,FAM111B,FOXP3,TRPC4 |
| SLC29A1 |  | transporter |  | 1,000 | 4,51E-02 | FAS,GADD45A,RRM2B,SESN1 |
| IFN Beta |  | group |  |  | 4,90E-02 | FN1,IL24,MX1,SLC16A6,SPRY1,STAT1 |
| FANCC |  | other |  |  | 4,91E-02 | CASK,HOMER1,IL1R1,MX1,NR3C1,OAS1,PTPRC,SYPL1 |

**Supplemental Table S11: List of most significant upstream regulators in High fat (HF) treatment (p-value of overlap ≤ 0,05).**

| HIGH FAT |  |  |  |  |  |  |  |
| --- | --- | --- | --- | --- | --- | --- | --- |
| Upstream Regulator | Expr Log Ratio | Molecule Type | Predicted Activation State | Activation z-score | p-value of overlap | Target molecules in dataset |  |
| ERG | -7,011 | transcription regulator |  | 1,104 | 3,00E-06 | ADGRG1,APOC1,ARHGAP24,AXIN2,BCR,CD59,DIAPH2,DIAPH3,DIP2A,ERG,FYN,GFRA3,HDAC6,HMMR,LEF1,MET,MYO1D,NPHP1,NRCAM,PTPN22,PXN,RAPGEF1,RAPGEF5,RASSF1,RCAN2,RGS3,SLIT2,TSC22D3,WIPF1,ZEB1 |  |
| TCF4 | -7,362 | transcription regulator |  | 1,086 | 1,52E-04 | AXIN2,CBFA2T3,CDH17,GLI1,LEF1,NCOR2,PSD3,RUNX2,TCF7L2,WISP2,ZEB1 |  |
| PRDM5 |  | transcription regulator |  |  | 3,92E-04 | ARID3B,CACNA1C,CDH4,EBF1,EVL,IL6R,ISLR,ISLR2,MYB,MYD88,MYOM2,NAV2,PML,POU5F1,RARA,RUNX1,RUNX2,TINAGL1 |  |
| mir-33 |  | microrna |  | -1,118 | 1,42E-03 | ACACA,CPT1A,FRS2,HMGA2,PPARG |  |
| FSH |  | complex |  | 0,277 | 1,78E-03 | ADGRG1,AKAP12,AKAP7,ATP2B1,BDNF,CAMK2G,CARD10,CDKN1C,CREM,CYFIP2,DAPK1,DUSP14,EFNA1,FILIP1L,FSHR,GK,GPRC5B,ING1,ITPR1,KIDINS220,LRRC32,MAMLD1,MAP3K7,MAPK14,MBD2,MMP2,MT3,PPFIA4,RAB27A,RAB5C,SCARB1,SH3BP4,SNAP23,STIP1,TGFBR3,TPM1,TRIB1,ZEB1 |  |
| estrogen receptor |  | group |  | -0,126 | 2,40E-03 | ABCG2,AGO2,AKT3,CALD1,CAPG,CD59,CDH4,CLN8,CNKSR1,DICER1,EGLN2,ERBB3,ERBB4,GJB3,ICAM2,LY6E,MAP1B,MAPK12,MAPT,NRIP1,OCLN,PCDH18,PCDH19,PGR,SMN1/SMN2,TGFB3,TGFBR3,TSC22D3,WNT5B,ZEB1 |  |
| GIT1 |  | kinase |  |  | 2,41E-03 | MMP2,PTK2,PXN |  |
| TIMP3 |  | other |  |  | 2,41E-03 | APP,FAS,MET |  |
| MAPK14 | -5,517 | kinase |  | 1,977 | 2,51E-03 | AXIN2,BACE1,BAX,DICER1,DNMT3A,LEF1,LRP6,VDR |  |
| PPARGC1A |  | transcription regulator |  | -0,103 | 3,53E-03 | ACADM,BAX,CD36,COL2A1,CPT1B,CYP1A1,CYP2A6 (includes others),GK,RUNX2 |  |
| let-7 |  | microrna |  | -0,429 | 3,72E-03 | AGO2,AURKB,BDNF,CDC25A,DDX56,DICER1,E2F5,EIF4A1,GAB2,HMGA2,LEF1,MYD88,RDH10,SLC25A13,TARBP2,TRIB1 |  |
| APP | 2,264 | other |  | -0,736 | 4,17E-03 | ACHE,APP,BAX,MME,SLC11A2,TNFSF10 |  |
| TFRC |  | transporter |  | 0,000 | 4,27E-03 | ADGRG1,FAM20C,GADD45A,LRRC25,PPARG,SLC26A11,SLC2A14,SULF2,TNFSF10 |  |
| TENM1 |  | transmembrane receptor | Activated | 2,000 | 4,90E-03 | CHL1,ERBB3,SCARB1,SOX5 |  |
| UPF1 |  | enzyme |  | 0,555 | 4,93E-03 | ERG,HMGA2,SMG6,SMG7,UPF2 |  |
| NFIL3 |  | transcription regulator |  |  | 5,63E-03 | FAS,GADD45A,TNFSF10 |  |
| NAT8 |  | transcription regulator |  |  | 7,46E-03 | APP,BACE1 |  |
| NAT8B |  | enzyme |  |  | 7,46E-03 | APP,BACE1 |  |
| SNRNP70 |  | other |  |  | 7,46E-03 | APP,MDM4 |  |
| HNRNPA2B1 |  | other |  |  | 8,93E-03 | ADGRG2,AKT3,ATRX,CACNB2,CADM1,DDR1,ERBB3,EYA4,GPRC5B,HMGB3,HYAL1,LPAR1,NPR3,NRCAM,PDE1A,PEG10,PITPNC1,PLCB4,PRSS3,SERPINA1,SFI1 |  |
| JAK2 |  | kinase |  |  | 9,16E-03 | BAX,CD36,CDC25A,LMO2,MPL,RARA |  |
| AMER1 |  | other |  |  | 1,05E-02 | APAF1,AXIN2,BAX |  |
| WNT3A |  | cytokine |  |  | 1,05E-02 | AXIN2,LEF1,LRP6 |  |
| EIF2AK3 |  | kinase |  | 1,067 | 1,12E-02 | ANG,ATG5,DDIT3,PRDM1 |  |
| FABP2 |  | transporter |  | 0,762 | 1,12E-02 | CPT1A,NPC1L1,NR1H3,PPARG |  |
| SERPINF1 |  | other |  | 0,152 | 1,12E-02 | AXIN2,LRP6,PPARG,TNFSF10 |  |
| RUNX3 |  | transcription regulator |  |  | 1,12E-02 | AKT1,BAX,ITGAL,RUNX1 |  |
| SOX11 |  | transcription regulator |  | 0,084 | 1,61E-02 | CNOT6,EBF1,FAS,FCMR,HMGB3,ITGA4,LEF1,MYO1B,PRDM1,SEPT2,SPIB,TFDP2 |  |
| NR3C1 |  | ligand-dependent nuclear receptor |  | -0,232 | 1,61E-02 | AIFM3,AKT1,ANGPTL4,APOL3,APTX,BABAM2,BARD1,BMF,BRWD1,C1QTNF7,CARD10,CARD14,CDKN1C,CORO1C,CPEB4,CSRNP1,DEAF1,ELMOD3,GADD45A,IL15RA,IL1RAP,ING1,KAT5,LGALS8,MAP3K14,MAP3K7,MAPK1,MAPK7,MAX,MDM4,MYD88,NFATC1,NGFR,PIK3R3,POU5F1,PPP3CA,RBMS3,RTN4,SCARB1,SCNN1A,SON,STAT6,STK17B,TESMIN,TNFRSF1B,TNFRSF25,TSC22D3 |  |
| SLC4A1 |  | transporter |  |  | 1,73E-02 | AQP1,ICAM4,RHCE/RHD |  |
| PURA |  | transcription regulator |  |  | 1,73E-02 | CASP8,ITGAL,ITGAX |  |
| miR-491-5p (and other miRNAs w/seed GUGGGGA) |  | mature microrna |  |  | 1,73E-02 | MMP2,PTK2,PXN |  |
| LMO2 | 6,795 | transcription regulator |  |  | 1,73E-02 | ADGRG1,ERG,NKX3-1 |  |
| RHOC |  | enzyme |  |  | 1,73E-02 | CDKN1C,DDIT3,NAT8L |  |
| POU5F1 | 5,820 | transcription regulator |  | 0,317 | 1,74E-02 | AKT1,APAF1,BAX,BID,BNIP1,CASP1,CIDEB,DAPK1,DLX1,DLX4,EOMES,FAS,GADD45A,MEIS1,MMP2,POU5F1,TNFRSF25,TNFSF10,ZEB1 |  |
| IL2 |  | cytokine |  | 1,234 | 1,99E-02 | CARD10,CASP1,CD59,CDC25A,CDKN1C,CSF2RA,CSRNP1,DAPK1,DLST,EPHA4,FAS,IL18BP,ING4,LRMP,LRRC32,NOP2,PDE4A,PIK3R3,PRDM1,PTPRC,RGPD4 (includes others),SPRED2,STIP1,STK17B,TFDP2,TNFRSF18,TNFRSF1B,TNFSF10 |  |
| IRF2 |  | transcription regulator |  |  | 1,99E-02 | MYH9,PSMB8,TAP2,TAPBP,TNFSF10 |  |
| BMP4 |  | growth factor |  | 0,180 | 2,10E-02 | ABCG2,ADGRG1,CDK2,DLX4,EFNA1,HEY1,ITGA4,MFAP5,POU5F1,PPARG,PRKCH,PRR5,TFAP2A |  |
| miR-30a-3p (and other miRNAs w/seed UUUCAGU) |  | mature microrna |  | -1,000 | 2,10E-02 | CC2D1B,KIF1B,LIMA1,WDR44 |  |
| Beta Secretase |  | group |  |  | 2,11E-02 | APP,MME |  |
| GRHL2 |  | transcription regulator |  |  | 2,11E-02 | ERBB3,ZEB1 |  |
| miR-892b (miRNAs w/seed ACUGGCU) |  | mature microrna |  |  | 2,11E-02 | MAP3K7,TAB3 |  |
| miR-615-3p (miRNAs w/seed CCGAGCC) |  | mature microrna |  |  | 2,11E-02 | LCOR,PPARG |  |
| PSEN1 |  | peptidase |  |  | 2,11E-02 | APP,BACE1 |  |
| USP8 |  | peptidase |  |  | 2,11E-02 | APP,BACE1 |  |
| Lh |  | complex |  | 0,594 | 2,16E-02 | ADGRG1,AKAP12,ATP2B1,CAMK2G,CARD10,CDKN1C,CYFIP2,DAPK1,DUSP14,GK,GPRC5B,ITPR1,KIDINS220,MAP3K7,MAPK14,MBD2,MMP2,MT3,PPFIA4,PRKAR2A,RAB27A,RAB5C,SH3BP4,SNAP23,STIP1,TPM1,TRIB1 |  |
| FOXM1 |  | transcription regulator |  | 0,470 | 2,19E-02 | ATF2,AURKB,AXIN2,CDC25A,CDK2,CDKN3,MAPK8,MET,MMP2,NEK2,SFTPD,STMN1 |  |
| GATA4 |  | transcription regulator |  | -0,635 | 2,30E-02 | ACTC1,CLDN2,EPHX1,HSPB7,MYH9,NFATC2,NUSAP1,PTPRB,RARB,TPM1 |  |
| NANOG |  | transcription regulator |  | -0,194 | 2,39E-02 | DLX1,DLX4,EOMES,FOSB,GMFG,MAP1B,MEIS1,NPIPA7 (includes others),NRIP1,PCDHA2,PRRC2C,RAPGEF5,TBC1D3 (includes others),ZFP42 |  |
| RUNX2 | -3,561 | transcription regulator |  | 0,600 | 2,54E-02 | COL10A1,CSNK2B,MMP2,PCDH18,PTK2,RUNX1,RUNX2,TPM1 |  |
| mir-7 |  | microrna |  |  | 2,59E-02 | BAX,KLF4,PIK3R3 |  |
| ITGAL | 6,105 | transmembrane receptor |  |  | 2,59E-02 | HEY1,ITGA4,ITGAL |  |
| ETS1 |  | transcription regulator |  | 1,264 | 2,65E-02 | CASP1,CDK2,COL2A1,MET,PEG10,PML,RUNX1,TBXAS1,TDP2,TRPC1,UBAP2L,ZEB1 |  |
| TGFBR2 |  | kinase |  |  | 2,77E-02 | ANGPTL4,BDNF,CASP1,CHRNB4,EXT2,HSD17B3,ING1,MMP2,NFIB,RBMS3,SELP |  |
| ESR2 | -6,818 | ligand-dependent nuclear receptor |  | 0,365 | 2,90E-02 | BID,MME,NDRG2,NKX3-1,PGR,RARA,SCARB1,VAV3 |  |
| CDX2 |  | transcription regulator |  |  | 2,91E-02 | AXIN2,CDH17,CLDN2,LGALS8,POU5F1,PRDM1,SOX5,TCF7L2,UGT1A7 (includes others) |  |
| AHI1 |  | other |  | -0,447 | 3,02E-02 | BCAS4,CDKN1C,GRHL1,IL1RN,SPIB |  |
| miR-122-5p (miRNAs w/seed GGAGUGU) |  | mature microrna |  | -1,000 | 3,26E-02 | ADAM17,AKT3,ANK2,ENTPD4,NFATC1,NFATC2IP,RAB6B,TPD52L2,TRIB1 |  |
| ATF6 |  | transcription regulator |  | 1,633 | 3,48E-02 | AURKA,CDKN3,DAPK1,HMMR,NUCB2,TROAP |  |
| HSPA5 |  | enzyme |  | -0,526 | 3,48E-02 | ACADM,ACADVL,APP,CLU,CPT1A,DDIT3 |  |
| APOE |  | transporter |  |  | 3,49E-02 | ADGRG1,BEGAIN,CASP1,CLN8,DIP2A,KCNAB1,NELL2,PCDH9,PRPSAP2,SEMA6B,SORBS1,TNIP1 |  |
| ESRRG |  | ligand-dependent nuclear receptor |  | -0,152 | 3,50E-02 | ALDOC,KCNQ1,PDK4,TPI1 |  |
| CSF3 |  | cytokine |  | -0,218 | 3,50E-02 | ARHGDIA,FPR1,PPARG,RARA |  |
| mir-17 |  | microrna |  |  | 3,50E-02 | AKT1,APP,FAS,POU5F1 |  |
| TPM3 |  | other |  |  | 3,64E-02 | MMP2,TEK,TNFRSF25 |  |
| ZBTB17 |  | transcription regulator |  |  | 3,64E-02 | CDKN1C,DDIT3,RRM2B |  |
| IGFBP2 |  | other |  | -1,073 | 3,67E-02 | CADM1,COL14A1,COL21A1,EPHA4,NF1,RBMS3,TGFB3 |  |
| TCF |  | group |  |  | 3,80E-02 | ADGRG2,CTNNA2,ECM1,LEF1,LMO2,MME,MMP2,NRCAM,PCSK6,SERPINA1,SERPINA3,SERPINA5 |  |
| CEBPA |  | transcription regulator |  | 1,508 | 3,81E-02 | AKAP12,CSF1R,CSF2RA,CTNNA1,DICER1,EEF1A2,EPHX1,ICAM2,IL1RN,ITGAL,NFATC2,PTPRC,SPINT2,TBXAS1,TNFSF10,TRIB1,VDR |  |
| LTB4R2 |  | g-protein coupled receptor |  |  | 3,98E-02 | MMP2,NOX4 |  |
| ZNF24 |  | transcription regulator |  |  | 3,98E-02 | CDKN3,MMP2 |  |
| miR-199a-3p (and other miRNAs w/seed CAGUAGU) |  | mature microrna |  |  | 3,98E-02 | DNMT3A,MET |  |
| RUVBL2 |  | transcription regulator |  |  | 3,98E-02 | AXIN2,PGR |  |
| IFT57 |  | other |  |  | 3,98E-02 | CASP8,REST |  |
| CANX | 8,044 | other |  |  | 3,98E-02 | BAX,NOX4 |  |
| ACTA2 |  | other |  |  | 3,98E-02 | MET,PTK2 |  |
| CCR2 |  | g-protein coupled receptor |  |  | 3,98E-02 | APP,ITGAL |  |
| TGFB1 |  | growth factor |  | 1,205 | 4,02E-02 | ANGPTL4,BACE1,BARD1,BAX,CADM1,CBFA2T3,CD59,CDK2,CDKN3,CLEC2D,COL2A1,CSF1R,ESR2,FAS,FURIN,GLI1,HEY1,ITGA4,LASP1,MAPK1,MET,MKL1,MMP2,NEK2,ORC1,POLD1,PRSS3,PTPRK,PXN,SCARB1,SCG5,SERPINA1,SERPINA3,TFAP2A,TPM1,TYMP,WNT5B,ZEB1,ZFYVE9 |  |
| OSM |  | cytokine |  | 1,783 | 4,04E-02 | ABCC5,ANGPT2,IL6R,PTGES,SELP,SERPINA1 |  |
| ATF3 |  | transcription regulator |  | -1,000 | 4,33E-02 | AURKA,AURKB,CHAC1,KAT5,NEK2 |  |
| CLU | -5,886 | other |  | -1,994 | 4,35E-02 | AKT1,BAX,CLU,MMP2 |  |
| SOX9 |  | transcription regulator |  |  | 4,35E-02 | COL2A1,LRP6,PPARG,TCF7L2 |  |
| YAP1 |  | transcription regulator |  | 1,732 | 4,72E-02 | AURKB,BAX,CASP8,DIAPH3,DICER1,MSLN,NEK2 |  |
| STAT5a/b |  | group |  | 0,816 | 4,72E-02 | EPHA4,FCMR,LRMP,PROX1,RARA,STIP1,STK17B |  |
| CBX5 |  | transcription regulator |  | -0,535 | 4,79E-02 | ALDH3A1,AXIN2,CDC25A,CDH17,GRHL3,HOXB6,ICAM2,LEF1,PPARG,PSMB8,RUNX2,SCG5,SERPINA3,SLC7A7,SYTL2 |  |
| INHBA |  | growth factor |  | -1,069 | 4,84E-02 | BAX,CDC25A,CHD7,CLASP2,ERBB4,FRS2,GRIA3,HIC2,PCDH9,POGZ,PRDM1,PRPF38B,PTBP2,VAV3 |  |
| ICAM1 |  | transmembrane receptor |  |  | 4,87E-02 | HEY1,ITGA4,ITGAL | |
| THPO |  | Cytokine |  |  | 4,87E-02 | AURKA,AURKB,BAX | |
| NR2F2 |  | ligand-dependent nuclear receptor |  |  | 4,87E-02 | POU5F1,PROX1,TFAP2A | |

**Supplemental Table S12: List of most significant upstream regulators in High fat + insulin (HFIns) treatment (p-value of overlap ≤ 0,05).**

| HIGH FAT INSULIN | | | | | | |
| --- | --- | --- | --- | --- | --- | --- |
| Upstream Regulator | Expr Log Ratio | Molecule Type | Predicted Activation State | Activation z-score | p-value of overlap | Target molecules in dataset |
| FBXW7 |  | transcription regulator |  | 1,000 | 7,91E-04 | ADAM17,DDX11,DTX1,HSPA8,HSPB1,ITGB3BP,PPARGC1A |
| FSH |  | complex |  | -0,478 | 8,70E-04 | ACPP,ACTR2,ADGRG1,ALDH3A2,AMOTL2,ATP2B1,CASP4,CDK14,CDKN1C,CREM,DAPK1,FDXR,FILIP1L,FKBP5,GK,GNAS,HSD3B1,HSD3B2,KIDINS220,LDLR,LRRC32,MAPK14,MBD2,MMP2,PLCL1,PMAIP1,PPP2R5C,PRNP,RAD17,RASSF2,RGS12,RGS5,RGS7,SGK1,STK24,VEGFA,VGF,ZNF331 |
| E2F7 |  | transcription regulator |  |  | 1,35E-03 | CCNB1,ECT2,KIF23,RACGAP1 |
| BRD7 |  | transcription regulator |  | -0,140 | 1,89E-03 | CYP1A1,DICER1,ESR1,NDRG1,PIDD1 |
| ATF6 |  | transcription regulator |  | -0,707 | 1,96E-03 | AURKA,BUB1,CDKN3,DAPK1,HERPUD1,HMMR,NUCB2,UNC13B |
| NFYA |  | transcription regulator |  |  | 2,01E-03 | APAF1,CYP1A1,GUCY1B3,IGF1,LPIN1,PIDD1,PMAIP1 |
| E2F8 | -5,384 | transcription regulator |  |  | 2,52E-03 | CCNB1,ECT2,KIF23,RACGAP1 |
| TPM3 |  | other |  | 0,000 | 4,25E-03 | IGF1,MMP2,TNFRSF25,VEGFA |
| FGF8 |  | growth factor |  | 1,265 | 4,56E-03 | CDK20,DDAH2,FGFR1,FGFR4,LAMB3,RGS12,SLC22A17,SPP1,TNXB,VDR |
| PANDAR |  | other |  |  | 5,03E-03 | APAF1,PIDD1,PMAIP1 |
| Histone h3 |  | group |  |  | 5,22E-03 | ADD2,CDK14,CIITA,COL4A6,CTNNA1,ENO3,ESR1,FGFR1,FKBP5,FMR1,FOXM1,FOXP4,HBE1,KIAA0895,MX1,NDUFV1,NRF1,POLR2A,PPP2R2B,PTGES,RARB,RBM39,RCAN1,RPS9,SBF1,SEC31A,SPATS2L,SPHK1,SRRM2,SYNE2,TERT,ZNF423 |
| AHI1 |  | other |  | 0,816 | 5,93E-03 | BCAS4,BIN1,CDKN1C,IL1RN,PALM2,SPIB |
| RNF31 |  | enzyme |  | 0,816 | 5,93E-03 | ACE,APOA1,AURKA,C8orf44-SGK3/SGK3,ESR1,NR5A2 |
| let-7 |  | microrna |  | 0,691 | 6,30E-03 | APC2,BCAT1,CDC25A,DICER1,E2F5,EIF4G2,LEF1,MYD88,PBX2,PPP1R12B,RDH10,RELA,STARD13,TARBP2,ZC3H3 |
| MAPK1 |  | kinase |  | 1,897 | 6,60E-03 | ADAM17,AURKA,BICRAL,C1S,CCNB1,CRYL1,DXO,HIC2,IFI16,L3MBTL1,MITF,NOX5,OAS1,OLFML2A,PAQR6,PLD1,PSMB8,QKI,SP110,SPAG16,TAP1,TRIM14,TRIM25,VDR,ZC3HAV1 |
| PRDM1 |  | transcription regulator |  | -0,882 | 6,61E-03 | CIITA,ESR1,PSMB8,TAPBP |
| Histone h4 |  | group |  |  | 8,53E-03 | CD247,CDC25A,CHRNA3,CIITA,ESR1,FMR1,FOXM1,PMAIP1,RARB,SPHK1,TERT |
| ERK1/2 |  | group |  | -0,380 | 9,36E-03 | ASAH1,CYP46A1,ESR1,ESR2,FKBP5,FOSB,IL17RD,IL19,MMP2,NR4A3,PSMB8,PTGES,SGK1,TAP1,TAPBP,TERT,UNC119,VEGFA,WISP1 |
| TXN |  | enzyme |  | 0,933 | 9,56E-03 | APOA1,CYP1A1,ESR1,NOS2,RELA,VEGFA |
| NUPR1 |  | transcription regulator |  | 0,135 | 1,15E-02 | ABCC5,ADGRG1,ALDOC,ALOXE3,ARHGEF26,ATP6V0A1,AURKA,BUB1,C1orf112,CARD8,CCDC77,CDK15,COQ10A,CXADR,DGCR8,DHCR24,DIDO1,E2F8,ELMOD1,ETV1,FAM111B,FGF1,FHL2,FOXO3,GCH1,GK,GNE,IL13RA1,IL6R,KIF23,LMNB1,LRP8,MSH6,MYD88,MYH10,NAPEPLD,NDRG1,NFIB,OSBPL6,PER3,PEX12,POLE2,POLH,PRNP,RBM14,SHOX2,SLC16A6,SPATS2L,SPC25,SRGAP2,ST6GALNAC6,STX3,SYNE2,USP36,WNK1,ZC3HAV1 |
| HNRNPA2B1 |  | other |  |  | 1,18E-02 | ADGRG2,CADM1,CPS1,CUX1,ELOVL7,GABRA5,HYAL1,MAST4,NR3C2,OAS1,PKIB,PLCB4,PPARGC1A,PRSS3,RIMKLB,SCN9A,SEMA3B,STK32B,SULT1A1,WWOX |
| CNOT7 |  | transcription regulator |  |  | 1,20E-02 | C12orf75,HERC6,OAS1,PPM1K,PSMB8,SP110,TAP1 |
| PRDM5 |  | transcription regulator |  | 0,000 | 1,22E-02 | CACNA1C,CKLF,CYP3A7,EBF1,EDNRA,EED,GOLGA6A (includes others),IL6R,ISLR2,MYB,MYD88,NREP,PPP1R12B,RXRB |
| ERG |  | transcription regulator |  | 0,149 | 1,27E-02 | ADGRG1,ARHGAP20,ARHGAP24,DIAPH2,DIP2A,DOCK2,HDAC6,HERPUD1,HMMR,IKBKG,LEF1,MYO1D,NPHP1,NRG1,NUMB,PTPN22,RAB7A,RAPGEF1,RGS3,TACC1 |
| Lh |  | complex |  | -0,273 | 1,36E-02 | ACPP,ACTR2,ADGRG1,ATP2B1,CASP4,CDK14,CDKN1C,DAPK1,FDXR,FKBP5,GK,GNAS,HSD3B1,HSD3B2,KIDINS220,MAPK14,MBD2,MMP2,PLCL1,PMAIP1,PPP2R5C,RGS12,RGS5,RGS7,SGK1,STK24,VEGFA |
| ESR1 | 7,553 | ligand-dependent nuclear receptor |  | -0,534 | 1,59E-02 | ABCC5,ADAM17,BAZ2A,BCL2L2,C1QTNF6,CDKN3,CLN8,CYP1A1,ESR1,FGFR1,FOXM1,ICOSLG/LOC102723996,IGF1,KIF23,LDLR,MAP2K7,MAPK12,MND1,NRF1,PHF19,PHLDB1,PKIB,POLA1,RGS19,RGS3,RNF38,SCUBE2,SEC31A,SEMA3B,SGK1,SLC25A36,SMAD3,SP110,STX3,TACC1,TAPBP,TERT,TNFRSF10A,UMODL1,UNC119,VEGFA |
| SF3B1 | 5,135 | other |  |  | 1,95E-02 | MDM4,PUF60 |
| DLX4 | 5,321 | transcription regulator |  |  | 1,95E-02 | DLX4,VEGFA |
| FBLN2 |  | other |  |  | 1,95E-02 | MMP2,VEGFA |
| miR-615-3p (miRNAs w/seed CCGAGCC) |  | mature microrna |  |  | 1,95E-02 | LCOR,PPARG |
| miR-1-3p (and other miRNAs w/seed GGAAUGU) |  | mature microrna |  |  | 1,95E-02 | ESR1,SPHK1 |
| JARID2 |  | transcription regulator |  |  | 1,95E-02 | GATA2,HOXD8 |
| ICOSLG/LOC102723996 | -5,555 | other |  |  | 1,95E-02 | FOXP3,ICOSLG/LOC102723996 |
| CCNA1 |  | other |  |  | 1,95E-02 | MMP2,VEGFA |
| SRSF5 |  | other |  |  | 1,95E-02 | NR3C1,VEGFA |
| NR3C1 | 5,039 | ligand-dependent nuclear receptor |  | -0,110 | 1,96E-02 | AIFM3,ANGPTL4,BCL2L2,BIN1,CASP4,CDKN1C,CSRNP1,DAPK2,DAXX,DEAF1,DIABLO,ELMOD1,ELMOD3,FKBP5,FOXO3,IKBKB,IL18,IL1RAP,IL24,IP6K3,MAPK7,MAX,MCF2,MDM4,MYD88,NAIP,NFATC1,NOS2,NR3C1,NTRK1,PDE4B,PIK3R3,PPP3CA,RAD21,RELA,RNF38,SEMA5B,SGK1,SLC19A2,SON,SPP1,TLR1,TNFRSF10A,TNFRSF25,UNC13B |
| FANCC |  | other |  |  | 2,06E-02 | CASK,FGFR1,IL1R1,MX1,NR3C1,OAS1,RND3,SYPL1,TOM1L1 |
| RARA |  | ligand-dependent nuclear receptor |  | 0,108 | 2,20E-02 | ADAM17,APOA1,BAZ2A,CDKN3,CLN8,FGFR1,FGFR4,FOXP3,KIF23,MND1,NTRK1,PHF19,PHLDB1,RARB,RARRES1,RGS3,RNF38,SEC31A,SLC25A36,SMAD3,SP110,SPP1,STX3,TAPBP,TNFRSF10A |
| PRKCI |  | kinase |  |  | 2,33E-02 | ECT2,ELF3,SF3B1 |
| RUNX1 |  | transcription regulator |  | -0,391 | 2,35E-02 | ADGRG1,FOXP3,GATA2,IL6R,NR4A3,NTRK1,PMAIP1,SPP1 |
| SPI1 |  | transcription regulator |  |  | 2,50E-02 | CCR6,CD72,CIITA,CSF2RB,EPB41L2,HK3,IL1RN,IL24,PSMB8,PTPN6 |
| SP1 |  | transcription regulator |  | 0,426 | 2,51E-02 | ALOX15B,CHRNA3,CHRNB4,CIITA,CREM,DIAPH2,ESR1,FOXM1,GNAS,GP6,HSD3B1,LDLR,LIPA,MMP2,MSH6,NTRK1,PMAIP1,PROM1,PTH1R,RELA,SLC19A1,SMAD3,SNCG,SPP1,SRC,TERT,TIMP2,TP73,UGT1A7 (includes others),VEGFA,ZEB2 |
| TCF4 |  | transcription regulator |  |  | 2,65E-02 | FGF1,LEF1,NOS2,SGK1,SPP1,TCF7L2,TERT |
| TP53 |  | transcription regulator |  | -1,675 | 2,84E-02 | AMOTL2,APAF1,AURKA,BID,BUB1,CASP4,CASP8,CCNB1,CCNL1,CDC25A,CDKN3,DAPK1,DBP,DGKA,DICER1,DLGAP1,ESR1,FDXR,FOXP3,GLS2,HMMR,HSPA4L,HSPA8,IGF1,ISCU,KIF23,LIPF,LPIN1,MAP2K7,MAP3K8,MAP4,MAPK12,MDM4,MLH1,MMP2,MSH6,NDRG1,NOS2,OAS1,PANK1,PCBP4,PDE4B,PFKM,PIDD1,PMAIP1,POLE2,PPP3CA,PPP4C,PRNP,PROM1,PTPN6,RAD17,RPS6KB1,RRM2B,SCN3B,SCP2,SELP,SLC19A1,SON,SRC,SRGAP3,SULF2,TAP1,TCF7L2,TERT,TNFRSF10A,TP73,TPD52L1,VEGFA |
| SRF |  | transcription regulator |  |  | 2,93E-02 | ANAPC15,ARHGAP20,EGR2,EPG5,FGF1,FHL2,FILIP1L,FOSB,HIPK1,MBD2,NLRP1,NR4A1,RND3 |
| ESRRG |  | ligand-dependent nuclear receptor |  | 1,980 | 3,08E-02 | ALDOC,KCNE1,KCNQ1,PDK4 |
| ZBTB17 |  | transcription regulator |  |  | 3,28E-02 | CDKN1C,PMAIP1,RRM2B |
| SIRT1 |  | transcription regulator |  | 1,709 | 3,40E-02 | CYP1A1,FOXO3,IGF1,LDLR,MMP2,NAT1,TERT,TP73 |
| Cg |  | complex |  | -1,332 | 3,40E-02 | ACPP,EPHB6,ESR2,HSD3B1,ITGA4,MMP2,NR1H2,NRIP1,PMAIP1,RGS20,SACS,STC1,TRO,VEGFA |
| OSM |  | cytokine |  | 0,028 | 3,42E-02 | ABCC5,IL6R,LDLR,PTGES,SELP,VEGFA |
| IFNG |  | cytokine | Inhibited | -2,746 | 3,55E-02 | ADORA2A,BCL2L2,CABP7,CASP8,CHAC1,CIITA,DAPK1,DUOX2,EFCAB6,FCER1G,GCH1,HERC6,ICOSLG/LOC102723996,IL1RN,IL32,MCHR1,MMP2,MOV10L1,MTMR3,MX1,MYD88,NOS2,OAS1,PMAIP1,PSMB8,PTGES,RARRES1,SBF1,SELP,SEPT4,SLC2A11,TAB1,TAP1,TCF7L2,TLR1,TP73 |
| MEF2 |  | group |  |  | 3,69E-02 | IL1RN,NR4A1 |
| HCAR1 |  | g-protein coupled receptor |  |  | 3,69E-02 | BSG,PPARGC1A |
| ZNF24 |  | transcription regulator |  |  | 3,69E-02 | CDKN3,MMP2 |
| HDAC10 | -6,818 | transcription regulator |  |  | 3,69E-02 | MMP2,NR4A1 |
| miR-515-5p (and other miRNAs w/seed UCUCCAA) |  | mature microrna |  |  | 3,69E-02 | PIK3C2B,SPHK1 |
| miR-503-5p (miRNAs w/seed AGCAGCG) |  | mature microrna |  |  | 3,69E-02 | CDC25A,FGFR1 |
| IRS2 |  | enzyme |  |  | 3,69E-02 | MX1,VEGFA |
| HDAC9 |  | transcription regulator |  |  | 3,69E-02 | CCNB1,NR4A1 |
| NKX2-3 |  | transcription regulator |  |  | 3,69E-02 | AOC3,SHOX2 |
| PTH |  | other |  |  | 3,69E-02 | CSF1,PTH1R |
| ISG15 |  | other |  |  | 3,69E-02 | MX1,OAS1 |
| RBCK1 |  | transcription regulator |  |  | 3,69E-02 | CCNB1,ESR1 |
| DEFB103A/DEFB103B |  | other |  |  | 3,69E-02 | BID,IL18 |
| ADORA3 |  | g-protein coupled receptor |  | 0,000 | 3,84E-02 | CSF1,IL24,SPP1,VEGFA |
| PIK3CA |  | kinase |  |  | 3,84E-02 | CCNB1,FOXM1,IL19,NOS2 |
| SOX9 |  | transcription regulator |  |  | 3,84E-02 | DMBT1,MITF,PPARG,TCF7L2 |
| YAP1 |  | transcription regulator |  | -0,351 | 3,94E-02 | AMOTL2,CASP8,DICER1,FOXM1,MSLN,PMAIP1,TP73 |
| CREBBP |  | transcription regulator |  |  | 3,94E-02 | FOSB,GNAS,NR4A1,NR4A3,RARB,SERPINE2,SMPD3 |
| RABL6 |  | other |  | 0,632 | 4,09E-02 | BUB1,CCNB1,CDC25A,DAPK1,EZH2,HMMR,KIF23,PMAIP1,POLA1,POLE2 |
| SMARCA4 |  | transcription regulator | Inhibited | -2,168 | 4,18E-02 | ADGRG1,AP1S2,ATP2B4,C1orf54,CCR6,CD163L1,CDC25A,CIITA,CLK1,CPM,CPS1,EDNRA,ENTPD3,ESPNL,FHL2,FKBP5,HBE1,HCLS1,HCST,HENMT1,HEPH,IFI16,IGF1,LRAT,MARCH1,MCTP2,MMP2,NECTIN1,NFKBIZ,NOSTRIN,NRP2,PDE4B,PER3,PHLDB1,SEMA3B,SERPINE2,SPHK1,SPP1,STXBP6,TAOK3,TAP1,TBX15,TM4SF19,TMEM117,TRIM36,TWF1,WDR45 |
| NFKB1 |  | transcription regulator |  |  | 4,34E-02 | CIITA,DICER1,ELF3,FOSB,IL1RN,IL20,MYB,NOS2,NR4A1,NR4A3,PTPN6,TERT,VEGFA |
| GPER1 |  | g-protein coupled receptor |  |  | 4,40E-02 | ASAH1,CACNA1D,ESR1 |
| TRIM28 |  | transcription regulator |  |  | 4,40E-02 | DNAJC6,MMP2,ZNF274 |
| PRAME |  | other |  |  | 4,40E-02 | EIF2AK3,IL13RA1,RARB |
| PIM2 |  | kinase |  |  | 4,40E-02 | CDC25A,CDKN1C,TP73 |
| LIPG |  | enzyme |  |  | 4,40E-02 | CSF1,LDLR,VEGFA |
| SFN |  | other |  | -0,447 | 4,41E-02 | AMOT,FKBP5,IL1R1,PDK4,SGK1 |
| KMT2D |  | transcription regulator |  | -0,492 | 4,41E-02 | ADGRG1,ADGRG2,ENO3,LAMB3,PPP2R2B |
| HNF4A |  | transcription regulator |  | 0,293 | 4,48E-02 | APOA1,CYP1A1,CYP2B6,IGF1,NPC1L1,NR1H2,NR1H3,PPARG,RARB,RARG,UGT1A7 (includes others) |
| HDAC1 |  | transcription regulator |  | -0,070 | 4,48E-02 | APAF1,CCNB1,CDC25A,ESR1,FOXM1,GNAS,PMAIP1,PPP2R2B,SPP1,TERT,TP73 |
| USP7 |  | peptidase |  | -1,000 | 4,70E-02 | FAM111B,FKBP5,FOXP3,GK |
| TGFBR2 |  | kinase |  | -1,000 | 4,89E-02 | ANGPTL4,CHRNB4,COL4A6,HSD17B3,MMP2,NFIB,PDPN,SELP,UBE3A,ZMIZ1 |
